# Development of an efficient screening system to identify novel bone metabolism-related genes using the exchangeable gene trap mutagenesis mouse models

**DOI:** 10.1038/srep40692

**Published:** 2017-01-20

**Authors:** Syuji Kurogi, Tomohisa Sekimoto, Taro Funamoto, Tomomi Ota, Shihoko Nakamura, Takuya Nagai, Mai Nakahara, Kumiko Yoshinobu, Kimi Araki, Masatake Araki, Etsuo Chosa

**Affiliations:** 1 Division of Orthopaedic Surgery, Department of Medicine of Sensory and Motor Organs, Faculty of Medicine, University of Miyazaki, Japan; 2 Institute of Resource Development and Analysis, Kumamoto University, Japan

## Abstract

Despite numerous genetic studies on bone metabolism, understanding of the specific mechanisms is lacking. We developed an efficient screening system to identify novel genes involved in bone metabolism using mutant mouse strains registered with the Exchangeable Gene Trap Clones (EGTC) database. From 1278 trap clones in the EGTC database, 52 candidate lines were selected in the first screening, determined based on “EST profile”, “X-gal”, “Related article”, and “Novel gene”. For the second screening, bone morphometric analysis, biomechanical strength analysis, bone X-gal staining, etc. were performed on candidate lines. Forty-two male trap lines (80.8%) showed abnormalities with either bone morphometric analysis or biomechanical strength analysis. In the screening process, X-gal staining was significantly efficient (P = 0.0057). As examples, *Lbr* and *Nedd4* trap lines selected using the screening system showed significant bone decrease and fragility, suggesting a relationship with osteoblast differentiation. This screening system using EGTC mouse lines is extremely efficient for identifying novel genes involved in bone metabolism. The gene trap lines identified as abnormal using this screening approach are highly likely to trap important genes for bone metabolism. These selected trap mice will be valuable for use as novel bio-resources in bone research.

Osteoporosis is a serious metabolic bone disease, affecting an increasing number of patients owing to the aging populations worldwide. In Europe, the number of osteoporosis patients is reported to be 27.5 million, with 37 billion Euros spent annually on treatment^1^. The population of Japan is also aging rapidly, with Yoshimura *et al*. estimating the number of osteoporosis patients in Japan at over 12.8 million^2^. The Japanese Orthopaedic Association has defined conditions wherein mobility functions are declined due to locomotive organ impairment, including osteoporosis and osteoarthritis, as “locomotive syndrome”^3^, and is proactively committed to taking preventive measures and promoting awareness of locomotive syndrome.

For the prevention and treatment of osteoporosis, a better understanding of bone metabolism is essential. Until now, many genes involved in bone metabolism and their functions have been identified, such as runt-related transcription factor 2 (*Runx2*)^4^, old astrocyte specifically-induced substance (*OASIS*)^5^, and receptor activator of nuclear factor kappa-B ligand (*RANKL*)^6^. Currently, the anti-RANKL antibody denosumab (PRALIA^®^) is used for the clinical treatment of osteoporosis and has contributed to fracture prevention. Understanding bone metabolism is helpful for the prevention and treatment of osteoporosis, but more remains to be understood.

Complete genome sequences of individual organisms have been mapped, as seen with the completion of the Human Genome Project^7^. However, base sequence information alone is not enough to extrapolate all the biological functions of the encoded genes and other parts of the coding regions. Furthermore, many proteins show diverse functions; for examples, Runx2 (Cbfa1) was originally identified as a T lymphocyte-specific transcriptional regulator^4^, OASIS as a transcriptional factor in astrocytes and gliotic tissue^8^, and RANKL as a growth factor of T-cells^6^. Therefore, analysis of genetically modified mice *in vivo* is an essential method to determine the functions of novel genes, or the unknown functions of known genes, to improve the annotation of our genome sequences.

Co-authors Araki *et al*. developed an exchangeable gene trap method^9^,^10^,^11^ and have disclosed trap clone information on the Exchangeable Gene Trap Clones (EGTC) database (http://egtc.jp)^12^,^13^. Information on 1278 trapped embryonic stem (ES) cell clones has been registered with the EGTC database (as of September 2016), and 490 gene trap lines have been extensively established from trapped ES clones, which allows us to easily search a target trap gene in the database.

To date, little has been reported on a screening method using mutant mouse strains to identify causative molecules in metabolic bone disease^14^. In the current study, we report on the development of an efficient screening system to identify novel genes involved in bone metabolism using mutant mouse strains established with the exchangeable gene trap method based on the EGTC database (Fig. 1a).

## Results

### First screening

In the first screening, 52 trap lines that were considered novel bone metabolism-related trap genes were selected from the EGTC database. Although the EGTC database includes important known genes related to bone metabolism, trapped genes that have been studied enough and already linked to bone metabolism *in vivo* were excluded from the first screening. Table 1 shows gene trap line information selected in the first screening. A total of 52 trap lines for the second screening were determined as follows: 20 lines from “Expressed Sequence Tag (EST) profile”, eight lines from “Novel gene”, seven lines from “Related article (RA)” (Supplementary Table S1), four lines from “X-gal”, 10 lines from both “EST profile” and “X-gal”, two lines from “X-gal” and “Novel gene”, and one line from “RA” and “Novel gene”. Eleven lines are “novel genes with no prior mutant mice (NPM)” and 41 lines are “known genes with a prior mutant mouse, but no bone phenotype reported (NBR)” (Table 1).

### Second screening

To discover novel genes involved in bone metabolism *in vivo*, the 52 gene trap lines selected in the first screening (Table 1) were investigated in the second screening. Homozygous (Homo) mice were used for all screenings, but when these were not available, because of embryonic lethality, heterozygous (Hetero) mice were used and analysed with wild-type (WT) littermate mice. Analyses of growth curves, biochemical blood tests, micro-computed tomography (μCT), bone morphometric analyses (BMA), biomechanical strength analyses (BSA), and bone X-gal staining were performed in the second screening. A case was determined as “positive” when at least one parameter was >2.0 standard deviations (SD) from the WT mean [WT mean ± 2.0 SD].

### Growth curve and biochemical blood test

**Figure 1:**
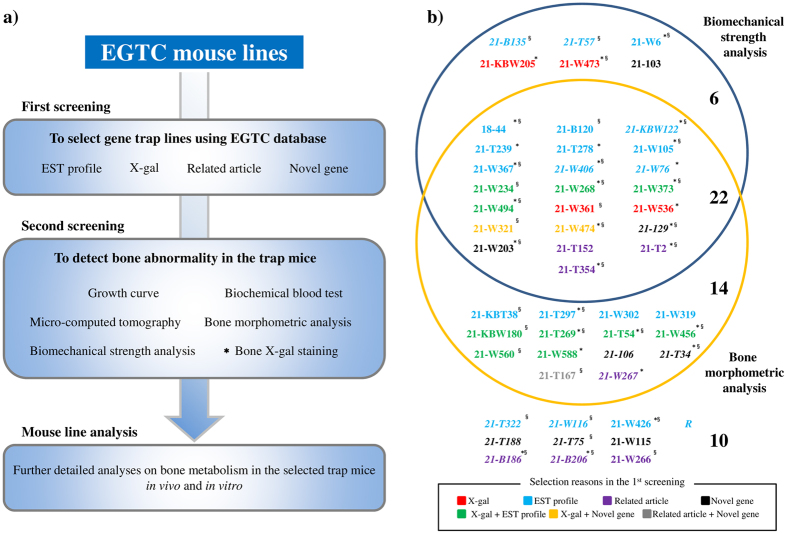
(**a**) Screening system outline. EST: Expressed Sequence Tag, *X-gal staining was performed in detail only on bone in the second screening. (**b**) Biomechanical strength analysis and bone morphometric analysis results from male trap lines in the second screening. Lines inside and outside the frame indicate those with and without an observed difference in the second screening, respectively. Selection reasons in the first screening are indicated by colour. Bold and italic fonts indicate positive and negative X-gal staining in the second screening, respectively. *A positive line in the growth curve, ^§^A positive line in the biochemical blood test (Supplementary Table S2).

Body weight measurements from 2 to 10 weeks after birth and biochemical blood tests were performed for the 52 lines selected from the first screening. In male lines, six lines (11.5%) showed a positive weight increase, and 24 lines (46.2%) showed a positive weight decrease. Thirty-eight lines (73.1%) were positive following biochemical blood tests and 45 lines (86.5%) were positive for either body weight measurements or following biochemical blood tests (Table 2, Fig. 1b, Supplementary Table S2). In female lines, eight lines (15.4%) showed a positive weight increase, and 25 lines (48.1%) showed a positive weight decrease. Twenty-six lines (50.0%) were positive following biochemical blood tests (Supplementary Table S2).

### Micro-computed tomography

μCT was performed in all 52 trap lines. Marked morphological abnormalities, such as osteogenesis imperfecta, anomalies, and pathological fractures, were not observed in any of the 52 lines (data not shown).

### Bone morphometric analysis

BMA was performed in the 52 trap lines selected from the first screening. Thirty-six male lines (69.2%) were positive in bone mineral density (BMD) analysis, cortical bone analysis, or trabecular bone analysis. Twelve (23.1%) were positive in both trabecular bone analysis and cortical bone analysis, and only three lines (5.8%) were positive in cortical bone analysis alone (Ayu21-W105/Ayu21-W373/Ayu21-KBW180) (Table 2, Fig. 1b, Supplementary Table S2). Thirty-six (69.2%) female lines were positive in BMA, and six lines (11.5%) were positive in both trabecular bone analysis and cortical bone analysis (Supplementary Table S2).

### Biomechanical strength analysis

BSA was performed for the 52 lines selected from the first screening. Twenty-eight male lines (53.8%) were positive for maximum load (M.load), maximum stress (M.stress), maximum displacement (M.dip), maximum work (M.work), or elastic modulus (E.M). For M.load, which is an indicator of bone strength, greater bone strength was observed in 11 lines (21.2%) compared with WT mice, while lesser bone strength was observed in 17 lines (32.7%). For E.M, which is an indicator of the flexibility of bone, 11 lines (21.2%) were positive, and of these, three lines (27.3%) showed an increase in male trap mice (Ayu21-W367/Ayu21-W321/Ayu21-W203) (Table 2, Fig. 1b, Supplementary Table S2). Thirty-one female lines (59.6%) were positive in BSA. Eleven lines (21.2%) showed greater bone strength compared with WT mice, and 20 lines (38.5%) showed lesser strength (Supplementary Table S2).

These results demonstrated that 42 male (80.8%) and 44 female (84.6%) trap lines showed abnormalities in either BMA or BSA (Table 2, Supplementary Table S2).

### Bone X-gal staining

Because the EGTC database does not include the results of bone X-gal staining in all trap lines, X-gal staining was performed on femurs of all lines in the second screening. WT mice of all 52 trap lines were compared for use as controls. X-gal staining was observed in trabecular bone or cartilage of the femoral head in 36 lines (69.2%), while staining was not observed in 16 lines (30.8%) compared with WT mice (Tables 2 and 4b, Supplementary Table S2).

### Correlation between the first screening and second screening

The usefulness of the first screening was evaluated for the discovery of novel genes involved in bone metabolism using the results of BMA and BSA in the second screening, which directly reflect bone metabolism. The correlation between the first screening and the second screening was investigated. In male trap lines, “X-gal” was significantly efficient in the first screening (P = 0.0161) (Table 3). Femoral bone X-gal staining in the second screening was positive in 36 male trap lines (69.2%), and BMA or BSA were positive in 33 (91.7%) of these lines (P = 0.0057) (Table 4). As the second screening results from male samples were more significant than female samples (Supplementary Table S3), we have presented the results from the male trap lines in the following analyses.

### Classification by bone morphometric analysis and biomechanical strength analysis

From the results of the second screening in male lines, trap lines which showed positive in BMA and BSA were classified (Fig. 1b). Twenty-two lines (42.3%) were positive in both BMA and BSA, six lines (11.5%) were positive only in BSA, 14 lines (26.9%) were positive only in BMA, and 42 lines (80.8%) were positive in either analysis.

### Correlation between maximum load and bone morphometric parameters

To investigate the correlation between bone strength, which is the most important parameter for abnormality in bone metabolism, and bone morphometric parameters, a correlation coefficient was obtained using the gene trap mouse and WT mouse ratio (GT/WT) of M.load and GT/WT of bone morphometric parameters for all lines (Supplementary Table S4). As expected^15^, the BMD parameters (trabecular bone mineral density (Tb.BMD), trabecular bone mineral content (Tb.BMC), and cortical bone mineral content (Ct.BMC)) showed high correlation coefficients of 0.595 to 0.730. Cortical bone parameters (cortical volume (Ct.V), cortical thickness (Ct.Th), and external line length (Ex.Ln.Le)) also showed high correlation coefficients of 0.559 to 0.705. Although trabecular bone parameters showed high correlation coefficients for trabecular bone volume (BV), trabecular thickness (Tb.Th), and trabecular number (Tb.N), the correlation coefficient was relatively low for marrow space star volume (V*m.space).

### Correlation between maximum load and cortical bone mineral content

**Figure 2:**
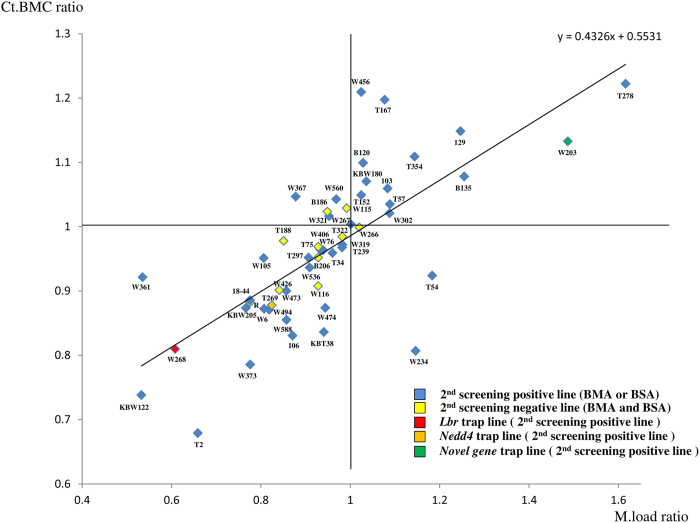
Correlation of cortical bone mineral content (Ct.BMC) and maximum load (M.load) ratios. The abscissa of the scatter diagram shows the gene trap and wild-type mouse ratio (GT/WT) of M.load of biomechanical strength analysis, and the ordinate represents the GT/WT of the Ct.BMC of bone morphometric analysis. BMA: bone morphometric analysis, BSA: biomechanical strength analysis. Each EGTC ID is indicated around the plotted point.

From the results in Supplementary Table S4, a correlation was identified as a regression line by plotting GT/WT of M.load and Ct.BMC (correlation coefficient 0.730; P = 3.25 × 10^−9^), typical parameters of BSA and BMA, on the scatter diagram (Fig. 2). A correlation was observed between M.load ratio and Ct.BMC ratio as expected^15^, and those lines negative in BMA and BSA were plotted around the reference point.

### First and second screening results of Ayu21-W268, Ayu21-T269, and Ayu21-W203

**Figure 3:**
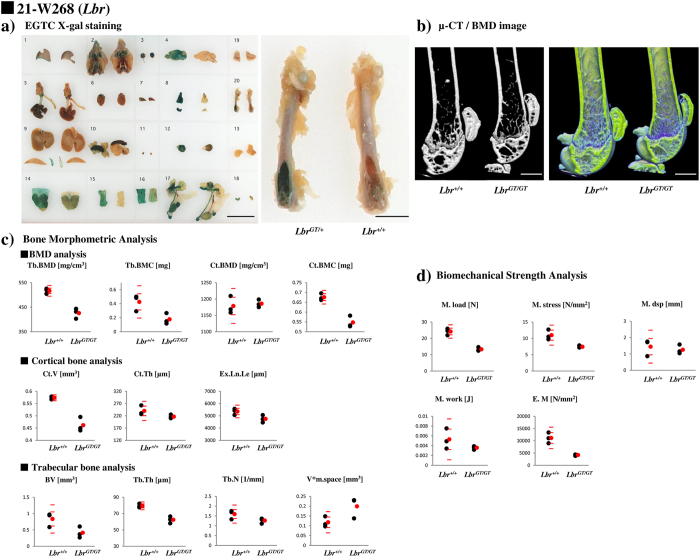
First and second screening results of Ayu21-W268. (**a**) Left: X-gal staining results in the first screening of the Ayu21-W268 (Lamin B receptor (*Lbr*) gene trap mouse line) in the EGTC. (1) ear, (2) cerebral base, (3) eye, (4) brain, (5) tongue-lung, oesophagus, (6) heart, (7) lung (slice), (8) thymus, (9) liver, (10) spleen, pancreas, (11) adrenal gland, (12) kidney, (13) muscle, (14) stomach, (15) duodenum, (16) rectum, (17) genital tract, adipose tissue, (18) bladder, (19) brown adipose tissue, (20) femur. Bar: 20 mm; left: heterozygote, right: negative control. Right: X-gal femoral staining in the first screening. Bar: 4 mm (http://egtc.jp). (**b**) Micro-computed tomography (μCT) image in the second screening. Left: CT image, right: bone mineral density (BMD) image. Colour scale indicates BMD; 100 mg/cm^3^ (blue) and 1550 mg/cm^3^ (yellow). Bar: 1 mm. (**c**) Graphs showing the results of bone morphometric analysis. Tb.BMD: trabecular bone mineral density, Tb.BMC: trabecular bone mineral content, Ct.BMD: cortical bone mineral density, Ct.BMC: cortical bone mineral content, Ct.V: cortical bone volume, Ct.Th: cortical bone thickness, Ex.Ln.Le: external line length, BV: trabecular bone volume, Tb.Th: trabecular bone thickness, Tb.N: trabecular bone number, V*m.space: marrow space star volume. (**d**) Three-point bending test results on femurs of *Lbr*^+/+^ and *Lbr*^*GT/GT*^ mice in the second screening. M.load: maximum load, M.stress: maximum stress, M.dsp: maximum displacement, M.work: maximum work, E.M: elastic modulus. Black points show each value, red points show mean of *Lbr*^+/+^ and *Lbr*^*GT/GT*^ mice, and red bars show 1.0 and 2.0 SD of *Lbr*^+/+^ mice.

**Figure 4:**
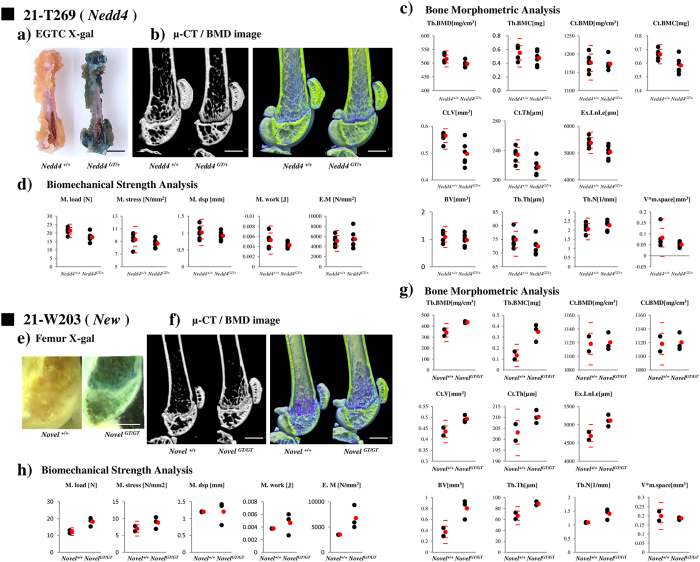
First and second screening results of Ayu21-T269 and Ayu21-W203. (**a**) Femur X-gal staining results in the first screening of Ayu21-T269 (Neural precursor cell expressed, developmentally down-regulated gene 4 (*Nedd4*) gene trap mouse line) in the EGTC database. Left: negative control, right: heterozygote. Bar: 2 mm (http://egtc.jp). (**b**) Micro-computed tomography (μCT) image in the second screening. Left: CT image, right: BMD image. Colour scale indicates BMD; 100 mg/cm^3^ (blue) and 1550 mg/cm^3^ (yellow). Bar: 1 mm. (**c**) Graphs showing the results of bone morphometric analysis. (**d**) Three-point bending test results on femurs of *Nedd4*^*+/+*^ and *Nedd4*^*GT/+*^ mice in the second screening. (**e**) Femur X-gal staining results in the second screening of Ayu21-W203 (*Novel gene* trap mouse line). Left: negative control, right: homozygote. Bar: 1 mm. (**f**) μCT image in the second screening. Left: CT image, right: BMD image. Colour scale indicates BMD; 100 mg/cm^3^ (blue) and 1550 mg/cm^3^ (yellow). Bar: 1 mm. (**g**) Graphs showing the results of BMA in the second screening. (**h**) Three-point bending test results on femurs of *Novel*^*+/+*^ and *Novel*^*GT/GT*^ mice in the second screening. Black points show each value, red points show mean, and red bars show 1.0 and 2.0 SD.

As examples of the trap lines selected by the first and second screening, the screening results of Ayu21-W268, Ayu21-T269, and Ayu21-W203 lines are presented in Figs 3 and 4. Ayu21-W268 (red in Fig. 2), Ayu21-T269 (orange in Fig. 2), and Ayu21-W203 lines (green in Fig. 2) were all distinct from the reference point in Fig. 2.

Ayu21-W268 is a Lamin B receptor (*Lbr*) gene trap line. Trabecular bone was strongly stained with “X-gal” in the first screening (Fig. 3a), and “EST profile” showed a high value of 615 in bone tissue (Table 1). In the second screening of male mice, decreased body weights were recorded at all measurement points, and a low alkaline phosphatase (ALP) value was detected with biochemical blood tests (Table 2, Supplementary Table S2). Additionally, there was loss of trabeculae on μCT images, decreased bone density of the primary spongiosa around the growth plate on BMD images (Fig. 3b), and a number of bone morphometric parameters >[WT mean ± 2.0 SD], including Tb.BMD, Tb.BMC, Ct.BMC, Ct.V, Ex.Ln.Le, Tb.Th, and V*m.space (Fig. 3c). BSA also indicated strength parameters >[WT mean ± 2.0 SD] for M.load, M.stress, and E.M (Fig. 3d). Bone X-gal staining was consistent with that of the first screening (data not shown).

Ayu21-T269 is a neural precursor cell expressed, developmentally down-regulated gene 4 (*Nedd4*) gene trap line. Cortical and trabecular bones and the femoral head were strongly stained with “X-gal” in the first screening (Fig. 4a), and “EST profile” showed a high value of 835 in bone tissue (Table 1). In the second screening of male mice, decreased body weight at 10 weeks of age and a high ALP value were detected (Table 2, Supplementary Table S2). Additionally, loss of trabeculae on μCT images, decreased bone density around the growth plate on BMD images (Fig. 4b), and bone morphometric parameters (Ct.BMC and Ct.V) > [WT mean ± 2.0 SD] (Fig. 4c) were observed. Bone X-gal staining was consistent with that of the first screening (data not shown).

Ayu21-W203 is a “Novel gene” trap line. This line was selected from “New” in the first screening (Table 1). In the second screening of male mice, decreased body weight was recorded at 10 weeks of age, and high K and Mg values and a low Cl value were detected with biochemical blood tests (Table 2, Supplementary Table S2). Additionally, overgrowth of trabeculae on μCT images, increased bone density of the primary spongiosa around the growth plate on BMD images (Fig. 4f), and a number of bone morphometric parameters >[WT mean ± 2.0 SD], including Tb.BMD, Tb.BMC, Ct.V, Ex.Ln.Le, BV, Tb.Th, and Tb.N, were observed(Fig. 4g). BSA also indicated strength parameters >[WT mean ± 2.0 SD] for M.load, M.work, and E.M (Fig. 4h). Trabecular bone around the growth plate of the femur was strongly stained with X-gal (Fig. 4e).

### Mouse line analyses of Ayu21-W268 and Ayu21-T269 trap lines after screening

To verify the usefulness of our screening system, the results of mouse line analyses of Ayu21-W268 (*Lbr*) and Ayu21-T269 (*Nedd4*) trap mice are presented as examples of representative lines. These trapped genes have not been previously reported to be associated with bone metabolism.

**Figure 5:**
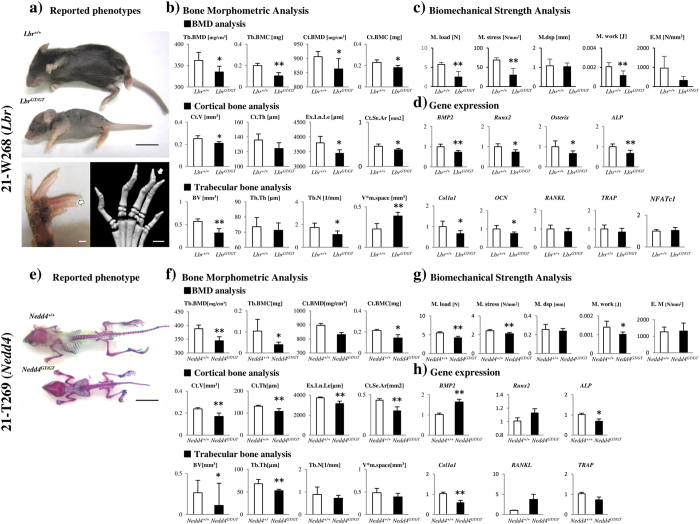
Mouse line analyses of Ayu21-W268 and Ayu21-T269 trap lines after screening. (**a**) Upper: *Lbr*^+/+^ and *Lbr*^*GT/GT*^ mice at 4 weeks old. The *Lbr*^*GT/GT*^ mouse shows sparseness of hair, presence of scales most predominantly on the tail, and decreased body size. Bar:10 mm. Lower: photo and micro-computed tomography (μ-CT) images of hind paw. The *Lbr*^*GT/GT*^ mouse shows syndactyly characterized by soft tissue fusion between digits three and four (arrows). Bar: 1 mm. (**b**) Three dimensional structural indices of femurs of *Lbr*^+/+^ and *Lbr*^*GT/GT*^ mice at 4 weeks old in bone morphometric analysis. (**c**) Three-point bending test results on femurs of *Lbr*^+/+^ and *Lbr*^*GT/GT*^ mice at 4 weeks old. (**d**) Real-time PCR analysis of bone metabolism-related genes of femurs of *Lbr*^+/+^ and *Lbr*^*GT/GT*^ mice at 4 weeks old. (**e**) Transparent skeletal specimen of *Nedd4*^+/+^ and *Nedd4*^*GT/GT*^ mice at 4 weeks old. *Nedd4*^*GT/GT*^ mice show decreased body size. Bar: 10 mm. (**f**) Three dimensional structural indices of femurs of *Nedd4*^*+/+*^ and *Nedd4*^*GT/GT*^ mice at 4 weeks old in bone morphometric analysis. (**g**) Three-point bending test results on femurs of *Nedd4*^*+/+*^ and *Nedd4*^*GT/GT*^ mice at 4 weeks old. (**h**) Real-time PCR analysis of bone metabolism-related genes of femurs of *Nedd4*^*+/+*^ and *Nedd4*^*GT/GT*^ mice at 4 weeks old. Relative expression levels were normalized to the ratio of an internal *β-actin* standard and wild-type mouse gene expression levels. Results are mean ± SD (n = 5). Student’s *t*-tests: *P < 0.05, **P < 0.01.

*Lbr* trap mice showed decreased body size, sparseness of hair, scaly skin (ichthyosis) most predominantly on the tail, and syndactyly characterized by soft tissue fusion, consistent with previous reports (Fig. 5a)^16^,^17^. *Lbr* trap mouse line analysis confirmed a significant decrease in bone volume for almost all parameters of BMD analysis, cortical bone analysis, and trabecular bone analysis in BMA, and also indicated significant bone fragility in BSA (Fig. 5b,c). Real-time PCR showed that although the osteoclast differentiation markers nuclear factor of activated T-cells, cytoplasmic 1 (*NFATc1*), and tartrate-resistant acid phosphatase (*TRAP*) were similarly expressed compared with WT mice, expression of the osteoblast differentiation markers collagen, type I, alpha 1 (*Col1a1*), *ALP,* and osteocalcin (*OCN*) significantly decreased compared with WT mice (Fig. 5d).

Similarly, *Nedd4* trap mice showed decreased body size and frequent embryonic lethality, consistent with previous reports (Fig. 5e)^18^,^19^. *Nedd4* trap mouse line analysis confirmed a significant decrease in bone volume for almost all parameters in BMD analysis, cortical bone analysis, and trabecular bone analysis in BMA, and also indicated significant bone fragility in BSA (Fig. 5f,g). Real-time PCR showed that expression of the osteoblast differentiation markers *Col1a1* and *ALP* significantly decreased compared with WT mice (Fig. 5h).

## Discussion

Bone remodelling involves a delicate balance between bone formation by osteoblasts and bone resorption by osteoclasts^20^. A number of molecules are either directly or indirectly related with the pathology of osteoporosis, but not all have been identified. In the current study, to search for novel genes involved in bone metabolism in the first screening, 52 gene trap lines (Table 1) likely to be associated with bone metabolism were selected from mouse lines registered with the EGTC database^12^,^13^. The list of trapped genes in Table 1 presents those that have not been previously reported to be associated with bone metabolism *in vivo*. Using these gene trap mice, multiple parameters were used to evaluate bone phenotypes, such as the presence of skeletal/morphometric abnormalities and bone strength, in the second screening (Table 2, Supplementary Table S2). There were 45 trap lines (86.5%) in male mice and 44 lines (84.6%) in female mice that were positive for either body weight measurements or following biochemical blood tests (Table 2, Fig. 1b, Supplementary Table S2), indicating that the first screening method was useful for the detection of abnormal body weight and biochemical blood test results. For BMA and BSA in the 52 lines selected from the first screening, 42 male (80.8%) and 44 female (84.6%) trap lines showed abnormalities in either BMA or BSA (Table 2, Fig. 1b, Supplementary Table S2). Based on these findings, we considered our screening system (Fig. 1a) to be very efficient.

Osteoporosis was defined as “a skeletal disorder characterized by compromised bone strength predisposing a person to an increased risk of fracture” at a 2001 National Institute of Health consensus conference, and after the concept of bone quality was introduced, was further defined as “bone strength primarily reflects the integration of bone density and bone quality”^21^. Saito *et al*. defined bone quality by material characteristics and structural characteristics^22^. In this screening, a relatively simple and efficient system for the selection of abnormal bone metabolism mouse lines indicating various phenotypes was developed using several screening approaches.

In this study, screening was performed using the EGTC database, which was developed by co-authors Araki *et al*. The principle behind gene trapping involves randomly inserting the trap vector containing a drug-resistant gene into the ES cell genome and disrupting only those genes with the inserted vector. This method has better efficiency for novel gene-mutant mouse production compared with other methods. An improved “exchangeable gene trap method” is characterized by: (1) trapped genes can be completely disrupted with a high frequency of the trap vector being inserted around the start codon; and (2) by applying the Cre/*lox* site-specific recombination system, the reporter gene can be replaced by any gene, which is applicable to the Cre-expressing mouse^11^,^13^. Data on trapped clones are available in the EGTC database, and established mouse lines are available for almost all registered clones. As at September 2016, 1278 gene trap clones have been isolated. Among the trap clones, novel genes account for 5.9% of clones, and known genes for 89.2%. Although the known genes include important genes related to bone metabolism, those trapped genes that have been studied enough and previously reported to be associated with bone metabolism *in vivo* were excluded from the first screening in the current study, such as Ayu21-KBW225 (BMP receptor, type 1A: *Bmpr1a*)^23^, Ayu21-KBW114 (signal transducer and activator of transcription 3: *Stat3*)^24^, Ayu21-W71 (protein inhibitor of activated STAT 2: *Pias2*)^25^, Ayu21-T320 (Tnf receptor-associated factor 3: *Traf3*)^26^, and Ayu21-KBW156 (GLI-Kruppel family member GLI2: *Gli2*)^27^. Therefore, it is expected that a large number of unknown important genes involved with bone metabolism will be included in the EGTC database.

Bassett *et al*. reported nine novel genes related to bone metabolism from 100 randomly selected lines of gene-knockout mice from the Wellcome Trust Sanger Institute Mouse Genetics Project, and their method was determined to be extremely efficient^14^. In the present study, we selected 52 gene trap lines in the first screening that were considered to have high bone tissue specificity using “EST profile”, “X-gal”, “Related article”, and “Novel gene”. Abnormalities were confirmed in 42 male (80.8%) and 44 female (84.6%) trap lines in the second screening (BMA or BSA) (Tables 1 and 2, Fig. 1b, Supplementary Table S2).When male lines were compared for second screening results and selection reasons in the first screening (Table 3), “EST profile”, “Related article”, and “Novel gene” were noted to be similar for lines that were negative in BMA and BSA, but all lines selected by “X-gal” in the first screening were positive in either BMA or BSA in the second screening. Statistical analyses of correlations between results of the first and second screenings (BMA or BSA) indicated that “X-gal” was significantly efficient (P = 0.0161) (Table 3). This is the first time that this correlation has been reported. This may be because *β-geo* is used in the trap vector, and if X-gal staining is positive in bone, it indirectly indicates that the trapped endogenous gene is expressed in bone^11^. Examining the tissue specificity of trapped genes using “X-gal” staining of bone in the first screening is considered effective. If “X-gal” staining of bone had been more frequently performed in the first screening, it is thought that better results would have been achieved. In fact, bone X-gal staining in the second screening was positive in 36 trap lines (69.2%), and BMA or BSA were positive in 33 (91.7%) of these lines (P = 0.0057) (Table 4). This correlation is also reported for the first time in this study. Conversely, in female lines, no significant correlation between the first and the second screening was detected (Supplementary Table S3). This may be because of the influence of the female sex hormone, which affects bone metabolism. The correlation was not observed between “EST profile” and bone X-gal staining in the second screening (P = 0.327; data not shown), which indicates that “EST profile” cannot be used as a substitute for “X-gal” in the first screening. Because EST profiles represent partial sequences originating from a cDNA library of bone and bone marrow, including complementary sequences and areas without an open reading frame, it is possible that the expression patterns of trapped endogenous genes are incompletely monitored. As for “Novel gene”, the functions of genes and non-coding domains cannot be determined using only base sequences. It therefore appears that trapped gene expression in bone or factors that participate in embryogenesis and bone formation, as described in previously reported articles, are necessary based on analysis results of Ayu21-W474, Ayu21-W321, and Ayu21-T167 (Table 1, Fig. 1b, Supplementary Table S1). With regard to “Related article”, although genes encoding molecules associated with bone metabolism showed a tendency to be positive in the second screening (Supplementary Table S1), a greater number of trap lines are needed for more accurate analysis.

The correlation between GT/WT of M.load and Ct.BMC of each line, typical parameters of BSA and BMA, respectively, was also examined in the second screening (Fig. 2). The diagram shows that as bone strength increased, the M.load ratio value exceeded 1.0 and plotted to the right, and as bone strength decreased, it plotted to the left. Furthermore, the ratio value plotted higher as Ct.BMC increased and lower as Ct.BMC decreased. This indicates that as the distance increases between the reference point (where the M.load ratio and Ct.BMC ratio were both 1.0) and the plotted point, there is a high possibility of an abnormal bone phenotype. The correlation between M.load ratio and Ct.BMC ratio was confirmed (Fig. 2, Supplementary Table S4) as expected^15^. There were, however, several outliers such as Ayu21-W456, T167, W361, T2, and W234. Although the Ct. BMC of the outliers showed significant differences compared with the WT, the M.load ratio did not correlate with the Ct.BMC ratio. These findings suggest the presence of biomechanical factors independent of Ct.BMC, such as collagen and other bone matrix proteins. On the other hand, some lines were positive after screening, even though they plotted around the reference point of 1.0 for both the M.load ratio and the Ct.BMC ratio, because abnormal values were observed in parameters other than M.load and Ct.BMC, such as M.work, E.M., Tb.N, or V*m.space (Ayu21-W267, W321, W319, T239, and T57).

Since known genes related to bone metabolism were excluded from the screening, as examples, Ayu21-W268 and Ayu21-T269, which were positive in the second screening (Table 2, Figs 1b, 3 and 4, Supplementary Table S2) and distinct from the reference point in Fig. 2, were analysed to demonstrate the usefulness of this screening system. Ayu21-W268 and Ayu21-T269 lines are trapped *Lbr* and *Nedd4* genes, respectively, and were selected in the first screening based on the results of “EST profile” and “X-gal” (Table 1, Figs 3 and 4). In the second screening of male mice, mice showed weight loss, abnormal ALP values, and other phenotypes that have been reported^16^,^17^,^18^,^19^ in both *Lbr* and *Nedd4* trap lines (Table 2, Fig. 5a,e, Supplementary Table S2). These trapped genes have not been previously reported on bone metabolism. μCT images, BMA, and BSA suggested a loss of trabeculae and a number of abnormal BMA and BSA parameters (Figs 3 and 4). Growth curves and biochemical blood tests revealed clear abnormalities in lines with significantly abnormal bone phenotypes like these lines and, therefore, may be very useful in the screening approach. However, they had no correlation with BMA and BSA, and further examination is needed. After screening, analysis of the *Lbr* trap mouse line confirmed a significant decrease in bone volume for almost all parameters of BMD analysis, cortical bone analysis, and trabecular bone analysis in BMA, and also showed bone fragility in BSA (Fig. 5b,c), as did the second screening (Fig. 3). Expression of the osteoblast differentiation markers *Col1a1, ALP*, and *OCN* were significantly decreased compared with WT mice (Fig. 5d), which suggested involvement of abnormal osteoblast differentiation in the phenotype of the *Lbr* gene trap mouse. Similarly, analysis of the *Nedd4* trap mouse line showed a significant decrease in bone volume in BMA and bone fragility in BSA (Fig. 5e,f). Expression of *Col1a1* and *ALP* mRNA significantly decreased compared with WT mice (Fig. 5g), which also suggested involvement of abnormal osteoblast differentiation in the *Nedd4* gene trap line. These results suggest that such as Ayu21-W203 (*Novel gene*) trap line selected by our screening system as shown in Fig. 4 would also be related to bone metabolism as well as Ayu21-W268 (*Lbr*) and Ayu21-T269 (*Nedd4*). In Fig. 2, Ayu21-W268, Ayu21-T269, and Ayu21-W203 all plotted distinct from the reference point (Fig. 2). Therefore Fig. 2 is useful for the selection of gene trap lines for metabolic bone analyses. Furthermore, this screening approach can be easily applied to more detailed mouse line analyses both *in vivo* and *in vitro*. This novel genetic screening system to identify novel bone metabolism-related genes using the trap mice established by the exchangeable gene trap method is extremely efficient, and the mouse lines found to be abnormal in this screening are highly likely to trap important genes for bone metabolism. These exchangeable gene trap mice are suggested to be very useful as novel bio-resources and are expected to advance various bone metabolism research.

Bassett *et al*. compared each line of gene-knockout mice with normal reference mean data obtained from 77 WT mice^14^. In the current study, we placed emphasis on standardizing the background of the trapped genes among the genotypes in each line and then compared littermate mice in our screening. For this reason, our analysis used three to eight samples (except Ayu21-KBW122/Ayu21-W203) for each genotype, and each parameter was evaluated using [WT mean ± 2.0 SD] as a reference in the second screening (Table 2, Supplementary Table S2). Although 24 trap lines were analysed using Hetero mice in the second screening (Table 2), because a bone phenotype does not always emerge clearly enough with Hetero mice, additional analyses, such as histological examination, detection of gene expression related to bone metabolism, and embryo analysis, might be also necessary in second screening.

This study demonstrates that our screening system (Fig. 1a) is highly efficient, useful, and creative. Discovery of novel bone metabolism-related genes that have not been analysed and their functional analysis using gene trap mice allow for determination of the pathogenesis of metabolic bone diseases, such as osteoporosis, and will contribute greatly to the development of novel therapeutic drugs in the future. This screening system will be applicable to numerous research fields, not only bone metabolism.

## Materials and Methods

### Ethical considerations

All experiments using mice were performed with the approval of the Animal Care and Use Committee and the Genetic Modification Safety Committee of Kumamoto University and the University of Miyazaki, and were in accordance with guidelines for animal experiments and safety management rules for genetic modification of Kumamoto University and the University of Miyazaki.

### Exchangeable gene trap method and Exchangeable Gene Trap Clones database

Gene trapping is a proven method in which reporter genes with no promoter are transfected into ES cells as a trap vector using electroporation. Drug resistance only occurs when the vector is inserted downstream of the promoter of an endogenous gene, and the clone can then be isolated. Araki *et al*. improved on this gene trap method by developing an “exchangeable gene trap method”, which does more than just create a gene-disruption type mutation^9^,^10^,^11^. The vector contains the fusion gene *β-geo*, formed from beta-galactosidase and neomycin-resistance genes. The trapped gene is easily isolated and identified by rapid amplification of 5′ complementary DNA ends (5′ RACE) or the plasmid rescue method. Because expression of the *LacZ* reporter gene is observed time- and tissue-specifically under the control of the trapped gene promoter, the expression pattern *in vivo* can be easily detected with X-gal staining. In addition, by applying the Cre/*lox* site-specific recombination system, the reporter gene can be replaced by any gene (post-insertional modification).

Chimeric mice were produced by aggregation of ES cells with eight-cell embryos of ICR mice (NipponClea) or C57BL/6 females (NipponClea) mated with BDF1 males (NipponClea). Chimeric male mice were mated with C57BL/6 females to obtain F1 Hetero mice. The DNA from F1 Hetero males underwent Southern blotting to confirm that the band pattern matched that of DNA from the original trap ES clones. The clones isolated by the exchangeable gene trap method have been registered and are available in the EGTC database (http://egtc.jp)^12^,^13^. The EGTC database provides information on the trap vector used, the ES cell line, and the 5′ RACE sequence as well as the characteristics of the trapped gene (Table 1). X-gal staining data of systemic organs are available for several lines. In addition, information on the insert position of the trap vector using the University of California Santa Cruz (UCSC) Genome Browser, and information on the trapped genes by linking to National Centre for Biotechnology Information (NCBI) Entrez Gene, Mouse Genome Informatics (MGI, Jackson Lab), Kyoto Encyclopedia of Genes and Genomes (KEGG GENES), International Gene Trap Consortium (IGTC), UniGene, and Expressed Sequence Tag (EST) profile can be easily collected. To date, chimeric mice have been produced from approximately 490 of 1278 ES clones in the EGTC database, which have been established as mouse lines and deposited with the CARD R-BASE (Center for Animal Resources and Development, Resource Database in Kumamoto University)^12^,^13^.

### First screening

In the first screening, all trap clones from the EGTC database were screened using the approaches below (Fig. 1a).

### Determination of tissue specificity by Expressed Sequence Tag profile: “EST profile”

Highly expressing lines on bone and bone marrow (score > 100) in the EST profile from the Unigene database (http://www.ncbi.nlm.nih.gov/unigene) were selected.

### Determination of tissue specificity by X-gal staining: “X-gal”

From the results of X-gal staining performed on EGTC, lines with positively-stained bone and cartilage in trap mice were selected.

### Previously reported articles: “Related article”

A search was conducted for articles related to the trapped genes. Those lines that trapped known genes involved in bone and cartilage metabolism *in vivo* were excluded. Identified lines with trapped genes that are likely to affect bone metabolism, such as angiogenesis-related genes or cell-cell adhesion-related genes, were selected as well as lines with trapped genes that have not been reported *in vitro* or *in vivo* to be associated with bone and cartilage metabolism-related diseases in humans.

### Expressed Sequence Tag/New: “Novel gene”

In the EGTC database, when there was no corresponding gene in the MGI, NCBI, or UCSC databases, but EST (including non-coding RNA) obtained by 5′ RACE existed in UCSC, the trapped genes were classified as “EST”. When there was no corresponding gene and EST, the trapped genes were classified as “New”. Lines selected from “EST” or “New” were considered “Novel genes” and were defined as “not generated and phenotyped before in the literature”.

### Second screening

All lines selected in the first screening were produced by transplanting preserved frozen embryos into a foster parent, and Hetero male mice were mated with Hetero females. All mice were housed in a pathogen-free environment on a 12-h light cycle at 22 ± 2 °C, were fed standard chow, and had free access to water. From the obtained WT and Hetero or Homo mice, between three and eight male and female mice (except Ayu21-KBW122/Ayu21-W203) were selected to map growth curves and for biochemical blood tests. Body weights were measured from 2 to 10 weeks after birth to generate growth curves, and blood samples were collected from 12 to 16 weeks after birth for biochemical blood tests using a chemistry analyser (FUJI DRI-CHEM 4000; Fujifilm Corp., Tokyo, Japan). Levels of sodium (Na, mEq/L), potassium (K, mEq/L), chlorine (Cl, mEq/L), calcium (Ca, mg/dL), inorganic phosphorus (IP, mg/dL), magnesium (Mg, mg/dL), and alkaline phosphatase (ALP, U/L) were measured. After the biochemical blood test, each mouse was euthanized and the femurs were excised. Femurs were stored at −20 °C until experimentation. Homo mice were used for all screenings, but when these were not available because of embryonic lethality, Hetero mice were used and analysed with WT littermate mice. In this study, both male and female mice were tested in the second screening (Supplementary Table S2). The usefulness of the first screening for the discovery of novel genes involved in bone metabolism was investigated using the results of BMA and BSA in the second screening, which directly reflect bone metabolism.

### Micro-computed tomography

For μCT scan preparation, soft tissues surrounding the femur were removed. μCT was performed using a μCT system (ScanXmate-L090H; Comscantecno, Kanagawa, Japan) as described^28^,^29^,^30^.

### Bone morphometric analysis

BMA was performed by creating trabecular bone and cortical bone models for evaluation based on data obtained from μCT scans (TRI/3D-BON; RATOC System Engineering, Tokyo, Japan)^28^,^29^,^30^. The trabecular bone model was cut to a 2.0-mm width proximally from the distal growth plate of the femur, and the cortical bone model was cut to a 0.5-mm width towards the narrowest part of the cortical bone in the middle of the femur. A total of 11 parameters were measured, in accordance with reported articles^31^,^32^,^33^: four for bone mineral density (BMD) analysis (trabecular bone mineral density; Tb.BMD (mg/cm^3^), trabecular bone mineral content; Tb.BMC (mg), cortical bone mineral density; Ct.BMD (mg/cm^3^), cortical bone mineral content; Ct.BMC (mg)); three for cortical bone analysis (cortical volume; Ct.V (mm^3^), cortical thickness; Ct.Th (μm), external line length; Ex.Ln.Le (μm)); and four for trabecular bone analysis (trabecular bone volume; BV (mm^3^), trabecular thickness; Tb.Th (μm), trabecular number; Tb.N (1/mm), marrow space star volume; V*m.space (mm^3^)).

### Biomechanical strength analysis

For BSA, the mouse femur was set at the centre of the experimental bench and a three-point bending test (EZ-test S; Shimadzu Co., Kyoto, Japan) was conducted. The distance between fulcrums was 6 mm, and maximum load (M.load, N), maximum stress (M.stress, N/mm^2^), maximum displacement (M.dsp, mm), maximum work (M.work, J), and elastic modulus (E.M, N/mm2) were measured until the sample broke at a test speed of 1 mm/min. For measurement of M.stress, the cross-sectional area was determined by Ex.Ln.Le (μm) of the cortical bone obtained from BMA, and stress was calculated as an approximate value using the following formula:


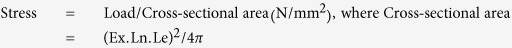


### Bone X-gal staining

Bone X-gal staining was performed using the method of Allen *et al*.^34^. The distal segment of the femur sample was cut in the sagittal plane for observation of trabecular bone. The excised sample was placed in fixative (1% formaldehyde, 0.2% glutaraldehyde, and 0.02% NP-40 in phosphate-buffered saline (PBS)) at room temperature. After 30 min of agitation, the sample was washed with PBS and then transferred into staining solution (5 mM potassium ferricyanide, 5 mM potassium ferrocyanide, 2 mM MgCl_2_, 0.5% X-gal in PBS) and left in staining solution at room temperature overnight. After staining, the sample was washed thoroughly with PBS and fixed in 10% formalin.

### Ayu21-W268 and Ayu21-T269 trap mouse line analyses, B6;CB-*Lbr* ^*Gt(pU-21W)268Card*^ (*Lbr* ^GT^) and B6;CB-*Nedd4* ^*Gt(pU-21T)269Imeg*^(*Nedd4^GT^*)

To verify the usefulness of this screening system, the results of mouse line analyses of Ayu21-W268 (red in Fig. 2) and Ayu21-T269 (orange in Fig. 2) selected in the first and second screening are shown as examples of representative lines, which were distinct from the reference point in Fig. 2. Ayu21-W268 is a Lamin B receptor (*Lbr*) gene trap line and Ayu21-T269 is a neural precursor cell expressed, developmentally down-regulated gene 4 (*Nedd4*) gene trap line.

### Establishment of Ayu21-W268 and Ayu-T269 mouse lines

Ayu21-W268 and Ayu21-T269 clones were isolated using the exchangeable gene trap vectors pU-21W and pU-21T, respectively, and the feeder-free ES cell line KTPU8 derived from the TT2 ES cell line. The TT2 ES cell line is widely used in Japan, and was established from an F1 embryo of C57BL/6 and CBA mice. The produced chimeric male mice were mated with C57BL/6 (Nippon CLEA) females to obtain F1 Hetero mice, and Ayu21-W268 and Ayu21-T269 mouse lines were established^13^.

### Evaluation of bone morphometric analysis, biomechanical strength analysis, and gene expression

BMA and BSA of *Lbr*^*+/+*^ and *Lbr*^*GT/GT*^ mice (n = 5) and *Nedd4*^*+/+*^ and *Nedd4*^*GT/GT*^ mice (n = 5) at 4 weeks old were performed as described above. The excised femur sample was cleared of adherent soft tissue and preserved in RNAlater (Thermo Fisher Scientific, Carlsbad, CA, USA) at 4 °C overnight. The femur was then crushed and dissolved using an RNeasy kit (Qiagen, Valencia, CA, USA) and reverse transcribed into cDNA using Moloney Murine Leukemia Virus Reverse Transcriptase (Thermo Fisher Scientific). Real-time polymerase chain reaction (real-time PCR) (Stepone Plus, Applied Biosystems Co., Carlsbad, CA, USA) was performed on each sample (n = 5) for the expression of the following bone and cartilage metabolism-related genes: bone morphogenetic protein 2 (*BMP2*), *Runx2, ALP, Col1a1, OCN, RANKL, NFATc1*, and *TRAP*. The oligonucleotides used are listed in Supplementary Table S5. Endogenous *β-actin* gene levels were used for normalization and the expression level of each gene was determined using the ΔΔCT method.

### Statistical analysis

For growth curves, biochemical blood tests, BMA, and BSA performed in the second screening, a case was determined as “positive” when at least one parameter was >2.0 SD from the WT mean ([WT mean ± 2.0 SD]), and a case was determined as “negative” when all the parameters were <[WT mean ± 2.0 SD]. After screening, the Student’s t-test was used for all mouse line analyses, and a case was considered significant at P < 0.05. Correlations between biomechanical strength and bone morphometric parameters were evaluated using the Pearson’s correlation test. Correlations between the selected reasons in the first screening and positive lines in the second screening, correlations between the results of the second screening and bone X-gal staining, and correlations between “EST profile” and bone X-gal staining in the second screening were considered positive if the P-value was <0.05 using the Fisher’s one-tailed test.

## Additional Information

**How to cite this article**: Kurogi, S. *et al*. Development of an efficient screening system to identify novel bone metabolism-related genes using the exchangeable gene trap mutagenesis mouse models. *Sci. Rep.* **7**, 40692; doi: 10.1038/srep40692 (2017).

**Publisher's note:** Springer Nature remains neutral with regard to jurisdictional claims in published maps and institutional affiliations.

## Supplementary Material

Supplementary Tables

Excel of Supplementary Table

## Figures and Tables

**Table 1:** List of gene trap lines selected in the first screening.

No.	EGTC ID	Gene Symbol	Chr	Genbank Accession	EGTC X-gal Staining	EST Profile	1st Screening Decision	NPM/NBR
1	18–44	*Bptf*	11	AB212668	ND	388	EST profie	NBR
2	21–103	*New*	4	AB187255	ND	ND	Novel gene	NPM
3	21–106	*New*	5	AB187257	ND	ND	Novel gene	NPM
4	21–129	*EST*	5	AB191465	ND	0	Novel gene	NPM
5	21-B120	*Apobec1*	6	AB247461	ND	153	EST profie	NBR
6	21-B135	*Reep3*	10	AB252053	ND	248	EST profie	NBR
7	21-B186	*Trp53cor1*	17	AB273634	ND	ND	RA^35^	NBR
8	21-B206	*Lpar5*	6	AB264309	ND	29	RA^36^	NBR
9	21-KBT38	*Ankrd11*	8	AB491281	ND	418	EST profie	NBR
10	21-KBW122	*Lima1*	15	AB462912	bone−	410	EST profie	NBR
11	21-KBW180	*Cnn3*	3	AB621934	+	300	X-gal + EST profile	NBR
12	21-KBW205	*Wdr11*	7	AB624508	+	44	X-gal	NBR
13	21-T152	*Tctn1*	5	AB290806	ND	58	RA^37^	NBR
14	21-T167	*2500002B13Rik; lincRNA*	8	AB291807	ND	ND	RA^38^ + Novel gene	NPM
15	21-T188	*EST*	17	AB292813	ND	ND	Novel gene	NPM
16	21-T2	*Msi2*	11	AB285199	ND	80	RA^39^	NBR
17	21-T239	*Wdfy1*	1	AB307034	ND	446	EST profie	NBR
18	21-T269	*Nedd4*	9	AB325690	+	835	X-gal + EST profile	NBR
19	21-T278	*Tmem161a*	8	AB332319	ND	153	EST profie	NBR
20	21-T297	*Mark3*	12	AB330742	ND	190	EST profie	NBR
21	21-T322	*Cyb5b*	8	AB325891	ND	344	EST profie	NBR
22	21-T34	*EST*	2	AB265217	ND	ND	Novel gene	NPM
23	21-T354	*Pkig*	2	AB364965	ND	43	RA^40^	NBR
24	21-T54	*Brd1*	15	AB284376	+	168	X-gal + EST profile	NBR
25	21-T57	*Fbxo17*	7	AB278133	ND	117	EST profie	NBR
26	21-T75	*4933407K13Rik; uncRNA*	X	AB284377	ND	ND	Novel gene	NPM
27	21-W105	*Epb4.1l2*	10	AB458550	ND	498	EST profie	NBR
28	21-W115	*New*	11	AB469153	ND	ND	Novel gene	NPM
29	21-W116	*Cirh1a*	8	AB467276	bone−	344	EST profie	NBR
30	21-W203	*New*	3	AB505799	ND	ND	Novel gene	NPM
31	21-W234	*Etf1*	18	AB504294	+	674	X-gal + EST profile	NBR
32	21-W266	*Ywhag*	5	AB505804	ND	ND	RA^41^	NBR
33	21-W267	*Hdac4*	1	AB505805	ND	65	RA^42^	NBR
34	21-W268	*Lbr*	1	AB510929	+	615	X-gal + EST profile	NBR
35	21-W302	*Umps*	16	AB508947	ND	322	EST profie	NBR
36	21-W319	*Rcl1*	19	AB514155	ND	476	EST profie	NBR
37	21-W321	*1600020E01Rik; lincRNA*	6	AB550825	+	7	X-gal + Novel gene	NPM
38	21-W361	*Dym*	18	AB525777	+	44	X-gal	NBR
39	21-W367	*Ptpn11*	5	AB525781	ND	337	EST profie	NBR
40	21-W373	*Slc38a2*	15	AB525785	+	549	X-gal + EST profile	NBR
41	21-W406	*Ndufaf1*	2	AB517997	ND	153	EST profie	NBR
42	21-W426	*D19Wsu162e*	19	AB533339	ND	520	EST profie	NBR
43	21-W456	*Ppat*	5	AB562513	+	226	X-gal + EST profile	NBR
44	21-W473	*Slc38a4*	15	AB570398	+	7	X-gal	NBR
45	21-W474	*EST*	19	AB665561	+	ND	X-gal + Novel gene	NPM
46	21-W494	*P4hb*	11	AB571318	+	4152	X-gal + EST profile	NBR
47	21-W536	*Osbpl8*	10	AB575992	+	73	X-gal	NBR
48	21-W560	*Rbm12*	2	AB601831	+	1342	X-gal + EST profile	NBR
49	21-W588	*Itpr1*	6	AB576002	+	117	X-gal + EST profile	NBR
50	21-W6	*Ctnnd1*	2	AB427136	ND	513	EST profie	NBR
51	21-W76	*Srr*	11	AB435414	ND	336	EST profie	NBR
52	R	*RhoA*	9	—	ND	754	EST profie	NBR

EGTC: Exchangeable Gene Trap Clones, Chr: Chromosome, ND: not determined, +: positive X-gal staining, bone−: X-gal staining without bone, EST: Expressed Sequence Tag, RA: related articles, Novel gene: EST + New, NPM: novel gene with no prior mutant mice, NBR: known gene with a prior mutant mouse, but no bone phenotype reported, “R” line has not been registered with the EGTC database.

**Table 2:** Second screening results in male trap lines.

No	Gene Symbol	Genotype	Growth Curve	Blood Test	Bone X-gal Staining	Bone Morphometric Analysis																		
						BMD Analysis				Cortical Bone Analysis			Trabecular Bone Analysis				Biomechanical Strength Analysis							
						Tb.BMD	Tb.BMC	Ct.BMD	Ct.BMC	Ct.V	Ct.Th	Ex.Ln.Le	BV	Tb.Th	Tb.N	V*m.space	M.load	M.stress	M.dsp	M.work	E.M.			
1	*Bptf*	WT/Het	↓	+	+			↓	↓	↓		↓									↓			
2	*New*	WT/Hom			+													↑						
3	*New*	WT/Hom				↑			↓	↓		↓	↓	↓	↓	↑								
4	*EST*	WT/Hom	↑	+			↑						↑					↓		↑				
5	*Apobec1*	WT/Hom		+	+			↓								↑		↓						
6	*Reep3*	WT/Hom		+													↑			↑				
7	*Trp53cor1*	WT/Hom	↑	+																				
8	*Lpar5*	WT/Hom	↓	+																				
9	*Ankrd11*	WT/Het		+	+											↑								
10	*Lima1*	WT/Hom	↓	+		↓	↓	↓	↓	↓	↓	↓	↓	↓		↑	↓			↓				
11	*Cnn3*	WT/Het		+	+						↑													
12	*Wdr11*	WT/Hom	↓		+															↓				
13	*Tctn1*	WT/Het			+		↑						↑		↑				↓					
14	*2500002B13 Rik; lincRNA*	WT/Hom		+	+				↑	↑	↑													
15	*EST*	WT/Hom		+																				
16	*Msi2*	WT/Hom	↓	+	+	↓	↓		↓	↓		↓	↓	↓	↓	↑	↓		↓	↓	↓			
17	*Wdfy1*	WT/Hom	↓		+											↑			↑					
18	*Nedd4*	WT/Het	↓	+	+				↓	↓														
19	*Tmem161a*	WT/Hom	↑		+						↑						↑	↑						
20	*Mark3*	WT/Het	↓	+	+						↓					↑								
21	*Cyb5b*	WT/Het		+																				
22	*EST*	WT/Hom	↓	+				↓					↓											
23	*Pkig*	WT/Hom	↑	+	+		↑		↑	↑		↑	↑	↑	↑				↑	↑				
24	*Brd1*	WT/Het	↓	+	+			↓																
25	*Fbxo17*	WT/Hom		+																↑				
26	*4933407K13 Rik; uncRNA*	WT/Het		+																				
27	*Epb4.1l2*	WT/Hom	↓	+	+							↓					↓				↓			
28	*New*	WT/Hom			+																			
29	*Cirh1a*	WT/Het		+																				
30	*New*	WT/Hom	↓	+	+	↑	↑			↑		↑	↑	↑	↑		↑			↑	↑			
31	*Etf1*	WT/Het		+	+			↓			↓								↑		↓			
32	*Ywhag*	WT/Het		+	+																			
33	*Hdac4*	WT/Het	↓													↓								
34	*Lbr*	WT/Hom	↓	+	+	↓	↓		↓	↓		↓		↓		↑	↓	↓			↓			
35	*Umps*	WT/Het			+											↑								
36	*Rcl1*	WT/Het			+										↑									
37	*1600020E01 Rik; lincRNA*	WT/Het		+	+							↑				↑					↑			
38	*Dym*	WT/Hom		+	+				↓	↓								↓			↓			
39	*Ptpn11*	WT/Het	↑	+	+								↑		↑	↓		↓			↑			
40	*Slc38a2*	WT/Hom	↓	+	+						↓										↓			
41	*Ndufaf1*	WT/Het	↑	+				↓			↓								↓	↓				
42	*D19Wsu162e*	WT/Hom	↓	+	+																			
43	*Ppat*	WT/Het	↓	+	+			↑																
44	*Slc38a4*	WT/Het	↓	+	+														↓	↓				
45	*EST*	WT/Het	↓	+	+							↓		↑				↑						
46	*P4hb*	WT/Hom	↓	+	+		↓		↓	↓		↓	↓	↓	↓	↑	↓							
47	*Osbpl8*	WT/Hom	↓		+		↑				↓		↑	↑	↑		↓			↓				
48	*Rbm12*	WT/Hom		+	+								↓											
49	*Itpr1*	WT/Het	↓		+						↓			↓										
50	*Ctnnd1*	WT/Het	↓	+	+														↑					
51	*Srr*	WT/Hom	↓													↑		↓			↓			
52	*RhoA*	WT/Het																						

WT: wild-type, Het: Heterozygote, Hom: Homozygote, Tb.BMD: trabecular bone mineral density, Tb.BMC: trabecular bone mineral content, Ct.BMD: cortical bone mineral density, Ct.BMC: cortical bone mineral content, Ct.V: cortical bone volume, Ct.Th: cortical bone thickness, Ex.Ln.Le: external line length, BV: trabecular bone volume, Tb.Th: trabecular bone thickness, Tb.N: trabecular bone number, V*m.space: marrow space star volume, M.load: maximum load, M.stress: maximum stress, M.dsp: maximum displacement, M.work: maximum work, E.M: elastic modulus. +: abnormal blood test results, positive X-gal staining, ↑: > [WT mean + 2.0 SD], ↓: < [WT mean − 2.0 SD], “R” line has not been registered with the EGTC database.

**Table 3:** Correlation between the first screening items and the second screening results (BMA or BSA) in male trap lines.

	1^st^ screening selected lines	2^nd^ screening positive lines	BSA positive lines	BMA positive lines	2^nd^ screening negative lines	P value
**EST profile**	30	26	16 (53.3)	23 (76.7)	4	0.1828
**X-gal**	16	16	10 (62.5)	14 (87.5)	0	0.0161
**RA**	8	5	3 (37.5)	5 (62.5)	3	0.1712
**Novel gene**	11	8	5 (45.5)	7 (63.6)	3	0.3533

Biomechanical strength analysis (BSA) and bone morphometric analysis (BMA) indicate the number of positive lines (%). One-tailed Fisher’s exact test was calculated for lines with a difference observed for each selected item in the first and second screening (BMA or BSA). EST: Expressed Sequence Tag, RA: related article, Novel gene: EST + New.

**Table 4:** Correlation between the second screening results (BMA or BSA) and bone X-gal staining in the second screening in male trap lines.

	X-gal staining		Total	P-value
	(+)	(−)		
**2**^**nd**^ **screening positive lines**	33 (78.6)	9 (21.4)	42	0.0057
**2**^**nd**^ **screening negative lines**	3 (30.0)	7 (70.0)	10	

One-tailed Fisher’s exact test was used to confirm the correlation between the second screening results (BMA or BSA) and bone X-gal staining in the second screening. BMA: bone morphometric analysis, BSA: biomechanical strength analysis.
