# Supplementary material for: Development of an efficient screening system to identify novel bone metabolism-related genes using the exchangeable gene trap mutagenesis mouse models
Source: Sci Rep. 2017 Jan 20;7:40692. doi: 10.1038/srep40692 (PMC5247747; doi:10.1038/srep40692)
Supplement: Supplementary Tables [file srep40692-s1.pdf]

**Development of an efficient screening system to identify novel bone metabolism-related genes using the exchangeable gene trap mutagenesis mouse models**

Syuji Kurogi, MD[1,a], Tomohisa Sekimoto, MD, PhD[1,a], Taro Funamoto, MD, PhD[1], Tomomi Ohta, MD[1], Shihoko Nakamura, MD[1], Takuya Nagai, MD[1], Mai Nakahara, PhD[2], Kumiko Yoshinobu, MSc[2], Kimi Araki, PhD[2], Masatake Araki, PhD[2], & Etsuo Chosa, MD, PhD[1,*]

[1] Division of Orthopaedic Surgery, Department of Medicine of Sensory and Motor Organs, Faculty of Medicine, University of Miyazaki, Japan

[2] Institute of Resource Development and Analysis, Kumamoto University, Japan

[a] S.K. and T.S. made equal contributions to this paper.

**Supplementary Table S1. Selected genes screened by previous reports in the first screening**

| EGTC ID | Gene Symbol | Reported gene function | Related article |
|---|---|---|---|
| 21-B186 | *Trp53cor1* | Dephosphorylation of the nuclear factor of activated T cells (NFAT) transcription factor | [35] |
| 21-B206 | *Lpar5* | Inhibitor of B cell antigen receptor signaling and antibody response | [36] |
| 21-T152 | *Tctn1* | Regulator of mouse Hedgehog signaling | [37] |
| 21-T167 | *2500002B13Rik* | Participation of embryogenesis | [38] |
| 21-T2 | *Msi2* | Control of TGF-β signaling | [39] |
| 21-T354 | *Pkig* | Participation of BMP-2-induced osteoblastic differentiation | [40] |
| 21-W266 | *Ywhag* | Participation of desmosome formation and cell-cell adhesion in vitro | [41] |
| 21-W267 | *Hdac4* | Control of chondrocyte hypertrophy during skeletogenesis | [42] |

EGTC: Exchangeable Gene Trap Clones, *Trp53cor1*: tumor protein p53 pathway corepressor 1, *Lpar5*: lysophosphatidic acid receptor 5, *Tctn1*: tectonic family member 1, *Msi2*: Musashi homolog 2, *Pkig*: protein kinase inhibitor, gamma, *Ywhag*: tyrosine 3-monooxygenase/tryptophan 5-monooxygenase activation protein, gamma polypeptide, *Hdac4*: histone deacetylase 4.

# Supplementary table S2. Second screening results

| No. | EGTC ID / CARD ID | Gene symbol / Chr / Strain Name | Cell Line / Trap Vector | | Group | Sample Number | Week old | Na [mEq/L] | K [mEq/L] | Cl [mEq/L] | Ca [mg/dL] | IP [mg/dL] | Mg [mg/dL] | ALP [U/L] | 2W [g] | 4W [g] | 6W [g] | 8W [g] | 10W [g] | Tb.BMD [mg/cm3] | Tb.BMC [mg] | Ct.BMD [mg/cm3] | Ct.BMC [mg] | Ct.V [mm3] | Ct.Th [μm] | Ex.Ln.Le [μm] | BV [mm3] | Tb.Th [μm] | Tb.N [1/mm] | V*m.space [mm3] | M. load [N] | M. stress [N/mm2] | M. dsp [mm] | M. work [J] | E.M. [N/mm2] | 2nd screening Bone X-gal staining |
|---|---|---|---|---|---|---|---|---|---|---|---|---|---|---|---|---|---|---|---|---|---|---|---|---|---|---|---|---|---|---|---|---|---|---|---|---|
| | | | | | | | | Biochemical blood test | | | | | | | Growth curve | | | | | Bone morphometric analysis: BMD analysis | | | | Cortical bone analysis | | | Trabecular bone analysis | | | | Biomechanical strength analysis | | | | | |
| 1 | 18-44 | *Bptf* | *KTPU10* | 【Mean】 | M WT | 3 | 12-16 | 148.00 | 4.30 | 109.33 | 9.70 | 4.90 | 2.25 | 301.33 | ND | 12.68 | 24.50 | 25.43 | 29.49 | 504.83 | 0.7360 | 1400.10 | 0.9810 | 0.7001 | 265.71 | 5697.38 | 1.4836 | 98.7686 | 2.0020 | 0.0546 | 24.300 | 10.687 | 0.8312 | 0.00499 | 31716 | + |
| | 609 | 11 | *pU-18* | | M Hetero | 4 | 10-12 | 150.25 | 4.68 | 111.50 | 10.08 | 5.80 | 2.55 | 485.50 | ND | 13.24 | 20.25 | 21.62 | 25.25 | 450.90 | 0.4958 | 1327.83 | 0.7415 | 0.5571 | 238.29 | 5021.60 | 1.0921 | 92.2889 | 1.8947 | 0.0634 | 18.848 | 8.587 | 0.9434 | 0.00401 | 23608 | |
| | | *B6;CB-BptfGt(pU-18)44Imeg* | | | F WT | 4 | 13-24 | 149.25 | 3.95 | 111.00 | 10.13 | 4.68 | 2.33 | 486.00 | ND | 13.55 | 18.81 | 19.96 | 22.18 | 365.75 | 0.2085 | 1392.38 | 0.7785 | 0.5583 | 250.25 | 4872.60 | 0.5524 | 86.6663 | 1.5528 | 0.0842 | 17.466 | 9.705 | 0.5730 | 0.00314 | 27845 | |
| | | | | | F Hetero | 4 | 11-24 | 149.75 | 3.68 | 114.00 | 10.13 | 5.30 | 2.38 | 473.75 | ND | 10.21 | 17.05 | 16.83 | 18.65 | 422.18 | 0.3008 | 1351.00 | 0.7530 | 0.5559 | 255.22 | 4828.03 | 0.7214 | 98.5164 | 1.6368 | 0.0547 | 16.659 | 8.827 | 0.4342 | 0.00256 | 19807 | |
| | | | | 【SD】 | M WT | | | 4.36 | 0.42 | 2.08 | 0.00 | 0.28 | 0.35 | 38.53 | ND | 2.28 | 1.46 | 0.26 | 0.16 | 37.36 | 0.2343 | 31.66 | 0.0894 | 0.0504 | 22.09 | 170.88 | 0.4004 | 9.6729 | 0.4825 | 0.0383 | 3.416 | 3.880 | 0.2129 | 0.00129 | 1534 | |
| | | | | | M Hetero | | | 1.89 | 0.61 | 1.73 | 0.22 | 0.41 | 0.37 | 177.69 | ND | 0.95 | ND | 1.51 | 0.00 | 51.74 | 0.2431 | 31.25 | 0.1146 | 0.0784 | 17.53 | 385.83 | 0.4697 | 13.3007 | 0.1235 | 0.0066 | 0.298 | 0.790 | 0.3394 | 0.00082 | 1938 | |
| | | | | | F WT | | | 0.50 | 0.40 | 1.83 | 0.21 | 0.63 | 0.30 | 104.32 | ND | 1.75 | 0.66 | 0.41 | 1.19 | 50.91 | 0.1086 | 42.29 | 0.0936 | 0.0516 | 24.42 | 138.14 | 0.2152 | 12.4876 | 0.3334 | 0.0500 | 2.418 | 1.965 | 0.1475 | 0.00036 | 2555 | |
| | | | | | F Hetero | | | 0.96 | 0.30 | 1.41 | 0.17 | 0.42 | 0.35 | 125.76 | ND | 2.35 | 0.05 | 1.01 | 0.48 | 49.58 | 0.0418 | 49.07 | 0.1031 | 0.0559 | 37.22 | 109.91 | 0.0963 | 16.0311 | 0.2855 | 0.0103 | 3.906 | 1.762 | 0.0658 | 0.00056 | 8811 | |
| 2 | 21-103 | *New* | *KTPU10* | 【Mean】 | M WT | 3 | 14-15 | 150.33 | 3.87 | 111.00 | 8.73 | 7.57 | 2.57 | 318.00 | 7.73 | 15.62 | 24.17 | 27.40 | 29.17 | 463.60 | 0.5766 | 1170.37 | 0.6376 | 0.5445 | 219.68 | 5419.75 | 1.2503 | 71.3480 | 2.2784 | 0.0630 | 20.768 | 8.864 | 1.1018 | 0.00566 | 4799 | + |
| | 458 | 4 | *pU-21* | | M Homo | 3 | 14 | 150.33 | 4.47 | 110.00 | 9.20 | 8.87 | 2.73 | 492.33 | 7.92 | 14.01 | 22.89 | 26.82 | 29.24 | 482.30 | 0.7047 | 1188.10 | 0.6755 | 0.5686 | 237.12 | 5338.43 | 1.4916 | 77.2527 | 2.6747 | 0.0235 | 22.503 | 9.928 | 1.2974 | 0.00711 | 3218 | |
| | | B6;CB-*Gt(pU-21)103Imeg* | | | F WT | 2 | 14-16 | 150.50 | 3.95 | 110.00 | 9.20 | 7.85 | 2.30 | 523.50 | 6.81 | 11.46 | 18.74 | 21.39 | 22.78 | 393.45 | 0.1396 | 1189.25 | 0.5009 | 0.4199 | 214.80 | 4420.21 | 0.3527 | 59.5835 | 1.2605 | 0.1753 | 13.018 | 8.379 | 0.8578 | 0.00294 | 4309 | |
| | | | | | F Homo | 3 | 14 | 151.33 | 4.10 | 115.00 | 9.17 | 8.87 | 2.50 | 693.67 | 6.64 | 12.42 | 19.10 | 21.43 | 22.82 | 428.43 | 0.2017 | 1213.03 | 0.6020 | 0.4963 | 230.99 | 4831.75 | 0.4708 | 63.7459 | 1.5941 | 0.0906 | 20.080 | 10.880 | 1.1285 | 0.00553 | 5477 | |
| | | | | 【SD】 | M WT | | | 2.31 | 0.47 | 2.65 | 0.50 | 1.02 | 0.25 | 20.81 | 2.00 | 3.11 | 1.66 | 1.76 | 1.73 | 17.31 | 0.2130 | 51.14 | 0.0536 | 0.0317 | 9.09 | 167.17 | 0.4372 | 5.1262 | 0.4825 | 0.0476 | 2.158 | 0.406 | 0.2442 | 0.00263 | 2274 | |
| | | | | | M Homo | | | 0.58 | 0.12 | 3.61 | 0.10 | 0.25 | 0.06 | 44.11 | 2.20 | 1.12 | 1.79 | 0.80 | 2.04 | 16.82 | 0.1098 | 15.74 | 0.0029 | 0.0087 | 1.40 | 66.01 | 0.2365 | 0.9485 | 0.3297 | 0.0108 | 1.189 | 0.633 | 0.1991 | 0.00081 | 872 | |
| | | | | | F WT | | | 0.71 | 0.07 | 1.41 | 0.00 | 1.34 | 0.14 | 67.18 | 1.58 | 5.10 | 2.18 | 1.90 | 1.39 | 2.62 | 0.0751 | 67.53 | 0.0798 | 0.0433 | 32.57 | 32.28 | 0.1905 | 0.8669 | 0.5556 | 0.1024 | 1.319 | 0.971 | 0.0666 | 0.00015 | 1100 | |
| | | | | | F Homo | | | 0.58 | 0.60 | 1.00 | 0.31 | 1.46 | 0.10 | 15.57 | 0.88 | 1.61 | 1.17 | 1.23 | 0.98 | 13.35 | 0.0227 | 10.45 | 0.0168 | 0.0118 | 3.50 | 127.80 | 0.0419 | 1.2391 | 0.1943 | 0.0159 | 2.897 | 2.191 | 0.1938 | 0.00080 | 2403 | |
| 3 | 21-106 | *New* | *KTPU10* | 【Mean】 | M WT | 3 | 14-15 | 149.67 | 4.03 | 111.67 | 9.73 | 5.93 | 2.33 | 259.67 | 6.76 | 14.16 | 22.46 | 25.42 | 28.70 | 493.83 | 0.4060 | 1169.80 | 0.6513 | 0.5566 | 232.88 | 5316.04 | 0.8344 | 72.2988 | 1.8437 | 0.1063 | 22.642 | 10.154 | 0.7419 | 0.00487 | 13923 | − |
| | 631 | 5 | *pU-21* | | M Homo | 3 | 14 | 150.67 | 4.50 | 113.33 | 9.73 | 5.97 | 2.03 | 290.67 | 7.60 | 15.98 | 21.54 | 22.84 | 25.03 | 576.23 | 0.1974 | 1176.63 | 0.5410 | 0.4590 | 223.49 | 4638.15 | 0.3786 | 63.5351 | 1.1936 | 0.2286 | 19.713 | 11.507 | 0.6523 | 0.00369 | 13953 | |
| | | B6;CB-*Gt(pU-21)106Imeg* | | | F WT | 3 | 14-15 | 149.00 | 3.87 | 113.67 | 9.27 | 5.97 | 2.10 | 394.00 | 6.35 | 12.91 | 18.48 | 20.30 | 21.69 | 432.37 | 0.0739 | 1199.23 | 0.5477 | 0.4567 | 225.75 | 4594.25 | 0.1640 | 58.6990 | 0.6394 | 0.3452 | 18.858 | 11.386 | 0.5780 | 0.00335 | 12805 | |
| | | | | | F Homo | 3 | 14-15 | 150.00 | 4.37 | 114.67 | 9.33 | 6.83 | 2.30 | 388.00 | 6.42 | 14.80 | 19.03 | 20.13 | 21.88 | 399.63 | 0.0342 | 1173.47 | 0.4984 | 0.4237 | 208.81 | 4515.26 | 0.0834 | 58.6289 | 0.3657 | 0.5294 | 15.057 | 9.268 | 0.8108 | 0.00318 | 7959 | |
| | | | | 【SD】 | M WT | | | 0.58 | 0.32 | 1.15 | 0.12 | 0.59 | 0.15 | 26.63 | 2.41 | 4.67 | 2.77 | 2.48 | 3.11 | 18.89 | 0.1058 | 48.36 | 0.0362 | 0.0134 | 18.00 | 182.52 | 0.2134 | 3.9472 | 0.3247 | 0.0407 | 3.517 | 2.243 | 0.0781 | 0.00137 | 2132 | |
| | | | | | M Homo | | | 0.58 | 0.30 | 1.15 | 0.31 | 1.06 | 0.25 | 16.92 | 0.36 | 1.04 | 2.19 | 2.59 | 3.15 | 158.66 | 0.0752 | 31.17 | 0.0702 | 0.0475 | 24.32 | 183.94 | 0.2213 | 15.7086 | 0.3181 | 0.1093 | 2.263 | 1.019 | 0.0204 | 0.00042 | 1476 | |
| | | | | | F WT | | | 1.00 | 0.32 | 1.15 | 0.15 | 1.88 | 0.17 | 112.80 | 1.60 | 0.27 | 0.38 | 0.19 | 0.56 | 60.98 | 0.0545 | 18.92 | 0.0395 | 0.0317 | 15.53 | 297.39 | 0.1133 | 7.0813 | 0.3167 | 0.1445 | 1.829 | 2.412 | 0.2260 | 0.00046 | 2288 | |
| | | | | | F Homo | | | 2.00 | 0.15 | 1.53 | 0.32 | 0.60 | 0.20 | 38.59 | 0.25 | 0.33 | 1.60 | 1.21 | 0.61 | 29.38 | 0.0199 | 31.45 | 0.0926 | 0.0700 | 21.51 | 373.12 | 0.0507 | 5.0135 | 0.2108 | 0.1729 | 2.209 | 0.632 | 0.1750 | 0.00077 | 1584 | |
| 4 | 21-129 | *EST* | *KTPU10* | 【Mean】 | M WT | 3 | 14 | 149.67 | 3.80 | 112.00 | 9.70 | 4.67 | 2.50 | 283.33 | 6.17 | 11.98 | 19.37 | 21.20 | 22.76 | 433.53 | 0.1777 | 1217.13 | 0.5382 | 0.4425 | 234.85 | 4364.84 | 0.4090 | 64.2027 | 1.5355 | 0.1073 | 14.852 | 9.774 | 0.6279 | 0.00247 | 9750 | − |
| | 526 | 5 | *pU-21* | | M Homo | 3 | 14-16 | 150.33 | 3.70 | 113.67 | 9.40 | 4.23 | 2.30 | 241.00 | 5.98 | 12.90 | 21.76 | 24.96 | 27.01 | 475.17 | 0.3938 | 1187.60 | 0.6181 | 0.5199 | 229.31 | 5061.56 | 0.8249 | 71.5151 | 1.9521 | 0.0871 | 18.512 | 9.075 | 0.4567 | 0.00330 | 11635 | |
| | | B6;CB-*Gt(pU-21)129Imeg* | | | F WT | 3 | 14-15 | 149.67 | 4.33 | 115.00 | 9.50 | 6.03 | 2.37 | 375.00 | 6.56 | 13.66 | 18.33 | 19.50 | 21.09 | 393.63 | 0.0938 | 1206.93 | 0.5340 | 0.4424 | 227.47 | 4431.00 | 0.2359 | 57.9661 | 1.0738 | 0.1700 | 16.592 | 10.585 | 0.4346 | 0.00251 | 11548 | |
| | | | | | F Homo | 3 | 14-15 | 150.00 | 3.70 | 114.67 | 9.60 | 5.86 | 2.43 | 385.67 | 5.42 | 11.77 | 17.88 | 19.64 | 21.07 | 412.20 | 0.1043 | 1204.67 | 0.5451 | 0.4525 | 223.21 | 4586.49 | 0.2481 | 59.0860 | 0.9856 | 0.2135 | 15.202 | 9.058 | 0.4503 | 0.00236 | 11027 | |
| | | | | 【SD】 | M WT | | | 1.15 | 0.17 | 2.00 | 0.20 | 0.72 | 0.26 | 18.01 | 0.30 | 2.50 | 1.02 | 0.85 | 0.60 | 55.26 | 0.0382 | 13.82 | 0.0730 | 0.0628 | 12.86 | 368.60 | 0.0625 | 7.2276 | 0.3089 | 0.0486 | 2.153 | 0.226 | 0.1290 | 0.00026 | 2771 | |
| | | | | | M Homo | | | 2.52 | 0.69 | 2.08 | 0.35 | 0.06 | 0.17 | 41.04 | 1.01 | 2.67 | 1.54 | 2.14 | 2.32 | 57.67 | 0.1480 | 33.20 | 0.0896 | 0.0673 | 16.83 | 400.98 | 0.2231 | 7.7371 | 0.2962 | 0.0579 | 3.327 | 1.177 | 0.1127 | 0.00053 | 2150 | |
| | | | | | F WT | | | 1.53 | 0.45 | 1.00 | 0.10 | 1.57 | 0.06 | 49.52 | 1.06 | 1.77 | 1.77 | 1.83 | 1.91 | 8.30 | 0.0217 | 5.25 | 0.0463 | 0.0378 | 8.49 | 267.81 | 0.0498 | 0.1327 | 0.0948 | 0.0105 | 2.208 | 0.326 | 0.0578 | 0.00061 | 988 | |
| | | | | | F Homo | | | 2.00 | 0.85 | 2.89 | 0.20 | 2.38 | 0.25 | 77.36 | 1.31 | 2.52 | 1.39 | 1.51 | 1.58 | 28.38 | 0.0356 | 7.22 | 0.0143 | 0.0112 | 6.59 | 105.52 | 0.0733 | 4.5214 | 0.1535 | 0.0581 | 2.493 | 1.186 | 0.0747 | 0.00066 | 2372 | |
| 5 | 21-B120 | *Apobec1* | *KTPU8* | 【Mean】 | M WT | 4 | 14 | 150.25 | 3.77 | 113.00 | 9.50 | 7.30 | 2.45 | 350.00 | 5.45 | 12.68 | 21.86 | 25.51 | 27.67 | 495.93 | 0.6585 | 1210.03 | 0.6520 | 0.5387 | 228.46 | 5220.94 | 1.3376 | 74.0066 | 2.3886 | 0.0406 | 21.098 | 9.714 | 0.5070 | 0.00460 | 15432 | + |
| | 690 | 6 | *pU-21B* | | M Homo | 4 | 14 | 150.75 | 4.40 | 113.75 | 9.78 | 7.15 | 2.43 | 332.75 | 5.35 | 11.13 | 21.40 | 26.40 | 28.24 | 508.13 | 0.5945 | 1189.83 | 0.7168 | 0.6027 | 237.77 | 5634.93 | 1.1864 | 75.8970 | 2.0159 | 0.0878 | 21.701 | 8.654 | 0.4782 | 0.00469 | 14439 | |
| | | B6;CB-*Apobec1Gt(pU-21B)120Imeg* | | | F WT | 3 | 14 | 149.00 | 3.73 | 111.33 | 9.57 | 5.00 | 2.53 | 350.33 | 5.34 | 12.18 | 17.37 | 19.09 | 21.77 | 390.05 | 0.1244 | 1201.25 | 0.5035 | 0.4188 | 206.15 | 4521.46 | 0.3138 | 57.1253 | 1.2125 | 0.1721 | 13.633 | 8.377 | 0.3963 | 0.00213 | 9093 | |
| | | | | | F Homo | 4 | 14-16 | 149.25 | 3.68 | 115.00 | 9.43 | 7.45 | 2.35 | 423.50 | 6.74 | 13.25 | 20.44 | 21.72 | 21.97 | 418.03 | 0.1585 | 1192.78 | 0.5493 | 0.4604 | 204.33 | 4928.37 | 0.3743 | 59.9266 | 1.1457 | 0.1853 | 13.730 | 7.092 | 0.3793 | 0.00211 | 9515 | |
| | | | | 【SD】 | M WT | | | 2.22 | 0.31 | 2.16 | 0.36 | 1.27 | 0.30 | 83.81 | 1.22 | 3.58 | 2.69 | 2.18 | 2.79 | 42.40 | 0.2662 | 7.07 | 0.0706 | 0.0581 | 13.72 | 310.11 | 0.4978 | 7.3096 | 0.3602 | 0.0222 | 2.360 | 0.443 | 0.2372 | 0.00105 | 1313 | |
| | | | | | M Homo | | | 1.71 | 0.84 | 1.26 | 0.10 | 1.81 | 0.38 | 57.89 | 1.96 | 4.22 | 3.34 | 2.14 | 2.38 | 24.14 | 0.1910 | 15.37 | 0.0494 | 0.0447 | 12.42 | 299.90 | 0.3474 | 3.8253 | 0.3464 | 0.0575 | 0.842 | 1.093 | 0.0859 | 0.00141 | 1318 | |
| | | | | | F WT | | | 1.00 | 0.15 | 0.58 | 0.21 | 0.79 | 0.15 | 42.06 | 0.91 | 2.59 | 0.15 | 0.56 | 0.86 | 14.07 | 0.0377 | 5.44 | 0.0177 | 0.0166 | 4.36 | 109.74 | 0.0862 | 3.8270 | 0.0728 | 0.0093 | 0.704 | 0.026 | 0.0088 | 0.00016 | 2348 | |
| | | | | | F Homo | | | 0.96 | 0.13 | 2.16 | 0.34 | 1.42 | 0.24 | 52.44 | 1.30 | 3.07 | 1.69 | 2.29 | 1.25 | 21.27 | 0.0574 | 15.98 | 0.0359 | 0.0270 | 6.45 | 157.90 | 0.1285 | 3.3518 | 0.2040 | 0.0448 | 1.690 | 0.637 | 0.0651 | 0.00039 | 3110 | |
| 6 | 21-B135 | *Reep3* | *KTPU8* | 【Mean】 | M WT | 3 | 14 | 150.33 | 3.90 | 113.00 | 9.73 | 6.13 | 2.30 | 293.33 | 5.95 | 13.44 | 21.72 | 23.62 | 25.80 | 487.87 | 0.3999 | 1201.07 | 0.6171 | 0.5133 | 232.48 | 4955.71 | 0.8294 | 74.5107 | 2.0747 | 0.0557 | 19.257 | 9.933 | 0.4787 | 0.00377 | 14485 | − |
| | 621 | 10 | *pU-21B* | | M Homo | 3 | 14-16 | 150.33 | 4.07 | 114.67 | 9.70 | 4.87 | 2.17 | 324.67 | 7.06 | 16.07 | 22.03 | 23.31 | 24.62 | 487.13 | 0.5139 | 1218.60 | 0.6654 | 0.5446 | 238.13 | 5103.17 | 1.0469 | 73.7328 | 1.9777 | 0.0603 | 24.163 | 11.592 | 0.3508 | 0.00463 | 16514 | |
| | | B6;CB-*Reep3Gt(pU-21B)135Imag* | | | F WT | 3 | 14-15 | 149.33 | 3.20 | 111.67 | 9.40 | 4.90 | 2.37 | 375.00 | 6.54 | 14.39 | 18.84 | 19.69 | 20.83 | 380.07 | 0.0812 | 1193.20 | 0.5167 | 0.4331 | 211.37 | 4563.33 | 0.2090 | 54.3739 | 0.8900 | 0.2509 | 16.200 | 9.810 | 0.3503 | 0.00257 | 12903 | |
| | | | | | F Homo | 2 | 15-16 | 151.33 | 3.57 | 117.67 | 9.40 | 5.47 | 2.33 | 392.00 | 7.83 | 14.96 | 19.25 | 20.36 | 20.84 | 399.70 | 0.0850 | 1230.20 | 0.5472 | 0.4447 | 216.85 | 4591.96 | 0.2071 | 57.1076 | 0.8639 | 0.2338 | 19.103 | 11.462 | 0.3236 | 0.00314 | 15542 | |
| | | | | 【SD】 | M WT | | | 1.53 | 0.30 | 2.65 | 0.25 | 1.12 | 0.26 | 15.28 | 0.87 | 1.45 | 1.25 | 0.87 | 1.29 | 29.54 | 0.1151 | 27.51 | 0.0712 | 0.0504 | 19.15 | 222.83 | 0.1913 | 2.9829 | 0.3487 | 0.0222 | 1.833 | 1.741 | 0.0782 | 0.00042 | 2159 | |
| | | | | | M Homo | | | 1.15 | 0.76 | 2.08 | 0.26 | 0.61 | 0.21 | 121.36 | 1.28 | 3.24 | 1.56 | 1.18 | 1.25 | 32.50 | 0.3508 | 23.94 | 0.1680 | 0.1278 | 24.31 | 674.86 | 0.6496 | 5.0597 | 0.2937 | 0.0167 | 5.769 | 0.566 | 0.0169 | 0.00196 | 824 | |
| | | | | | F WT | | | 0.58 | 0.46 | 3.06 | 0.26 | 0.75 | 0.31 | 79.30 | 0.64 | 1.82 | 1.42 | 1.69 | 2.16 | 1.44 | 0.0370 | 14.67 | 0.0054 | 0.0084 | 2.97 | 105.69 | 0.0968 | 3.1151 | 0.2596 | 0.0837 | 1.343 | 1.226 | 0.0918 | 0.00023 | 1632 | |
| | | | | | F Homo | | | 0.58 | 0.12 | 2.31 | 0.20 | 0.72 | 0.12 | 8.54 | 1.57 | 2.62 | 1.99 | 2.15 | 1.30 | 18.96 | 0.0197 | 26.05 | 0.0253 | 0.0113 | 13.64 | 110.74 | 0.0413 | 3.0772 | 0.0383 | 0.0100 | 3.774 | 2.809 | 0.0305 | 0.00057 | 2834 | |
| 7 | 21-B186 | *Trp53cor1* | *KTPU8* | 【Mean】 | M WT | 5 | 14-15 | 149.67 | 4.13 | 112.00 | 9.33 | 7.43 | 2.70 | 399.67 | 6.99 | 15.75 | 23.41 | 25.57 | 27.94 | 448.34 | 0.4261 | 1156.00 | 0.5853 | 0.5060 | 219.13 | 5086.89 | 0.9531 | 67.8157 | 2.2571 | 0.0442 | 20.439 | 9.857 | 0.4418 | 0.00347 | 16645 | − |
| | 726 | 17 | *pU-21B* | | M Homo | 5 | 14-15 | 150.00 | 4.60 | 112.67 | 9.27 | 7.77 | 2.53 | 367.33 | 7.77 | 17.73 | 24.40 | 26.83 | 28.87 | 442.26 | 0.4530 | 1154.38 | 0.5990 | 0.5180 | 213.15 | 5273.82 | 1.0093 | 66.3178 | 2.1613 | 0.0573 | 19.390 | 8.713 | 0.4423 | 0.00390 | 15515 | |
| | | B6;CB-*Trp53cor1Gt(pU-21B)186Imeg* | | | F WT | 3 | 15 | 149.33 | 3.57 | 113.33 | 8.93 | 6.70 | 2.47 | 494.00 | 6.87 | 13.80 | 18.77 | 19.89 | 21.87 | 361.17 | 0.0549 | 1163.20 | 0.4861 | 0.4179 | 203.66 | 4527.05 | 0.1434 | 51.1882 | 0.7024 | 0.3247 | 16.463 | 10.108 | 0.3701 | 0.00283 | 14175 | |
| | | | | | F Homo | 3 | 15 | 149.67 | 4.20 | 114.00 | 9.10 | 6.77 | 2.13 | 469.00 | 6.47 | 14.74 | 18.61 | 19.64 | 20.96 | 400.63 | 0.0983 | 1161.93 | 0.5103 | 0.4391 | 204.08 | 4727.14 | 0.2383 | 55.3507 | 0.8398 | 0.2983 | 17.618 | 9.913 | 0.4506 | 0.00310 | 10489 | |
| | | | | 【SD】 | M WT | | | 1.53 | 0.12 | 0.00 | 0.06 | 0.60 | 0.10 | 44.74 | 0.68 | 0.09 | 0.35 | 1.01 | 0.76 | 29.03 | 0.1227 | 15.30 | 0.0622 | 0.0495 | 15.29 | 188.21 | 0.2124 | 6.1543 | 0.2111 | 0.0105 | 4.634 | 1.575 | 0.1048 | 0.00100 | 2639 | |
| | | | | | M Homo | | | 1.00 | 0.17 | 2.31 | 0.06 | 0.95 | 0.06 | 77.03 | 0.55 | 0.25 | 0.43 | 1.21 | 0.97 | 41.89 | 0.2466 | 19.10 | 0.0911 | 0.0712 | 16.13 | 353.37 | 0.4857 | 5.1667 | 0.4333 | 0.0258 | 2.995 | 0.190 | 0.0785 | 0.00116 | 1597 | |
| | | | | | F WT | | | 2.08 | 0.32 | 2.08 | 0.31 | 0.62 | 0.06 | 49.33 | 0.70 | 0.80 | 0.39 | 1.07 | 1.61 | 22.73 | 0.0244 | 2.96 | 0.0158 | 0.0126 | 2.47 | 134.63 | 0.0590 | 3.7318 | 0.2166 | 0.0853 | 0.247 | 0.486 | 0.0535 | 0.00056 | 308 | |
| | | | | | F Homo | | | 0.58 | 0.35 | 1.00 | 0.17 | 0.21 | 0.06 | 55.11 | 0.64 | 0.87 | 0.60 | 1.07 | 1.52 | 26.60 | 0.0662 | 15.45 | 0.0230 | 0.0144 | 7.05 | 34.01 | 0.1600 | 2.8634 | 0.4773 | 0.1781 | 2.638 | 1.516 | 0.0529 | 0.00034 | 5333 | |
| 8 | 21-B206 | *Lpar5* | *KTPU8* | 【Mean】 | M WT | 3 | 15-16 | 150.67 | 4.07 | 114.67 | 9.67 | 5.67 | 2.33 | 327.33 | 7.84 | 17.69 | 22.54 | 25.33 | 26.31 | 454.80 | 0.3410 | 1176.30 | 0.5843 | 0.4963 | 212.32 | 5108.40 | 0.7439 | 68.1589 | 1.8178 | 0.0853 | 16.923 | 8.122 | 0.4671 | 0.00377 | 12671 | − |
| | 673 | 6 | *pU-21B* | | M Homo | 3 | 15 | 154.33 | 4.60 | 130.33 | 9.73 | 7.53 | 2.73 | 334.00 | 7.66 | 18.24 | 22.38 | 23.09 | 26.13 | 440.10 | 0.3330 | 1151.50 | 0.5563 | 0.4833 | 196.59 | 5278.11 | 0.7636 | 64.5070 | 1.8436 | 0.0995 | 15.710 | 7.118 | 0.5083 | 0.00403 | 10292 | |
| | | B6;CB-*Lpar5Gt(pU-21B)206Imeg* | | | F WT | 3 | 15-16 | 151.00 | 4.17 | 116.00 | 9.63 | 5.87 | 2.43 | 451.67 | 7.85 | 16.17 | 18.96 | 21.40 | 21.30 | 372.40 | 0.0845 | 1205.93 | 0.5333 | 0.4424 | 207.00 | 4714.51 | 0.2208 | 57.0984 | 0.8781 | 0.2232 | 15.348 | 8.704 | 0.3475 | 0.00266 | 11967 | |
| | | | | | F Homo | 3 | 14-15 | 152.67 | 3.63 | 117.67 | 9.67 | 5.23 | 2.60 | 468.33 | 4.75 | 14.02 | 18.18 | 20.33 | 21.79 | 411.83 | 0.1373 | 1185.37 | 0.5343 | 0.4508 | 207.17 | 4772.58 | 0.3331 | 61.5664 | 1.1998 | 0.1412 | 15.420 | 8.514 | 0.3393 | 0.00263 | 12870 | |
| | | | | 【SD】 | M WT | | | 2.89 | 0.15 | 3.06 | 0.31 | 1.50 | 0.06 | 103.36 | 1.51 | 3.14 | 0.94 | 0.61 | 1.52 | 63.83 | 0.1187 | 24.60 | 0.0789 | 0.0568 | 20.45 | 234.29 | 0.1998 | 8.5663 | 0.0774 | 0.0095 | 3.676 | 1.417 | 0.0672 | 0.00136 | 2021 | |
| | | | | | M Homo | | | 4.51 | 0.61 | 19.22 | 0.23 | 1.79 | 0.23 | 39.28 | 1.84 | 3.27 | 2.48 | 2.49 | 2.23 | 1.73 | 0.1009 | 1.11 | 0.0650 | 0.0569 | 8.37 | 505.24 | 0.2328 | 1.5743 | 0.2452 | 0.0215 | 2.580 | 1.118 | 0.0634 | 0.00102 | 2074 | |
| | | | | | F WT | | | 0.00 | 0.40 | 1.00 | 0.06 | 1.39 | 0.31 | 31.01 | 1.31 | 2.84 | 1.28 | 1.39 | 0.93 | 16.34 | 0.0239 | 12.16 | 0.0300 | 0.0286 | 4.92 | 268.02 | 0.0531 | 2.6096 | 0.0584 | 0.0126 | 0.689 | 0.584 | 0.0624 | 0.00054 | 1210 | |
| | | | | | F Homo | | | 0.58 | 0.21 | 0.58 | 0.21 | 0.31 | 0.17 | 9.29 | ND | 1.25 | 0.23 | 0.99 | 0.45 | 3.54 | 0.0086 | 11.06 | 0.0067 | 0.0032 | 4.57 | 51.04 | 0.0202 | 0.8497 | 0.0752 | 0.0174 | 0.641 | 0.532 | 0.0269 | 0.00022 | 1369 | |
| 9 | 21-KBT38 | *Ankrd11* | *KAB6* | 【Mean】 | M WT | 4 | 15 | 150.00 | 4.55 | 91.00 | 10.13 | 5.60 | 2.53 | 327.00 | 5.74 | 13.43 | 21.26 | 24.22 | 27.56 | 465.60 | 0.3560 | 1122.30 | 0.5548 | 0.4937 | 207.39 | 5146.62 | 0.7606 | 63.1273 | 2.0060 | 0.0661 | 17.433 | 8.240 | 0.5123 | 0.00291 | 23406 | + |
| | 1212 | 8 | *pU-21T* | | M Hetero | 3 | 15 | 152.67 | 4.83 | 115.33 | 9.87 | 6.20 | 2.30 | 355.67 | 5.32 | 8.85 | 17.46 | 20.73 | 24.65 | 482.87 | 0.3280 | 1100.37 | 0.5327 | 0.4749 | 204.93 | 4988.87 | 0.6613 | 61.6853 | 1.9462 | 0.0745 | 16.402 | 8.062 | 0.6782 | 0.00279 | 17985 | |
| | | B6-*Ankrd11Gt(pU-21KBT)38Card* | | | F WT | 3 | 16 | 148.67 | 3.43 | 113.33 | 9.97 | 4.10 | 2.23 | 413.33 | 5.54 | 14.37 | 17.86 | 19.67 | 21.45 | 396.93 | 0.0779 | 1132.40 | 0.5203 | 0.4591 | 204.78 | 4865.78 | 0.1918 | 52.5181 | 0.8672 | 0.2951 | 15.265 | 8.096 | 0.5279 | 0.00260 | 22236 | |
| | | | | | F Hetero | 3 | 16 | 148.67 | 4.17 | 114.00 | 9.90 | 4.70 | 2.43 | 513.33 | 5.25 | 11.96 | 15.17 | 17.53 | 19.56 | 399.73 | 0.0676 | 1076.77 | 0.4330 | 0.4015 | 189.64 | 4561.58 | 0.1676 | 54.5721 | 0.8929 | 0.2828 | 14.280 | 8.624 | 0.3209 | 0.00216 | 17934 | |
| | | | | 【SD】 | M WT | | | 0.82 | 0.51 | 50.67 | 0.19 | 1.14 | 0.22 | 14.99 | 1.05 | 3.82 | 3.07 | 2.33 | 1.82 | 41.67 | 0.1273 | 86.41 | 0.0890 | 0.0621 | 16.51 | 278.92 | 0.2027 | 3.1648 | 0.2376 | 0.0040 | 2.854 | 0.863 | 0.3010 | 0.00122 | 3743 | |
| | | | | | M Hetero | | | 1.15 | 0.15 | 2.52 | 0.21 | 0.10 | 0.20 | 104.55 | 0.28 | 2.00 | 2.08 | 2.03 | 2.98 | 71.59 | 0.1686 | 125.60 | 0.1860 | 0.1237 | 43.97 | 510.62 | 0.2838 | 10.2860 | 0.2630 | 0.0196 | 5.443 | 1.362 | 0.1245 | 0.00156 | 2473 | |
| | | | | | F WT | | | 1.53 | 0.59 | 1.15 | 0.06 | 0.30 | 0.21 | 77.47 | 0.50 | 4.26 | 1.43 | 1.39 | 1.48 | 12.23 | 0.0187 | 101.58 | 0.0585 | 0.0142 | 8.07 | 49.21 | 0.0412 | 3.1642 | 0.0882 | 0.0290 | 1.200 | 0.485 | 0.2098 | 0.00033 | 2095 | |
| | | | | | F Hetero | | | 1.15 | 0.78 | 2.00 | 0.20 | 0.53 | 0.21 | 62.29 | 0.62 | 4.14 | 0.75 | 1.78 | 1.95 | 12.71 | 0.0093 | 54.18 | 0.0408 | 0.0181 | 11.07 | 41.75 | 0.0276 | 3.0366 | 0.2158 | 0.0878 | 1.021 | 0.618 | 0.1263 | 0.00033 | 4051 | |
| 10 | 21-KBW122 | *Lima1* | *KAB6* | 【Mean】 | M WT | 2 | 15 | 151.50 | 4.20 | 116.00 | 9.90 | 7.95 | 2.30 | 281.50 | 7.95 | 18.58 | 23.72 | ND | 28.76 | 514.35 | 0.8295 | 1193.05 | 0.6830 | 0.5725 | 231.56 | 5492.19 | 1.6565 | 78.4833 | 2.7835 | 0.0224 | 23.560 | 9.825 | 0.8782 | 0.00639 | 9130 | − |
| | 1305 | 15 | *pU-21W* | | M Homo | 4 | 14-16 | 152.50 | 4.40 | 115.75 | 9.65 | 6.63 | 2.63 | 377.75 | 4.96 | 10.67 | 17.77 | 19.79 | 21.47 | 449.70 | 0.3410 | 1155.68 | 0.5043 | 0.4367 | 197.58 | 4850.30 | 0.7644 | 67.5866 | 1.8652 | 0.0726 | 12.545 | 6.708 | 1.0212 | 0.00354 | 4161 | |
| | | B6-*Lima1Gt(pU-21KBW)122Card* | | | F WT | 3 | 14-16 | 148.00 | 3.47 | 110.67 | 9.23 | 6.83 | 2.37 | 391.67 | 6.27 | ND | 16.01 | 19.49 | 20.68 | 403.77 | 0.0907 | 1193.67 | 0.5230 | 0.4381 | 208.48 | 4656.17 | 0.2203 | 57.0610 | 0.8814 | 0.2883 | 16.547 | 9.705 | 0.8112 | 0.00316 | 9371 | |
| | | | | | F Homo | 5 | 14-16 | 149.40 | 3.70 | 115.00 | 9.32 | 7.64 | 2.46 | 406.40 | 6.37 | 11.70 | 16.65 | 19.14 | 20.95 | 394.86 | 0.1500 | 1153.86 | 0.5078 | 0.4400 | 204.67 | 4731.24 | 0.3746 | 58.9463 | 1.2627 | 0.1577 | 14.087 | 7.905 | 0.9531 | 0.00370 | 6114 | |
| | | | | 【SD】 | M WT | | | 0.71 | 0.42 | 1.41 | 0.00 | 0.78 | 0.28 | 21.92 | 0.28 | 1.53 | 0.33 | ND | 0.88 | 5.16 | 0.1648 | 3.61 | 0.0212 | 0.0200 | 10.75 | 27.14 | 0.3494 | 1.7390 | 0.4600 | 0.0120 | 4.554 | 1.994 | 0.5209 | 0.00118 | 7517 | |
| | | | | | M Homo | | | 2.65 | 0.47 | 1.26 | 0.24 | 0.78 | 0.35 | 127.66 | 0.63 | 1.71 | 2.13 | 3.85 | 3.18 | 24.68 | 0.0667 | 25.85 | 0.0368 | 0.0376 | 7.64 | 302.39 | 0.1348 | 4.5405 | 0.2395 | 0.0303 | 1.798 | 0.831 | 0.1609 | 0.00062 | 1167 | |
| | | | | | F WT | | | 2.00 | 0.29 | 2.52 | 0.15 | 0.90 | 0.06 | 35.16 | ND | ND | 1.12 | 1.10 | 1.35 | 11.16 | 0.0108 | 15.21 | 0.0303 | 0.0203 | 6.41 | 204.00 | 0.0316 | 1.1381 | 0.1123 | 0.0530 | 3.234 | 2.638 | 0.1735 | 0.00058 | 4096 | |
| | | | | | F Homo | | | 1.14 | 0.59 | 1.58 | 0.31 | 1.51 | 0.34 | 41.26 | 1.59 | ND | 2.26 | 1.77 | 2.70 | 13.15 | 0.0298 | 7.46 | 0.0326 | 0.0269 | 5.25 | 167.92 | 0.0656 | 1.6572 | 0.1577 | 0.0362 | 1.027 | 0.327 | 0.1447 | 0.00067 | 3453 | |
| 11 | 21-KBW180 | *Cnn3* | *KAB6* | 【Mean】 | M WT | 3 | 15 | 149.67 | 3.87 | 109.33 | 9.03 | 7.93 | 2.90 | 240.67 | 6.24 | 14.21 | 20.51 | 23.83 | 26.11 | 455.70 | 0.3057 | 1158.47 | 0.5667 | 0.4891 | 211.58 | 5056.98 | 0.6613 | 65.4136 | 1.5690 | 0.1539 | 18.837 | 9.254 | 0.7184 | 0.00528 | 10140 | + |
| | 2050 | 3 | *pU-21W* | | M Hetero | 3 | 15 | 149.33 | 4.10 | 110.67 | 8.83 | 9.10 | 2.60 | 294.00 | ND | 14.34 | 19.36 | 23.53 | 26.39 | 500.37 | 0.4010 | 1157.77 | 0.6067 | 0.5241 | 221.83 | 5253.51 | 0.8136 | 77.5928 | 1.5862 | 0.1588 | 19.510 | 8.943 | 0.7744 | 0.00486 | 12902 | |
| | | B6-*Cnn3Gt(pU-21KBW)180Card* | | | F WT | 3 | 15 | 150.00 | 4.33 | 112.67 | 8.77 | 8.80 | 2.40 | 500.00 | 6.00 | 14.43 | 18.02 | 20.38 | 22.23 | 373.40 | 0.0746 | 1155.60 | 0.4850 | 0.4184 | 203.41 | 4560.12 | 0.1889 | 52.4799 | 0.7674 | 0.3313 | 18.743 | 11.316 | 0.5761 | 0.00330 | 13160 | |
| | | | | | F Hetero | 3 | 14-15 | 151.00 | 4.07 | 113.67 | 8.80 | 8.63 | 2.23 | 476.67 | 6.05 | 10.72 | 15.88 | 17.57 | 18.87 | 358.13 | 0.0723 | 1135.43 | 0.4653 | 0.4096 | 195.60 | 4593.29 | 0.1920 | 51.0097 | 0.8376 | 0.2843 | 18.215 | 10.885 | 0.6539 | 0.00298 | 13554 | |
| | | | | 【SD】 | M WT | | | 0.58 | 0.15 | 2.08 | 0.47 | 0.31 | 0.26 | 34.93 | 0.11 | 2.17 | 1.11 | 1.36 | 1.88 | 43.34 | 0.1684 | 15.97 | 0.0515 | 0.0424 | 4.33 | 326.22 | 0.3236 | 6.9461 | 0.4734 | 0.0641 | 4.117 | 1.722 | 0.2160 | 0.00226 | 5305 | |
| | | | | | M Hetero | | | 1.15 | 0.53 | 1.15 | 0.06 | 0.20 | 0.36 | 49.12 | ND | 5.77 | 3.36 | 3.89 | 4.09 | 58.86 | 0.0701 | 57.01 | 0.0308 | 0.0156 | 26.47 | 441.28 | 0.1168 | 7.5564 | 0.1157 | 0.0634 | 1.498 | 0.967 | 0.2273 | 0.00195 | 1482 | |
| | | | | | F WT | | | 0.00 | 0.15 | 1.15 | 0.25 | 0.66 | 0.17 | 57.30 | ND | 4.34 | 1.88 | 1.81 | 1.81 | 32.77 | 0.0526 | 13.28 | 0.0289 | 0.0212 | 8.58 | 141.56 | 0.1222 | 6.0126 | 0.3526 | 0.1339 | 2.709 | 1.495 | 0.2838 | 0.00062 | 2652 | |
| | | | | | F Hetero | | | 1.73 | 0.06 | 1.15 | 0.26 | 0.35 | 0.12 | 18.56 | 0.98 | 0.38 | 1.53 | 0.84 | 1.22 | 25.04 | 0.0426 | 15.91 | 0.0518 | 0.0402 | 7.44 | 256.01 | 0.1111 | 5.0720 | 0.3282 | 0.1668 | 0.737 | 0.788 | 0.1410 | 0.00078 | 1407 | |
| 12 | 21-KBW205 | *Wdr11* | *KAB6* | 【Mean】 | M WT | 3 | 15 | 151.67 | 4.50 | 112.00 | 8.63 | 7.40 | 2.40 | 328.67 | 6.87 | 14.23 | 22.26 | 25.29 | 27.98 | 417.87 | 0.4080 | 1113.30 | 0.6057 | 0.5450 | 213.46 | 5496.96 | 0.9466 | 84.2956 | 1.6164 | 0.1365 | 21.718 | 8.979 | 0.5674 | 0.00364 | 15954 | + |
| | 2083 | 7 | *pU-21W* | | M Hom | 3 | 14-15 | 151.67 | 4.83 | 112.00 | 9.00 | 8.87 | 2.43 | 367.00 | 4.77 | 8.01 | 16.71 | 20.21 | 21.59 | 396.10 | 0.2908 | 1132.07 | 0.5292 | 0.4668 | 206.75 | 4938.17 | 0.5180 | 72.1223 | 1.2652 | 0.1548 | 16.640 | 8.604 | 0.4044 | 0.00259 | 12852 | |
| | | B6-*Wdr11Gt(pU-21KBW)205Card* | | | F WT | 3 | 15 | 151.00 | 4.10 | 113.33 | 9.17 | 8.37 | 2.10 | 478.67 | 6.83 | 12.21 | 17.57 | 18.80 | 20.29 | 342.57 | 0.0656 | 1159.57 | 0.4893 | 0.4220 | 205.38 | 4560.03 | 0.1467 | 65.6145 | 0.5162 | 0.4532 | 17.615 | 10.653 | 0.3307 | 0.00278 | 14266 | |
| | | | | | F Hom | 3 | 15-16 | 151.00 | 4.08 | 114.50 | 8.75 | 7.65 | 2.08 | 493.00 | 4.54 | 8.43 | 13.99 | 15.79 | 16.80 | 331.63 | 0.0578 | 1151.93 | 0.4424 | 0.3840 | 201.86 | 4243.57 | 0.1732 | 64.8332 | 0.6904 | 0.2709 | 19.428 | 13.559 | 0.4298 | 0.00410 | 12488 | |
| | | | | 【SD】 | M WT | | | 0.58 | 0.46 | 1.73 | 0.58 | 1.81 | 0.36 | 25.79 | 0.33 | 0.60 | 1.88 | 2.52 | 3.10 | 53.79 | 0.2094 | 21.45 | 0.0740 | 0.0758 | 5.40 | 550.02 | 0.4208 | 9.2227 | 0.2464 | 0.0128 | 5.264 | 1.256 | 0.1516 | 0.00030 | 5206 | |
| | | | | | M Hom | | | 1.53 | 0.85 | 1.00 | 0.44 | 0.74 | 0.06 | 42.30 | 0.76 | 1.78 | 0.82 | 0.80 | 0.79 | 55.28 | 0.1443 | 26.86 | 0.0586 | 0.0414 | 14.29 | 143.65 | 0.3965 | 13.5622 | 0.5333 | 0.0950 | 2.556 | 1.502 | 0.1654 | 0.00061 | 2284 | |
| | | | | | F WT | | | 1.00 | 0.30 | 1.53 | 0.15 | 0.32 | 0.00 | 19.86 | 0.63 | 0.91 | 0.40 | 0.21 | 0.26 | 23.49 | 0.0129 | 9.19 | 0.0062 | 0.0041 | 3.52 | 23.65 | 0.0645 | 1.0086 | 0.1712 | 0.1548 | 2.089 | 1.355 | 0.0699 | 0.00029 | 1427 | |
| | | | | | F Hom | | | 1.15 | 0.17 | 2.38 | 0.24 | 0.53 | 0.10 | 44.77 | 0.99 | 1.75 | 1.41 | 1.56 | 1.05 | 13.47 | 0.0060 | 15.53 | 0.0126 | 0.0105 | 3.88 | 68.89 | 0.0233 | 0.7941 | 0.1502 | 0.0455 | 1.854 | 1.296 | 0.1503 | 0.00213 | 5152 | |
| 13 | 21-T152 | *Tctn1* | *KTPU8* | 【Mean】 | M WT | 3 | 10-11 | 146.67 | 5.90 | 111.67 | 10.03 | 8.73 | 2.77 | 351.33 | 6.86 | 13.52 | 23.04 | 25.77 | 28.68 | 479.43 | 0.4680 | 1135.03 | 0.6016 | 0.5302 | 217.17 | 5341.62 | 0.9900 | 73.7336 | 2.0348 | 0.1101 | 20.508 | 8.912 | 0.7307 | 0.00420 | 23255 | + |
| | 1074 | 5 | *pU-21T* | | M Hetero | 3 | 10-11 | 148.33 | 3.90 | 111.00 | 10.17 | 6.93 | 2.57 | 374.33 | 6.76 | 12.21 | 22.22 | 25.41 | 27.16 | 524.47 | 0.7177 | 1174.23 | 0.6313 | 0.5378 | 233.51 | 5147.96 | 1.3818 | 76.7932 | 2.4539 | 0.0602 | 20.703 | 9.818 | 0.5499 | 0.00355 | 26689 | |
| | | B6;CB-*Tctn1Gt(pU-21T)152Imeg* | | | F WT | 3 | 11 | 147.67 | 3.77 | 113.33 | 9.87 | 5.20 | 2.53 | 457.67 | 7.52 | 13.22 | 19.42 | 21.86 | 23.87 | 400.93 | 0.0920 | 1146.57 | 0.4788 | 0.4176 | 193.28 | 4703.11 | 0.2251 | 53.9121 | 1.1414 | 0.2586 | 12.808 | 7.278 | 0.5476 | 0.00198 | 17265 | |
| | | | | | F Hetero | 3 | 11 | 149.00 | 3.33 | 113.33 | 9.83 | 5.57 | 2.43 | 488.00 | ND | ND | ND | ND | ND | 388.60 | 0.0984 | 896.43 | 0.3640 | 0.4255 | 195.64 | 4758.85 | 0.2484 | 34.7215 | 1.0757 | 0.2088 | 13.703 | 7.632 | 0.4463 | 0.00214 | 14611 | |
| | | | | 【SD】 | M WT | | | 1.15 | 3.56 | 2.89 | 0.15 | 3.27 | 0.71 | 89.20 | 0.66 | 1.24 | 0.73 | 0.82 | 1.19 | 35.65 | 0.0792 | 45.37 | 0.0617 | 0.0515 | 11.55 | 343.08 | 0.1416 | 6.3768 | 0.1274 | 0.0455 | 4.962 | 1.143 | 0.0264 | 0.00098 | 5738 | |
| | | | | | M Hetero | | | 1.15 | 0.14 | 0.00 | 0.45 | 1.07 | 0.59 | 19.14 | 0.00 | 0.68 | 0.95 | 0.73 | ND | 28.55 | 0.4348 | 9.76 | 0.0623 | 0.0555 | 13.99 | 273.74 | 0.7656 | 9.2261 | 0.4668 | 0.0145 | 1.723 | 0.375 | 0.0927 | 0.00056 | 3650 | |
| | | | | | F WT | | | 1.15 | 0.31 | 1.53 | 0.35 | 0.26 | 0.06 | 51.43 | ND | ND | ND | ND | ND | 18.63 | 0.0196 | 19.87 | 0.0294 | 0.0259 | 6.68 | 294.06 | 0.0482 | 1.4569 | 0.2917 | 0.0856 | 1.178 | 0.267 | 0.0418 | 0.00025 | 3720 | |
| | | | | | F Hetero | | | 1.00 | 0.15 | 0.58 | 0.15 | 0.15 | 0.12 | 24.33 | ND | ND | ND | ND | ND | 4.95 | 0.0303 | 435.83 | 0.2043 | 0.0311 | 13.93 | 184.53 | 0.0729 | 30.0808 | 0.1427 | 0.0307 | 0.500 | 0.721 | 0.0463 | 0.00016 | 2844 | |
| 14 | 21-T167 | *2500002B13Rik; lincRNA* | *KTPU8* | 【Mean】 | M WT | 3 | 14 | 153.33 | 4.62 | 107.87 | 9.17 | 9.40 | ND | 333.00 | 8.50 | 17.18 | 24.64 | 26.90 | 29.80 | 431.97 | 0.3693 | 1115.13 | 0.5323 | 0.4780 | 203.47 | 5084.01 | 0.8471 | 70.7224 | 1.6438 | 0.1236 | 16.537 | 8.046 | 0.6079 | 0.00400 | 7915 | + |
| | 1774 | 8 | *pU-21T* | | M Hom | 3 | 14 | 154.77 | 4.66 | 109.03 | 9.10 | 9.17 | ND | 271.67 | 8.40 | 18.37 | 26.01 | 28.37 | 30.83 | 446.87 | 0.3797 | 1154.97 | 0.6377 | 0.5526 | 236.49 | 5202.04 | 0.8288 | 71.4281 | 1.7770 | 0.1013 | 17.808 | 8.369 | 0.5781 | 0.00364 | 10154 | |
| | | B6;CB-*2500002B13RikGt(pU-21T)167Card* | | | F WT | 3 | 15 | 151.13 | 4.02 | 110.03 | 8.93 | 7.87 | ND | 368.00 | 7.13 | 15.06 | 19.17 | 21.50 | 21.99 | 384.60 | 0.0829 | 1159.80 | 0.4920 | 0.4241 | 207.80 | 4539.40 | 0.2117 | 57.5585 | 0.8397 | 0.2525 | 16.268 | 9.862 | 0.3521 | 0.00249 | 11837 | |
| | | | | | F Hom | 3 | 15 | 152.27 | 3.93 | 111.50 | 9.07 | 8.53 | ND | 359.67 | 6.87 | 14.62 | 18.45 | 19.70 | 21.01 | 401.83 | 0.0747 | 1186.70 | 0.5210 | 0.4390 | 218.04 | 4523.44 | 0.1826 | 58.4991 | 0.7928 | 0.2980 | 18.495 | 11.362 | 0.5081 | 0.00345 | 11603 | |
| | | | | 【SD】 | M WT | | | 0.55 | 0.17 | 1.12 | 0.06 | 0.61 | ND | 26.15 | 1.21 | 0.70 | 1.83 | 2.21 | 2.11 | 52.36 | 0.2387 | 28.88 | 0.0161 | 0.0262 | 4.29 | 249.85 | 0.5613 | 8.0747 | 0.6767 | 0.0867 | 1.357 | 0.507 | 0.1196 | 0.00052 | 2103 | |
| | | | | | M Hom | | | 0.35 | 0.10 | 0.40 | 0.10 | 0.29 | ND | 61.91 | 0.88 | 1.15 | 1.33 | 2.22 | 1.49 | 49.15 | 0.2146 | 32.27 | 0.1071 | 0.0972 | 18.94 | 645.37 | 0.4348 | 11.0629 | 0.4130 | 0.0479 | 7.216 | 3.348 | 0.0617 | 0.00088 | 5048 | |
| | | | | | F WT | | | 0.06 | 0.18 | 1.20 | 0.45 | 0.49 | ND | 14.42 | 0.30 | 0.21 | 0.14 | 0.42 | 0.05 | 18.73 | 0.0101 | 17.46 | 0.0295 | 0.0200 | 5.91 | 105.16 | 0.0361 | 1.3845 | 0.1506 | 0.0458 | 3.816 | 1.825 | 0.0828 | 0.00056 | 5056 | |
| | | | | | F Hom | | | 0.90 | 0.43 | 1.31 | 0.23 | 1.55 | ND | 34.31 | 0.55 | 0.47 | 1.47 | 0.81 | 0.90 | 16.82 | 0.0093 | 0.44 | 0.0279 | 0.0235 | 5.31 | 130.68 | 0.0316 | 1.8774 | 0.2059 | 0.0850 | 1.298 | 0.713 | 0.0254 | 0.00075 | 2842 | |
| 15 | 21-T188 | *EST* | *KTPU8* | 【Mean】 | M WT | 3 | 14 | 151.33 | 4.20 | 111.67 | 9.10 | 8.77 | 2.73 | 322.33 | 5.76 | 13.52 | 20.28 | 23.57 | 26.24 | 421.87 | 0.4820 | 1127.33 | 0.5760 | 0.5097 | 226.47 | 4995.56 | 1.1069 | 88.3768 | 1.8192 | 0.1024 | 19.650 | 235.800 | 0.6159 | 0.00444 | 11721 | − |
| | 1045 | 17 | *pU-21T* | | M Hom | 3 | 14 | 154.00 | 5.27 | 112.33 | 9.57 | 9.47 | 2.93 | 344.33 | 5.00 | 10.29 | 18.12 | 22.80 | 25.27 | 459.17 | 0.4183 | 1140.17 | 0.5633 | 0.4941 | 227.26 | 4886.56 | 0.9288 | 95.1390 | 1.6200 | 0.1005 | 16.718 | 200.620 | 0.4427 | 0.00273 | 11289 | |
| | | B6-*Gt(pU-21T)188Imeg* | | | F WT | 3 | 14-15 | 151.67 | 4.17 | 116.00 | 9.23 | 8.37 | 2.63 | 434.67 | 6.69 | 14.17 | 17.61 | 19.24 | 20.91 | 340.90 | 0.0845 | 1143.20 | 0.4813 | 0.4210 | 213.20 | 4418.66 | 0.2424 | 67.5692 | 0.8802 | 0.2441 | 16.718 | 200.620 | 0.4368 | 0.00275 | 13094 | |
| | | | | | F Hom | 3 | 15 | 151.67 | 4.17 | 113.67 | 9.37 | 8.60 | 2.40 | 455.00 | 5.16 | 11.72 | 16.76 | 18.88 | 19.94 | 343.97 | 0.0916 | 1144.10 | 0.4733 | 0.4138 | 209.81 | 4411.07 | 0.2610 | 68.8172 | 0.8448 | 0.2517 | 17.010 | 204.120 | 0.5175 | 0.00287 | 13512 | |
| | | | | 【SD】 | M WT | | | 2.08 | 0.60 | 1.53 | 0.17 | 0.81 | 0.25 | 6.51 | 1.44 | 1.99 | 4.07 | 3.83 | 4.48 | 49.53 | 0.3040 | 24.90 | 0.1161 | 0.0930 | 25.76 | 462.47 | 0.6578 | 14.7728 | 0.6675 | 0.0947 | 4.745 | 56.936 | 0.1036 | 0.00151 | 6407 | |
| | | | | | M Hom | | | 2.00 | 0.90 | 2.08 | 0.31 | 0.71 | 0.25 | 5.77 | 0.83 | 0.48 | 0.95 | 0.74 | 0.72 | 4.14 | 0.1061 | 12.85 | 0.0247 | 0.0268 | 2.54 | 242.33 | 0.2423 | 2.2209 | 0.3432 | 0.0444 | 2.051 | 24.612 | 0.0870 | 0.00039 | 3603 | |
| | | | | | F WT | | | 1.53 | 0.40 | 2.65 | 0.31 | 1.19 | 0.25 | 43.41 | 0.49 | 1.50 | 0.88 | 0.45 | 0.70 | 15.08 | 0.0060 | 7.09 | 0.0201 | 0.0156 | 8.09 | 43.26 | 0.0128 | 1.1091 | 0.1081 | 0.0216 | 1.070 | 12.840 | 0.1306 | 0.00013 | 684 | |
| | | | | | F Hom | | | 1.15 | 0.85 | 0.58 | 0.57 | 1.76 | 0.10 | 43.51 | 1.16 | 1.98 | 1.18 | 0.35 | 0.30 | 6.07 | 0.0073 | 5.37 | 0.0049 | 0.0061 | 4.29 | 98.42 | 0.0224 | 1.6739 | 0.0594 | 0.0655 | 1.456 | 17.468 | 0.0830 | 0.00033 | 1430 | |
| 16 | 21-T2 | *Msi2* | *KTPU8* | 【Mean】 | M WT | 3 | 15-16 | 150.67 | 3.97 | 113.00 | 9.77 | 5.37 | 2.40 | 248.33 | 6.11 | 12.24 | 21.15 | 24.13 | 27.34 | 529.97 | 0.5501 | 1195.63 | 0.6532 | 0.5460 | 233.30 | 5216.91 | 1.0607 | 78.9576 | 2.0230 | 0.0719 | 19.227 | 8.893 | 0.5381 | 0.00380 | 12117 | + |
| | 836 | 11 | *pU-21T* | | M Homo | 3 | 15-16 | 149.33 | 5.00 | 115.00 | 9.50 | 7.30 | 2.47 | 301.00 | 4.87 | 8.21 | 15.87 | 18.77 | 20.12 | 478.70 | 0.2181 | 1170.60 | 0.4436 | 0.3782 | 199.68 | 4222.81 | 0.4501 | 68.3271 | 1.3500 | 0.1605 | 12.665 | 8.901 | 0.4004 | 0.00240 | 8813 | |
| | | B6;CB-*Msi2Gt(pU-21T)2Imeg* | | | F WT | 3 | 14-15 | 149.67 | 3.67 | 114.33 | 9.47 | 5.76 | 2.40 | 398.00 | 7.43 | 14.53 | 19.03 | 20.58 | 21.95 | 395.80 | 0.1178 | 1160.90 | 0.4824 | 0.4156 | 203.20 | 4532.06 | 0.2919 | 56.3470 | 1.0652 | 0.2096 | 12.798 | 7.845 | 0.3576 | 0.00242 | 9906 | |
| | | | | | F Homo | 3 | 14-15 | 152.33 | 3.90 | 115.67 | 9.67 | 6.50 | 2.07 | 435.33 | 5.51 | 9.90 | 15.79 | 17.93 | 18.72 | 395.13 | 0.0989 | 1157.07 | 0.4070 | 0.3514 | 186.05 | 4156.74 | 0.2473 | 56.7065 | 1.0639 | 0.1883 | 10.686 | 7.768 | 0.3581 | 0.00129 | 7940 | |
| | | | | 【SD】 | M WT | | | 1.53 | 0.29 | 1.00 | 0.29 | 0.67 | 0.26 | 8.14 | 1.27 | 4.43 | 3.15 | 2.16 | 1.89 | 19.83 | 0.0704 | 22.30 | 0.0442 | 0.0271 | 18.15 | 52.28 | 0.1035 | 5.0659 | 0.2610 | 0.0322 | 2.209 | 1.205 | 0.0627 | 0.00061 | 1094 | |
| | | | | | M Homo | | | 2.08 | 0.98 | 2.65 | 0.44 | 2.31 | 0.51 | 16.37 | 0.66 | 1.65 | 1.31 | 1.48 | 1.43 | 45.07 | 0.1747 | 33.62 | 0.0642 | 0.0452 | 20.10 | 284.53 | 0.3381 | 5.9998 | 0.5341 | 0.0701 | 1.694 | 0.096 | 0.0906 | 0.00062 | 1487 | |
| | | | | | F WT | | | 1.53 | 0.86 | 1.53 | 0.47 | 1.49 | 0.10 | 41.39 | 1.84 | 1.83 | 0.31 | 0.29 | 1.06 | 11.36 | 0.0427 | 30.07 | 0.0313 | 0.0252 | 9.18 | 145.93 | 0.0988 | 4.4204 | 0.1799 | 0.0368 | 0.360 | 0.515 | 0.0609 | 0.00044 | 677 | |
| | | | | | F Homo | | | 2.08 | 0.20 | 0.58 | 0.21 | 0.44 | 0.15 | 57.73 | 0.91 | 1.46 | 1.14 | 0.69 | 0.14 | 9.70 | 0.0288 | 28.60 | 0.0342 | 0.0212 | 10.80 | 72.56 | 0.0692 | 2.1398 | 0.3112 | 0.0570 | 1.421 | 0.968 | 0.1000 | 0.00026 | 1138 | |
| 17 | 21-T239 | *Wdfy1* | *KTPU8* | 【Mean】 | M WT | 3 | 13-14 | 150.00 | 4.17 | 112.33 | 9.80 | 6.63 | 2.50 | 287.33 | 5.61 | 11.32 | 22.10 | 24.67 | 27.26 | 511.70 | 0.7920 | 1168.53 | 0.6720 | 0.5751 | 223.38 | 5597.03 | 1.5837 | 77.5573 | 2.6419 | 0.0344 | 20.547 | 8.230 | 0.8364 | 0.00507 | 2881 | + |
| | 1082 | 1 | *pU-21T* | | M Hom | 3 | 13-15 | 150.67 | 4.30 | 111.67 | 10.07 | 7.37 | 2.77 | 322.00 | 4.69 | 8.58 | 18.25 | 21.73 | 25.27 | 519.30 | 0.7010 | 1164.30 | 0.6503 | 0.5583 | 224.85 | 5430.77 | 1.3799 | 75.7788 | 2.6066 | 0.0475 | 20.168 | 8.677 | 1.5316 | 0.00486 | 2484 | |
| | | B6;CB-*Wdfy1Gt(pU-21T)239Imeg* | | | F WT | 4 | 13-14 | 151.00 | 3.40 | 116.00 | 9.38 | 5.28 | 2.35 | 465.75 | 5.57 | 10.74 | 18.28 | 19.11 | 19.44 | 420.25 | 0.1470 | 1172.50 | 0.5168 | 0.4405 | 201.89 | 4787.48 | 0.3467 | 59.5336 | 1.2756 | 0.1614 | 13.561 | 7.464 | 1.7409 | 0.00266 | 19378 | |
| | | | | | F Hom | 4 | 13-14 | 151.25 | 3.88 | 115.50 | 9.63 | 6.28 | 2.40 | 475.25 | 4.74 | 8.73 | 16.68 | 18.39 | 17.24 | 382.80 | 0.1249 | 1150.45 | 0.4455 | 0.3870 | 184.14 | 4537.96 | 0.3146 | 58.0918 | 1.3432 | 0.1459 | 14.035 | 8.608 | 1.3854 | 0.00297 | 15945 | |
| | | | | 【SD】 | M WT | | | 2.65 | 0.35 | 3.21 | 0.66 | 2.02 | 0.44 | 41.36 | 1.32 | 2.09 | 1.71 | 0.42 | 0.11 | 12.23 | 0.1424 | 4.91 | 0.0366 | 0.0316 | 8.00 | 168.10 | 0.2516 | 4.2083 | 0.1016 | 0.0059 | 1.901 | 0.382 | 0.2911 | 0.00091 | 3120 | |
| | | | | | M Hom | | | 1.53 | 0.50 | 1.15 | 0.21 | 1.78 | 0.31 | 38.97 | 1.01 | 1.62 | 0.44 | 1.73 | 0.42 | 17.88 | 0.1528 | 18.84 | 0.0539 | 0.0393 | 28.17 | 272.66 | 0.2619 | 0.7516 | 0.2110 | 0.0254 | 1.739 | 1.534 | 0.3479 | 0.00112 | 2674 | |
| | | | | | F WT | | | 1.41 | 0.36 | 1.63 | 0.29 | 1.20 | 0.25 | 31.72 | 0.60 | 1.81 | 0.82 | 1.23 | 0.13 | 14.18 | 0.0385 | 18.91 | 0.0204 | 0.0111 | 10.11 | 128.43 | 0.0828 | 3.8988 | 0.2333 | 0.0376 | 1.342 | 0.994 | 0.5471 | 0.00059 | 5262 | |
| | | | | | F Hom | | | 1.50 | 0.22 | 1.73 | 0.21 | 1.26 | 0.18 | 95.78 | 1.20 | 1.12 | 1.78 | 1.66 | ND | 35.73 | 0.0611 | 24.84 | 0.0442 | 0.0304 | 14.94 | 108.22 | 0.1357 | 4.2994 | 0.3323 | 0.0694 | 1.959 | 1.479 | 0.4779 | 0.00094 | 5173 | |
| 18 | 21-T269 | *Nedd4* | *KTPU8* | 【Mean】 | M WT | 6 | 14 | 149.33 | 3.90 | 112.67 | 9.75 | 6.77 | 2.43 | 258.67 | 5.74 | 13.43 | 21.26 | 24.22 | 27.56 | 515.28 | 0.5500 | 1176.17 | 0.6648 | 0.5652 | 234.16 | 5381.40 | 1.0875 | 74.9115 | 2.0635 | 0.0824 | 21.349 | 9.294 | 1.0164 | 0.00528 | 5106 | + |
| | 1128 | 9 | *pU-21T* | | M Hetero | 6 | 14 | 149.83 | 3.68 | 112.17 | 10.03 | 6.28 | 2.40 | 358.83 | 4.53 | 10.81 | 19.03 | 21.89 | 23.85 | 497.55 | 0.4740 | 1173.08 | 0.5837 | 0.4971 | 217.64 | 5039.34 | 0.9747 | 72.3970 | 2.2668 | 0.0509 | 17.598 | 8.668 | 0.9190 | 0.00426 | 5462 | |
| | | B6;CB-*Nedd4Gt(pU-21T)269Imeg* | | | F WT | 6 | 14-16 | 150.67 | 3.67 | 115.83 | 9.52 | 5.80 | 2.28 | 357.17 | 6.92 | 14.16 | 19.27 | 20.89 | 21.88 | 429.45 | 0.1517 | 1202.60 | 0.5522 | 0.4591 | 211.84 | 4801.30 | 0.3461 | 61.4947 | 1.1197 | 0.1738 | 15.888 | 8.668 | 0.8707 | 0.00379 | 6996 | |
| | | | | | F Hetero | 2 | 15-16 | 150.33 | 3.55 | 113.83 | 9.58 | 6.02 | 2.42 | 409.17 | 5.15 | 10.02 | 16.59 | 17.89 | 19.68 | 425.25 | 0.1300 | 1188.22 | 0.4808 | 0.4043 | 197.23 | 4503.17 | 0.3022 | 60.3186 | 1.2056 | 0.1555 | 13.209 | 8.150 | 0.9057 | 0.00325 | 4161 | |
| | | | | 【SD】 | M WT | | | 2.07 | 0.43 | 2.25 | 0.24 | 2.67 | 0.28 | 23.21 | 1.05 | 3.82 | 3.07 | 2.33 | 1.82 | 14.52 | 0.1034 | 23.79 | 0.0349 | 0.0254 | 10.07 | 199.24 | 0.1900 | 3.0439 | 0.2967 | 0.0426 | 1.955 | 1.049 | 0.1954 | 0.00139 | 1028 | |
| | | | | | M Hetero | | | 1.33 | 0.53 | 1.72 | 0.30 | 0.66 | 0.18 | 63.70 | 0.62 | 1.80 | 1.61 | 1.69 | 1.30 | 11.25 | 0.1055 | 17.05 | 0.0604 | 0.0446 | 10.64 | 248.86 | 0.2203 | 3.0784 | 0.2689 | 0.0117 | 2.635 | 0.681 | 0.1311 | 0.00061 | 1769 | |
| | | | | | F WT | | | 1.21 | 0.49 | 2.40 | 0.20 | 1.18 | 0.25 | 46.46 | 1.97 | 2.05 | 1.33 | 1.27 | 1.26 | 29.15 | 0.0722 | 11.02 | 0.0320 | 0.0251 | 5.40 | 182.70 | 0.1434 | 5.9884 | 0.2996 | 0.0733 | 1.857 | 1.014 | 0.2378 | 0.00150 | 3138 | |
| | | | | | F Hetero | | | 1.37 | 0.52 | 1.17 | 0.27 | 1.68 | 0.26 | 44.10 | 1.58 | 2.45 | 1.74 | 1.23 | 2.00 | 26.55 | 0.0377 | 22.81 | 0.0455 | 0.0339 | 11.02 | 212.83 | 0.0757 | 3.6815 | 0.2005 | 0.0524 | 2.056 | 0.766 | 0.2074 | 0.00094 | 782 | |
| 19 | 21-T278 | *Tmem161a* | *KTPU8* | 【Mean】 | M WT | 3 | 10-12 | 149.67 | 4.07 | 113.00 | 10.11 | 6.30 | 2.33 | 467.67 | 5.71 | 13.38 | 22.28 | 25.65 | 28.08 | 412.38 | 0.2795 | 1049.76 | 0.3922 | 0.3721 | 172.31 | 4587.86 | 0.6739 | 62.6059 | 1.8390 | 0.1440 | 9.420 | 6.072 | 2.4580 | 0.00240 | 22135 | + |
| | 1069 | 8 | *pU-21T* | | M Homo | 3 | 11 | 150.67 | 4.17 | 112.33 | 10.13 | 6.07 | 2.10 | 391.33 | 7.02 | 15.05 | 24.95 | 28.38 | 30.75 | 447.84 | 0.3668 | 1154.66 | 0.4794 | 0.4150 | 200.62 | 4544.58 | 0.8207 | 66.0379 | 2.2999 | 0.0610 | 15.222 | 9.116 | 5.0881 | 0.00296 | 26760 | |
| | | B6;CB-*Tmem161aGt(pU-21T)278Imeg* | | | F WT | 3 | 11-12 | 150.00 | 3.73 | 114.33 | 10.13 | 5.80 | 2.20 | 489.00 | 5.17 | 13.24 | 18.67 | 19.85 | 21.55 | 375.50 | 0.1272 | 1091.06 | 0.3745 | 0.3433 | 175.78 | 4233.93 | 0.3335 | 58.7096 | 1.3503 | 0.1845 | 10.991 | 6.756 | 1.5516 | 0.00190 | 22850 | |
| | | | | | F Homo | 3 | 11-12 | 151.67 | 4.27 | 116.00 | 10.10 | 5.37 | 2.07 | 468.00 | 5.76 | 13.43 | 17.95 | 20.78 | 20.25 | 407.00 | 0.1234 | 1152.82 | 0.4179 | 0.3625 | 182.14 | 4316.99 | 0.3025 | 57.8273 | 1.2241 | 0.2277 | 11.944 | 8.165 | 5.5125 | 0.00237 | 23470 | |
| | | | | 【SD】 | M WT | | | 2.08 | 0.57 | 1.00 | 0.10 | 1.13 | 0.31 | 95.55 | 0.62 | 2.74 | 2.00 | 1.33 | 1.93 | 54.41 | 0.2410 | 42.08 | 0.1097 | 0.0767 | 24.18 | 308.50 | 0.4182 | 6.5919 | 0.3932 | 0.0459 | 1.888 | 1.075 | 3.3420 | 0.00070 | 6873 | |
| | | | | | M Homo | | | 0.58 | 0.57 | 2.52 | 0.45 | 0.74 | 0.10 | 109.20 | 0.81 | 2.46 | 1.19 | 0.61 | 1.08 | 31.62 | 0.1810 | 27.66 | 0.1052 | 0.0753 | 23.42 | 236.04 | 0.3279 | 2.6773 | 0.4465 | 0.0326 | 2.476 | 0.801 | 0.5672 | 0.00051 | 3618 | |
| | | | | | F WT | | | 1.00 | 0.23 | 2.08 | 0.06 | 0.44 | 0.00 | 42.51 | 0.99 | 1.64 | 1.02 | 1.11 | 1.47 | 26.07 | 0.0524 | 9.50 | 0.0158 | 0.0108 | 2.57 | 111.69 | 0.1077 | 2.2922 | 0.3124 | 0.0995 | 3.475 | 1.703 | 1.7662 | 0.00025 | 1699 | |
| | | | | | F Homo | | | 2.08 | 0.06 | 1.00 | 0.26 | 1.05 | 0.06 | 120.65 | 2.28 | 1.71 | 1.48 | 1.17 | 1.62 | 6.49 | 0.0195 | 45.96 | 0.8811 | 0.0299 | 8.44 | 212.80 | 0.0478 | 1.6381 | 0.2664 | 0.0418 | 2.364 | 1.321 | 0.3285 | 0.00060 | 1273 | |
| 20 | 21-T297 | *Mark3* | *KTPU8* | 【Mean】 | M WT | 3 | 15-16 | 149.25 | 4.20 | 110.25 | 9.70 | 5.90 | 2.25 | 320.50 | 8.49 | 16.71 | 25.69 | 30.34 | 33.24 | 498.62 | 0.4781 | 1197.50 | 0.6598 | 0.5513 | 223.78 | 5417.85 | 0.9598 | 74.6988 | 1.8598 | 0.0803 | 18.588 | 8.734 | 0.4780 | 0.00451 | 13439 | + |
| | 1077 | 12 | *pU-21T* | | M Hetero | 3 | 15 | 152.25 | 4.13 | 115.75 | 9.65 | 6.95 | 2.30 | 335.50 | 7.63 | 13.84 | 22.76 | 25.01 | 27.63 | 490.26 | 0.3647 | 1190.40 | 0.5930 | 0.4970 | 203.10 | 5260.49 | 0.7260 | 73.7473 | 1.4819 | 0.1498 | 16.848 | 7.608 | 0.5012 | 0.00312 | 11524 | |
| | | B6;CB-*Mark3Gt(pU-21T)297Imeg* | | | F WT | 3 | 15 | 152.00 | 4.27 | 116.67 | 9.43 | 6.93 | 2.13 | 379.33 | 7.26 | 13.02 | 18.57 | 20.01 | 20.95 | 398.33 | 0.0792 | 1219.20 | 0.5458 | 0.4476 | 211.21 | 4693.90 | 0.1875 | 57.7341 | 0.7665 | 0.3295 | 14.197 | 8.145 | 0.3852 | 0.00224 | 10740 | |
| | | | | | F Hetero | 3 | 15-16 | 151.00 | 4.30 | 116.33 | 9.33 | 6.83 | 2.17 | 402.33 | 6.72 | 11.14 | 18.17 | 19.02 | 19.56 | 382.13 | 0.0298 | 1230.06 | 0.5241 | 0.4255 | 200.79 | 4653.11 | 0.0770 | 53.1309 | 0.3681 | 0.5019 | 13.582 | 7.645 | 0.4325 | 0.00238 | 9281 | |
| | | | | 【SD】 | M WT | | | 0.50 | 0.45 | 4.19 | 0.22 | 0.94 | 0.06 | 76.04 | 1.61 | 3.27 | 1.32 | 2.28 | 4.68 | 39.99 | 0.2266 | 10.93 | 0.0537 | 0.0497 | 6.92 | 423.41 | 0.4006 | 6.3821 | 0.2352 | 0.0234 | 2.495 | 0.786 | 0.1688 | 0.00076 | 3210 | |
| | | | | | M Hetero | | | 3.30 | 0.69 | 4.19 | 0.29 | 2.95 | 0.45 | 31.31 | 1.15 | 2.78 | 1.77 | 1.83 | 2.70 | 69.45 | 0.2141 | 29.71 | 0.0956 | 0.0708 | 17.75 | 383.18 | 0.3692 | 10.6665 | 0.4010 | 0.0501 | 3.954 | 0.668 | 0.0949 | 0.00095 | 311 | |
| | | | | | F WT | | | 1.00 | 0.12 | 1.53 | 0.21 | 1.55 | 0.12 | 27.15 | 0.76 | 0.12 | 0.78 | 1.31 | 2.09 | 40.39 | 0.0523 | 9.06 | 0.0270 | 0.0201 | 11.95 | 29.13 | 0.1119 | 5.3708 | 0.3403 | 0.1760 | 0.599 | 0.314 | 0.1876 | 0.00032 | 354 | |
| | | | | | F Hetero | | | 1.73 | 0.44 | 2.31 | 0.12 | 0.81 | 0.21 | 42.34 | 0.32 | 0.67 | 0.45 | 0.87 | 0.80 | 51.26 | 0.0169 | 43.02 | 0.0478 | 0.0259 | 15.09 | 151.90 | 0.0473 | 4.6692 | 0.2306 | 0.1397 | 0.955 | 0.252 | 0.0277 | 0.00038 | 2488 | |
| 21 | 21-T322 | *Cyb5b* | *KTPU8* | 【Mean】 | M WT | 3 | 14 | 155.00 | 4.70 | 116.00 | 9.87 | 8.20 | 3.10 | 322.67 | 4.83 | 8.10 | 17.87 | 22.31 | 23.86 | 466.20 | 0.4763 | 1192.87 | 0.6023 | 0.5042 | 220.51 | 5047.09 | 1.0234 | 70.8510 | 2.3423 | 0.0441 | 18.958 | 9.242 | 0.3898 | 0.00359 | 12660 | − |
| | 1097 | 8 | *pU-21T* | | M Hetero | 3 | 14 | 153.00 | 4.27 | 116.67 | 9.77 | 6.93 | 2.93 | 355.00 | 5.28 | 7.64 | 17.38 | 22.46 | 24.08 | 485.87 | 0.4873 | 1194.30 | 0.5930 | 0.4964 | 217.29 | 5045.39 | 1.0257 | 72.9678 | 2.3922 | 0.0365 | 18.623 | 9.209 | 0.3844 | 0.00360 | 12316 | |
| | | B6;CB-*Cyb5bGt(pU-21T)322Imeg* | | | F WT | 3 | 14-15 | 154.33 | 3.40 | 115.67 | 9.93 | 8.23 | 2.67 | 423.00 | 5.21 | 8.87 | 16.13 | 17.47 | 18.98 | 382.67 | 0.0710 | 1207.00 | 0.4963 | 0.4112 | 204.46 | 4476.09 | 0.1808 | 56.4277 | 0.8590 | 0.2705 | 13.957 | 8.762 | 0.3187 | 0.00218 | 10337 | |
| | | | | | F Hetero | 3 | 14 | 154.33 | 3.53 | 116.00 | 9.93 | 8.37 | 2.67 | 362.33 | 5.19 | 7.85 | 17.35 | 18.42 | 19.71 | 396.57 | 0.1034 | 1200.17 | 0.4913 | 0.4095 | 200.58 | 4508.31 | 0.2522 | 58.7853 | 1.0294 | 0.2187 | 14.240 | 8.843 | 0.3464 | 0.00232 | 11519 | |
| | | | | 【SD】 | M WT | | | 1.73 | 0.26 | 0.00 | 0.23 | 1.01 | 0.17 | 27.02 | 0.53 | 2.33 | 2.06 | 1.38 | 1.65 | 34.22 | 0.2037 | 15.09 | 0.0983 | 0.0764 | 20.21 | 349.92 | 0.3868 | 8.5861 | 0.3631 | 0.0214 | 4.249 | 0.901 | 0.0589 | 0.00099 | 3643 | |
| | | | | | M Hetero | | | 2.65 | 0.58 | 1.53 | 0.25 | 0.50 | 0.29 | 38.12 | 0.27 | 2.49 | 1.46 | 2.00 | 2.44 | 43.11 | 0.0696 | 24.23 | 0.0518 | 0.0349 | 17.00 | 112.38 | 0.1085 | 6.3154 | 0.2765 | 0.0115 | 4.898 | 2.535 | 0.0521 | 0.00123 | 4890 | |
| | | | | | F WT | | | 4.04 | 0.40 | 4.04 | 0.15 | 0.91 | 0.25 | 35.03 | 0.83 | 2.95 | 1.21 | 1.22 | 1.21 | 6.70 | 0.0161 | 22.04 | 0.0237 | 0.0140 | 7.56 | 112.59 | 0.0389 | 1.8950 | 0.0958 | 0.0769 | 1.088 | 0.779 | 0.0359 | 0.00052 | 1983 | |
| | | | | | F Hetero | | | 2.89 | 0.21 | 2.65 | 0.67 | 1.42 | 0.45 | 93.29 | 0.62 | 1.10 | 1.28 | 0.93 | 0.75 | 48.75 | 0.0489 | 17.32 | 0.0162 | 0.0110 | 4.06 | 100.64 | 0.1069 | 3.0860 | 0.1880 | 0.0724 | 1.786 | 1.496 | 0.0721 | 0.00015 | 1902 | |
| 22 | 21-T34 | *EST* | *KTPU8* | 【Mean】 | M WT | 3 | 16 | 150.67 | 5.50 | 111.33 | 9.20 | 7.87 | 2.37 | 301.00 | 7.69 | 17.25 | 21.22 | 23.52 | 25.18 | 404.37 | 0.2553 | 1124.40 | 0.5010 | 0.4456 | 204.60 | 4776.75 | 0.6255 | 84.0113 | 1.2323 | 0.1502 | 16.442 | 197.300 | 0.7299 | 0.00361 | 9015 | − |
| | 1063 | 2 | *pU-21T* | | M Hom | 3 | 15 | 153.67 | 4.53 | 116.33 | 8.70 | 6.77 | 2.63 | 345.67 | 5.32 | 10.66 | 19.20 | 20.80 | 22.97 | 372.47 | 0.1684 | 1118.50 | 0.4806 | 0.4296 | 204.42 | 4631.43 | 0.4364 | 78.9465 | 1.0721 | 0.1803 | 15.784 | 189.410 | 0.7961 | 0.00360 | 10212 | |
| | | B6,Cg-*Gt(pU-21T)34Imeg* | | | F WT | 3 | 16 | 149.67 | 4.57 | 112.67 | 9.20 | 7.70 | 2.23 | 440.00 | 7.09 | 13.67 | 17.88 | 19.67 | 20.52 | 329.67 | 0.0617 | 1135.90 | 0.4794 | 0.4217 | 209.59 | 4486.04 | 0.1809 | 68.4376 | 0.5721 | 0.3565 | 14.458 | 173.500 | 0.5484 | 0.00263 | 10259 | |
| | | | | | F Hom | 3 | 15 | 150.00 | 4.10 | 114.33 | 8.17 | 7.23 | 2.47 | 492.00 | 5.75 | 11.66 | 15.95 | 18.19 | 19.47 | 338.80 | 0.0767 | 1133.00 | 0.4626 | 0.4083 | 210.99 | 4348.67 | 0.2174 | 68.0186 | 0.7918 | 0.2611 | 15.495 | 185.940 | 0.4987 | 0.00252 | 11442 | |
| | | | | 【SD】 | M WT | | | 0.58 | 0.46 | 0.58 | 0.17 | 0.42 | 0.23 | 12.53 | 0.36 | 1.17 | 2.69 | 1.29 | 2.02 | 39.31 | 0.0541 | 2.61 | 0.0268 | 0.0243 | 2.64 | 187.65 | 0.0870 | 11.8147 | 0.1297 | 0.0483 | 3.151 | 37.810 | 0.0860 | 0.00019 | 5783 | |
| | | | | | M Hom | | | 0.58 | 0.32 | 0.58 | 0.20 | 0.96 | 0.12 | 31.82 | 1.18 | 2.03 | 0.92 | 0.93 | 0.69 | 37.97 | 0.0806 | 8.53 | 0.0343 | 0.0284 | 6.78 | 170.26 | 0.1664 | 7.1046 | 0.1781 | 0.0179 | 4.708 | 56.490 | 0.2456 | 0.00071 | 6462 | |
| | | | | | F WT | | | 0.58 | 0.32 | 1.53 | 0.30 | 0.40 | 0.06 | 98.77 | 1.11 | 1.12 | 0.70 | 1.10 | 0.99 | 10.13 | 0.0087 | 31.16 | 0.0352 | 0.0200 | 12.50 | 58.68 | 0.0238 | 3.4089 | 0.1038 | 0.0535 | 2.506 | 30.072 | 0.1251 | 0.00012 | 4306 | |
| | | | | | F Hom | | | 1.00 | 0.17 | 3.21 | 0.32 | 0.25 | 0.15 | 8.54 | 1.18 | 2.11 | 1.11 | 0.90 | 1.56 | 16.82 | 0.0321 | 5.00 | 0.0144 | 0.0115 | 7.39 | 93.47 | 0.0826 | 2.8027 | 0.1700 | 0.0672 | 2.073 | 24.875 | 0.1723 | 0.00036 | 2657 | |
| 23 | 21-T354 | *Pkig* | *KTPU8* | 【Mean】 | M WT | 3 | 12 | 150.33 | 4.27 | 111.67 | 9.40 | 6.07 | 2.77 | 318.33 | 7.31 | 13.04 | 22.99 | 26.01 | 28.00 | 473.27 | 0.4899 | 1143.67 | 0.5905 | 0.5162 | 223.35 | 5112.50 | 1.0558 | 70.7185 | 2.4726 | 0.0403 | 17.645 | 9.606 | 0.4537 | 0.00437 | 12709 | + |
| | 1095 | 2 | *pU-21T* | | M Homo | 3 | 12 | 150.33 | 4.77 | 113.33 | 9.43 | 5.53 | 2.47 | 276.67 | 7.16 | 12.48 | 23.49 | 27.37 | 29.24 | 489.67 | 0.7070 | 1150.17 | 0.6548 | 0.5690 | 229.80 | 5451.39 | 1.4783 | 73.9256 | 2.7739 | 0.0357 | 20.182 | 8.455 | 0.6357 | 0.00533 | 11439 | |
| | | B6;CB-*PkigGt(pU-21T)354Imeg* | | | F WT | 3 | 12 | 149.67 | 4.13 | 113.00 | 9.13 | 6.47 | 2.50 | 364.00 | 5.71 | 11.81 | 18.32 | 19.89 | 20.79 | 344.77 | 0.0646 | 1149.43 | 0.4928 | 0.4286 | 199.66 | 4702.27 | 0.1793 | 49.7064 | 0.8593 | 0.2617 | 12.385 | 6.463 | 0.5063 | 0.00249 | 7045 | |
| | | | | | F Homo | 3 | 12 | 150.33 | 3.63 | 115.00 | 9.47 | 6.74 | 2.30 | 439.67 | 6.74 | 11.69 | 18.69 | 20.44 | 21.30 | 437.91 | 0.3555 | 1152.97 | 0.5645 | 0.4896 | 217.99 | 4955.39 | 0.7661 | 64.9235 | 1.8378 | 0.1131 | 14.623 | 8.488 | 0.3831 | 0.00221 | 11229 | |
| | | | | 【SD】 | M WT | | | 0.58 | 0.40 | 2.08 | 0.20 | 0.50 | 0.23 | 18.18 | 0.49 | 1.71 | 0.46 | 0.25 | 0.23 | 10.19 | 0.0053 | 8.84 | 0.0317 | 0.0237 | 7.36 | 97.58 | 0.0118 | 0.2768 | 0.0595 | 0.0027 | 1.955 | 2.602 | 0.0014 | 0.00042 | 1398 | |
| | | | | | M Homo | | | 0.58 | 1.15 | 2.31 | 0.51 | 0.96 | 0.40 | 45.08 | 0.18 | 1.71 | 1.29 | 0.28 | 0.57 | 12.30 | 0.1091 | 20.19 | 0.0488 | 0.0327 | 12.42 | 131.46 | 0.2315 | 1.7092 | 0.3711 | 0.0174 | 0.845 | 0.607 | 0.0514 | 0.00060 | 1171 | |
| | | | | | F WT | | | 1.53 | 0.45 | 2.00 | 0.12 | 0.87 | 0.10 | 35.00 | 0.23 | 0.17 | 0.21 | 0.20 | 0.14 | 11.97 | 0.0169 | 11.71 | 0.0309 | 0.0226 | 8.04 | 88.90 | 0.0437 | 3.0496 | 0.1520 | 0.0546 | 1.946 | 1.099 | 0.1017 | 0.00036 | 3500 | |
| | | | | | F Homo | | | 1.15 | 0.23 | 5.29 | 0.12 | 0.44 | 0.44 | 118.69 | 0.57 | 1.08 | 0.62 | 0.70 | 0.67 | 57.90 | 0.2707 | 16.95 | 0.0777 | 0.0672 | 16.09 | 371.60 | 0.5424 | 9.9719 | 0.7903 | 0.0912 | 1.454 | 0.850 | 0.1037 | 0.00069 | 589 | |
| 24 | 21-T54 | *Brd1* | *KTPU8* | 【Mean】 | M WT | 5 | 14-15 | 150.40 | 4.68 | 112.80 | 9.53 | 5.68 | 2.34 | 305.40 | 8.33 | 21.99 | 27.87 | 31.84 | 34.59 | 495.66 | 0.3912 | 1235.96 | 0.7202 | 0.5828 | 251.95 | 5257.60 | 0.7983 | 71.9899 | 1.7729 | 0.0675 | 17.861 | 7.987 | 1.0147 | 0.00456 | 4502 | + |
| | 1807 | 15 | *pU-21T* | | M Hetero | 5 | 13-15 | 151.00 | 5.32 | 114.40 | 9.78 | 7.90 | 2.46 | 323.60 | 7.91 | 18.03 | 26.65 | 30.34 | 32.34 | 499.18 | 0.4148 | 1225.02 | 0.6655 | 0.5435 | 242.58 | 5074.47 | 0.8395 | 75.4500 | 1.8867 | 0.0621 | 21.133 | 10.459 | 1.1628 | 0.00544 | 5040 | |
| | | B6;CB-*Brd1Gt(pU-21T)54Card* | | | F WT | 5 | 15 | 150.20 | 3.94 | 113.00 | 9.54 | 4.80 | 2.26 | 357.80 | 8.71 | 19.51 | 23.14 | 25.75 | 27.87 | 484.94 | 0.1690 | 1265.62 | 0.6800 | 0.5371 | 254.46 | 4874.59 | 0.3484 | 68.6184 | 1.0297 | 0.1662 | 20.214 | 10.711 | 1.2835 | 0.00484 | 4664 | |
| | | | | | F Hetero | 5 | 15 | 151.80 | 4.52 | 114.80 | 9.36 | 5.62 | 2.28 | 424.00 | 7.41 | 15.76 | 20.80 | 21.50 | 22.18 | 439.34 | 0.1199 | 1239.42 | 0.5611 | 0.4526 | 228.12 | 4528.37 | 0.2660 | 63.3895 | 1.0110 | 0.1712 | 18.414 | 11.340 | 0.9819 | 0.00440 | 5070 | |
| | | | | 【SD】 | M WT | | | 1.52 | 0.53 | 2.17 | 0.10 | 0.42 | 0.24 | 25.40 | 0.20 | 1.09 | 0.99 | 1.08 | 2.02 | 34.70 | 0.0644 | 5.42 | 0.0329 | 0.0290 | 6.66 | 140.44 | 0.0834 | 5.6024 | 0.0926 | 0.0130 | 8.676 | 3.840 | 0.4063 | 0.00250 | 1234 | |
| | | | | | M Hetero | | | 1.73 | 0.90 | 2.61 | 0.39 | 1.12 | 0.05 | 42.60 | 0.70 | 2.58 | 1.94 | 2.52 | 2.93 | 36.54 | 0.1039 | 14.28 | 0.0506 | 0.0435 | 8.51 | 267.71 | 0.1804 | 6.8651 | 0.2343 | 0.0213 | 2.973 | 2.215 | 0.2883 | 0.00091 | 1165 | |
| | | | | | F WT | | | 1.30 | 0.53 | 1.00 | 0.27 | 0.68 | 0.13 | 27.38 | 0.50 | 0.90 | 0.85 | 0.57 | 0.98 | 43.81 | 0.0438 | 14.18 | 0.0421 | 0.0272 | 12.53 | 87.79 | 0.0648 | 5.0863 | 0.0883 | 0.0288 | 3.093 | 1.760 | 0.2948 | 0.00066 | 467 | |
| | | | | | F Hetero | | | 2.28 | 0.38 | 2.49 | 0.18 | 0.90 | 0.31 | 67.52 | 0.76 | 1.53 | 0.41 | 0.51 | 0.61 | 38.19 | 0.0532 | 13.51 | 0.0341 | 0.0240 | 14.83 | 119.08 | 0.1035 | 6.2598 | 0.2343 | 0.0587 | 1.694 | 1.562 | 0.2293 | 0.00055 | 1482 | |
| 25 | 21-T57 | *Fbxo17* | *KTPU8* | 【Mean】 | M WT | 3 | 15 | 151.00 | 3.67 | 112.33 | 10.07 | 5.17 | 2.57 | 316.67 | 6.81 | 16.24 | 21.54 | ND | 27.42 | 497.80 | 0.5460 | 1193.07 | 0.6287 | 0.5244 | 218.09 | 5225.38 | 1.0920 | 75.9773 | 2.0070 | 0.0672 | 19.465 | 233.580 | 0.3281 | 0.00309 | 13326 | − |
| | 839 | 7 | *pU-21T* | | M Homo | 3 | 15 | 150.00 | 4.07 | 110.67 | 9.93 | 5.60 | 2.57 | 310.00 | 6.38 | 16.95 | 22.31 | ND | 26.58 | 524.77 | 0.6292 | 1211.17 | 0.6507 | 0.5374 | 229.39 | 5199.50 | 1.2238 | 78.7320 | 2.2046 | 0.0472 | 21.188 | 254.260 | 0.4157 | 0.00476 | 17217 | |
| | | B6;CB-*Fbxo17Gt(pU-21T)57Imeg* | | | F WT | 3 | 15 | 150.33 | 3.27 | 115.00 | 9.57 | 5.10 | 2.53 | 436.33 | 7.73 | 18.57 | 19.54 | ND | 21.79 | 410.33 | 0.1388 | 1216.07 | 0.5615 | 0.4616 | 218.62 | 4707.28 | 0.3330 | 61.6323 | 1.2147 | 0.1456 | 17.947 | 215.360 | 0.3360 | 0.00305 | 14523 | |
| | | | | | F Homo | 3 | 14-15 | 149.00 | 3.17 | 112.00 | 9.70 | 5.70 | 2.47 | 371.33 | 7.61 | 17.83 | 19.43 | ND | 22.46 | 434.80 | 0.1519 | 1214.27 | 0.5466 | 0.4501 | 215.42 | 4663.22 | 0.3505 | 62.3181 | 1.2644 | 0.1345 | 16.635 | 199.620 | 0.3288 | 0.00270 | 12848 | |
| | | | | 【SD】 | M WT | | | 1.73 | 0.40 | 3.06 | 0.32 | 0.21 | 0.38 | 46.65 | 1.52 | 6.12 | 3.98 | ND | 3.23 | 64.88 | 0.2011 | 44.80 | 0.1460 | 0.1040 | 25.14 | 510.11 | 0.2867 | 7.8031 | 0.1213 | 0.0223 | 4.663 | 55.958 | 0.0526 | 0.00081 | 5069 | |
| | | | | | M Homo | | | 1.00 | 0.29 | 2.52 | 0.15 | 0.35 | 0.29 | 24.00 | 0.43 | 2.02 | 0.47 | ND | 0.79 | 10.44 | 0.2128 | 18.76 | 0.0362 | 0.0337 | 6.21 | 262.39 | 0.4074 | 1.7646 | 0.4291 | 0.0315 | 1.711 | 20.532 | 0.0619 | 0.00079 | 345 | |
| | | | | | F WT | | | 1.15 | 0.15 | 0.00 | 0.12 | 0.70 | 0.25 | 19.35 | 0.70 | 0.28 | 0.22 | ND | 1.04 | 26.98 | 0.0449 | 11.73 | 0.0252 | 0.0170 | 11.23 | 11.84 | 0.0875 | 3.2721 | 0.2172 | 0.0372 | 0.522 | 6.266 | 0.0453 | 0.00035 | 1101 | |
| | | | | | F Homo | | | 2.00 | 0.64 | 3.00 | 0.17 | 0.82 | 0.12 | 40.87 | 0.83 | 1.31 | 1.02 | ND | 2.60 | 16.47 | 0.0142 | 17.64 | 0.0299 | 0.0214 | 11.05 | 80.13 | 0.0314 | 1.9782 | 0.0482 | 0.0192 | 0.687 | 8.249 | 0.0217 | 0.00038 | 1576 | |
| 26 | 21-T75 | *4933407K13Rik; uncRNA* | *KTPU8* | 【Mean】 | M WT | 3 | 14 | 151.00 | 4.37 | 110.33 | 8.73 | 7.87 | 2.70 | 247.00 | 7.25 | 17.31 | 24.21 | 25.88 | 28.39 | 479.43 | 0.3269 | 1166.60 | 0.5996 | 0.5127 | 227.16 | 5012.37 | 0.6613 | 69.7350 | 1.5156 | 0.1341 | 18.937 | 9.276 | 0.6606 | 0.00409 | 9529 | - |
| | 1042 | X | *pU-21T* | | M Hetero | 3 | 14 | 151.67 | 4.57 | 111.33 | 8.73 | 9.33 | 2.73 | 243.67 | 7.69 | 17.59 | 24.51 | 26.60 | 29.47 | 449.70 | 0.3287 | 1152.83 | 0.5810 | 0.5037 | 216.45 | 5094.07 | 0.7073 | 67.7887 | 1.5943 | 0.1201 | 17.580 | 8.493 | 0.6256 | 0.00361 | 7476 | |
| | | B6;CB-*4933407K13RikGt(pU-21T)75Imeg* | | | F Hetero | 3 | 14 | 150.00 | 4.17 | 112.33 | 8.83 | 9.23 | 2.53 | 440.00 | 7.05 | 14.38 | 18.76 | 20.28 | 21.53 | 361.13 | 0.0455 | 1173.50 | 0.4989 | 0.4251 | 207.45 | 4537.97 | 0.1354 | 54.3989 | 0.6714 | 0.3844 | 15.092 | 9.242 | 0.6356 | 0.00274 | 9519 | |
| | | | | | F Hom | 3 | 14 | 150.33 | 4.53 | 114.00 | 8.97 | 8.47 | 2.33 | 470.00 | 6.42 | 13.66 | 18.37 | 20.20 | 21.07 | 379.97 | 0.0833 | 1180.03 | 0.5042 | 0.4272 | 209.02 | 4545.16 | 0.2416 | 56.5177 | 0.8616 | 0.2315 | 15.740 | 9.571 | 0.6467 | 0.00296 | 9584 | |
| | | | | 【SD】 | M WT | | | 1.73 | 0.12 | 0.58 | 0.25 | 0.15 | 0.10 | 20.81 | 0.69 | 0.96 | 1.37 | 2.06 | 2.27 | 75.39 | 0.1722 | 27.39 | 0.1105 | 0.0839 | 28.03 | 328.98 | 0.2949 | 10.2743 | 0.2556 | 0.0548 | 6.519 | 2.232 | 0.2674 | 0.00189 | 8126 | |
| | | | | | M Hetero | | | 0.58 | 0.21 | 0.58 | 0.31 | 0.68 | 0.06 | 23.80 | 0.52 | 0.36 | 1.18 | 2.54 | 2.50 | 60.21 | 0.1852 | 25.41 | 0.0730 | 0.0598 | 14.77 | 426.29 | 0.3173 | 8.9257 | 0.1796 | 0.0134 | 2.743 | 0.569 | 0.0325 | 0.00036 | 301 | |
| | | | | | F Hetero | | | 2.00 | 0.23 | 0.58 | 0.12 | 0.47 | 0.06 | 10.58 | 0.54 | 1.14 | 1.49 | 1.16 | 1.90 | 16.73 | 0.0076 | 9.99 | 0.0367 | 0.0299 | 8.39 | 158.27 | 0.0203 | 0.9805 | 0.1252 | 0.0943 | 1.275 | 1.114 | 0.3898 | 0.00067 | 4517 | |
| | | | | | F Hom | | | 0.58 | 0.15 | 0.00 | 0.12 | 1.01 | 0.31 | 30.05 | 1.80 | 2.71 | 2.60 | 2.85 | 3.02 | 13.24 | 0.0214 | 2.54 | 0.0465 | 0.0386 | 6.77 | 274.67 | 0.0034 | 0.3842 | 0.1072 | 0.0531 | 1.756 | 0.592 | 0.2120 | 0.00037 | 3754 | |
| 27 | 21-W105 | *Epb4.1l2* | *KTPU8* | 【Mean】 | M WT | 3 | 15-16 | 148.67 | 4.33 | 112.00 | 9.50 | 6.50 | 2.80 | 263.00 | 6.85 | 15.89 | 23.08 | 25.61 | 27.35 | 489.87 | 0.6072 | 1182.00 | 0.6710 | 0.5673 | 224.79 | 5516.85 | 1.2421 | 72.0706 | 2.2754 | 0.0613 | 22.348 | 9.230 | 0.5421 | 0.00496 | 14241 | + |
| | 1222 | 10 | *pU-21W* | | M Homo | 4 | 16 | 149.75 | 3.98 | 110.50 | 9.75 | 6.15 | 2.65 | 276.00 | 5.97 | 15.37 | 21.81 | 24.19 | 25.68 | 496.40 | 0.5905 | 1185.30 | 0.6384 | 0.5381 | 224.28 | 5290.16 | 1.1990 | 75.1504 | 2.3754 | 0.0417 | 18.018 | 8.037 | 0.6585 | 0.00393 | 7503 | |
| | | B6;CB-*Epb4.1l2Gt(pU-21W)105Card* | | | F WT | 4 | 16 | 149.25 | 3.80 | 115.75 | 9.45 | 5.93 | 2.63 | 405.00 | ND | 14.62 | 17.77 | 18.87 | 19.60 | 369.78 | 0.0870 | 1175.43 | 0.4978 | 0.4235 | 206.94 | 4524.40 | 0.2281 | 54.1644 | 0.9798 | 0.2227 | 16.326 | 10.048 | 0.4082 | 0.00239 | 12183 | |
| | | | | | F Homo | 3 | 15 | 151.67 | 4.20 | 116.33 | 9.53 | 9.10 | 2.27 | 502.67 | 4.50 | 10.57 | 16.14 | 17.48 | 18.23 | 433.53 | 0.1455 | 1171.07 | 0.4968 | 0.4240 | 203.11 | 4588.05 | 0.3308 | 62.0725 | 1.1900 | 0.1450 | 14.072 | 8.482 | 0.4554 | 0.00242 | 10592 | |
| | | | | 【SD】 | M WT | | | 2.08 | 0.40 | 3.46 | 0.10 | 1.01 | 0.52 | z | 0.69 | 0.92 | 1.22 | 0.48 | 0.64 | 57.32 | 0.1953 | 33.63 | 0.0511 | 0.0313 | 12.59 | 51.66 | 0.2951 | 5.2572 | 0.3337 | 0.0285 | 1.408 | 0.639 | 0.0773 | 0.00113 | 1623 | |
| | | | | | M Homo | | | 0.96 | 0.30 | 0.58 | 0.29 | 0.41 | 0.29 | 40.26 | 0.18 | 0.88 | 1.05 | 1.17 | 1.05 | 48.95 | 0.1794 | 21.26 | 0.0612 | 0.0437 | 12.85 | 171.22 | 0.2840 | 8.1167 | 0.1523 | 0.0115 | 3.634 | 1.156 | 0.1122 | 0.00111 | 2421 | |
| | | | | | F WT | | | 0.50 | 0.29 | 1.71 | 0.25 | 0.43 | 0.26 | 30.25 | ND | 1.41 | 0.62 | 1.59 | 1.50 | 12.74 | 0.0245 | 9.26 | 0.0074 | 0.0041 | 2.66 | 75.49 | 0.0609 | 2.4706 | 0.1510 | 0.0476 | 2.715 | 1.878 | 0.1173 | 0.00032 | 1713 | |
| | | | | | F Homo | | | 1.15 | 0.26 | 2.08 | 0.15 | 0.79 | 0.12 | 45.00 | 0.35 | 1.24 | 1.49 | 1.05 | 1.87 | 33.12 | 0.0655 | 11.14 | 0.0431 | 0.0328 | 6.24 | 212.54 | 0.1366 | 6.2913 | 0.2411 | 0.0650 | 1.302 | 1.542 | 0.0218 | 0.00010 | 650 | |
| 28 | 21-W115 | *New* | *KTPU8* | 【Mean】 | M WT | 3 | 14-16 | 152.67 | 4.77 | 113.00 | 8.90 | 7.83 | 2.93 | 309.00 | 6.34 | 11.03 | 21.66 | 24.73 | 26.98 | 436.93 | 0.6680 | 1135.30 | 0.5770 | 0.5078 | 218.15 | 5116.53 | 1.4743 | 91.4703 | 2.0899 | 0.0662 | 19.716 | 9.187 | 0.7404 | 0.00408 | 14369 | + |
| | 1284 | 11 | *pU-21W* | | M Hom | 3 | 14-16 | 152.00 | 4.53 | 114.33 | 8.83 | 8.13 | 2.90 | 310.67 | 6.12 | 11.59 | 21.23 | 23.89 | 25.76 | 455.40 | 0.5383 | 1140.50 | 0.5937 | 0.5201 | 223.15 | 5140.22 | 1.1956 | 90.6423 | 1.8347 | 0.0951 | 19.557 | 9.209 | 0.7466 | 0.00434 | 10419 | |
| | | B6,Cg-*Gt(pU-21W)115Card* | | | F WT | 3 | 16 | 151.67 | 3.77 | 114.33 | 8.80 | 7.87 | 2.60 | 448.67 | 5.71 | 9.34 | 17.65 | 19.32 | 20.52 | 392.60 | 0.1333 | 1159.17 | 0.4913 | 0.4238 | 217.45 | 4394.03 | 0.3343 | 73.2880 | 0.8776 | 0.2752 | 13.282 | 8.765 | 0.7748 | 0.00227 | 13195 | |
| | | | | | F Hom | 3 | 14-16 | 150.67 | 4.07 | 114.33 | 8.90 | 8.63 | 2.57 | 440.33 | 6.08 | 11.36 | 17.70 | 19.47 | 21.15 | 348.93 | 0.0888 | 1164.47 | 0.5050 | 0.4338 | 216.86 | 4502.74 | 0.2487 | 67.9653 | 0.8533 | 0.2524 | 17.783 | 11.023 | 0.6410 | 0.00313 | 13098 | |
| | | | | 【SD】 | M WT | | | 1.53 | 0.81 | 1.73 | 0.10 | 0.76 | 0.06 | 45.74 | 0.61 | 1.16 | 0.57 | 0.94 | 1.76 | 69.50 | 0.4305 | 13.26 | 0.0919 | 0.0767 | 19.99 | 391.30 | 0.8616 | 14.3848 | 0.5742 | 0.0568 | 7.501 | 2.425 | 0.0873 | 0.00241 | 4458 | |
| | | | | | M Hom | | | 0.00 | 0.40 | 1.15 | 0.15 | 0.59 | 0.20 | 47.35 | 0.79 | 1.94 | 0.76 | 0.54 | 0.90 | 25.19 | 0.1470 | 18.73 | 0.0603 | 0.0482 | 14.49 | 191.07 | 0.2720 | 5.0858 | 0.2384 | 0.0237 | 5.397 | 2.007 | 0.2831 | 0.00107 | 6102 | |
| | | | | | F WT | | | 0.58 | 0.25 | 2.08 | 0.26 | 1.67 | 0.20 | 21.20 | 0.49 | 0.36 | 0.09 | 0.12 | 0.21 | 36.18 | 0.0356 | 12.08 | 0.0393 | 0.0330 | 9.55 | 164.07 | 0.0649 | 4.0161 | 0.0754 | 0.0225 | 4.038 | 3.091 | 0.1333 | 0.00055 | 1940 | |
| | | | | | F Hom | | | 0.58 | 0.42 | 1.15 | 0.10 | 0.61 | 0.25 | 24.58 | 0.85 | 2.60 | 1.66 | 2.00 | 2.27 | 20.36 | 0.0111 | 3.04 | 0.0121 | 0.0097 | 1.43 | 80.52 | 0.0277 | 1.3041 | 0.0712 | 0.0361 | 1.117 | 0.657 | 0.1165 | 0.00022 | 658 | |
| 29 | 21-W116 | *Cirh1a* | *KTPU8* | 【Mean】 | M WT | 6 | 15-16 | 151.33 | 4.27 | 113.67 | 9.53 | 6.82 | 2.52 | 244.67 | 7.50 | 16.12 | 23.39 | 26.53 | 28.73 | 559.92 | 0.6092 | 1230.62 | 0.7352 | 0.5971 | 245.07 | 5448.69 | 1.1194 | 81.7670 | 1.9972 | 0.0840 | 23.328 | 9.878 | 0.5993 | 0.00479 | 12065 | − |
| | 1263 | 8 | *pU-21W* | | M Hetero | 6 | 15-16 | 141.80 | 4.18 | 101.00 | 9.78 | 7.56 | 2.54 | 354.00 | 6.74 | 17.29 | 23.01 | 26.11 | 27.98 | 504.27 | 0.5868 | 1207.43 | 0.6673 | 0.5520 | 220.70 | 5447.29 | 1.1798 | 75.2748 | 2.1705 | 0.0639 | 21.648 | 9.147 | 0.5023 | 0.00437 | 14245 | |
| | | B6;CB-*Cirh1aGt(pU-21W)116Card* | | | F WT | 6 | 15-16 | 149.50 | 3.70 | 112.00 | 9.55 | 7.13 | 2.33 | 392.50 | 7.08 | 13.93 | 18.09 | 19.51 | 20.95 | 410.12 | 0.1319 | 1223.35 | 0.5464 | 0.4462 | 211.98 | 4663.17 | 0.3112 | 61.2157 | 1.1348 | 0.1896 | 15.949 | 9.196 | 0.3969 | 0.00252 | 12591 | |
| | | | | | F Hetero | 6 | 15 | 149.50 | 3.57 | 114.83 | 9.58 | 7.90 | 2.30 | 370.83 | 6.37 | 12.19 | 17.76 | 19.18 | 20.07 | 424.78 | 0.1327 | 1224.38 | 0.5361 | 0.4375 | 216.71 | 4544.55 | 0.3099 | 60.1843 | 1.1416 | 0.1587 | 15.169 | 9.238 | 0.4033 | 0.00241 | 11626 | |
| | | | | 【SD】 | M WT | | | 3.01 | 0.26 | 3.88 | 0.23 | 0.94 | 0.21 | 55.41 | 0.92 | 2.54 | 2.12 | 1.87 | 1.82 | 29.87 | 0.1803 | 21.15 | 0.0741 | 0.0557 | 17.22 | 270.83 | 0.3554 | 4.6545 | 0.3855 | 0.0519 | 2.518 | 0.889 | 0.1818 | 0.00181 | 4954 | |
| | | | | | M Hetero | | | 6.19 | 0.61 | 7.92 | 0.25 | 1.87 | 0.35 | 57.41 | 0.38 | 2.91 | 1.68 | 2.00 | 1.60 | 31.13 | 0.1454 | 21.16 | 0.0780 | 0.0568 | 14.85 | 244.58 | 0.2594 | 4.1366 | 0.3284 | 0.0316 | 2.132 | 0.254 | 0.0904 | 0.00140 | 1665 | |
| | | | | | F WT | | | 5.99 | 0.27 | 6.54 | 0.34 | 1.70 | 0.27 | 36.56 | 0.40 | 2.30 | 1.14 | 1.26 | 1.37 | 44.20 | 0.0545 | 26.60 | 0.0530 | 0.0357 | 12.70 | 150.30 | 0.1155 | 5.8245 | 0.2655 | 0.0990 | 1.589 | 0.423 | 0.0880 | 0.00039 | 1492 | |
| | | | | | F Hetero | | | 0.84 | 0.36 | 1.72 | 0.22 | 1.34 | 0.13 | 44.52 | 0.44 | 1.08 | 0.41 | 1.01 | 1.15 | 22.11 | 0.0265 | 28.23 | 0.0462 | 0.0297 | 16.78 | 135.56 | 0.0511 | 3.9164 | 0.0871 | 0.0334 | 1.193 | 0.731 | 0.1154 | 0.00023 | 1506 | |
| 30 | 21-W203 | *New* | *KTPU8* | 【Mean】 | M WT | 2 | 14-15 | 4.53 | 110.00 | 9.25 | 7.40 | 2.80 | 349.50 | ND | 7.00 | 12.22 | 10.82 | 24.49 | 26.19 | 341.25 | 0.1331 | 1118.20 | 0.4870 | 0.4350 | 203.11 | 4692.05 | 0.3705 | 66.6912 | 1.0886 | 0.1987 | 12.244 | 7.021 | 1.2034 | 0.00377 | 3497 | + |
| | 1621 | 3 | *pU-21W* | | M Hom | 3 | 15 | 4.80 | 113.33 | 8.80 | 7.73 | 2.80 | 383.33 | ND | ND | 9.34 | 19.34 | 23.12 | 24.52 | 434.87 | 0.3470 | 1120.27 | 0.5517 | 0.4925 | 210.21 | 5109.26 | 0.8017 | 88.4453 | 1.4067 | 0.1862 | 18.203 | 8.830 | 1.2053 | 0.00465 | 6771 | |
| | | B6;CB-*Gt(pU-21W)203Card* | | | F WT | 3 | 14-15 | 4.53 | 114.67 | 9.27 | 8.00 | 2.57 | 486.67 | ND | ND | 7.27 | 15.86 | 17.78 | 18.97 | 353.43 | 0.0622 | 1115.57 | 0.4423 | 0.3965 | 200.29 | 4385.65 | 0.1700 | 69.1475 | 0.6262 | 0.3737 | 14.443 | 9.454 | 1.1571 | 0.00371 | 4828 | |
| | | | | | F Hom | 4 | 14-15 | 4.95 | 115.00 | 9.00 | 8.98 | 2.83 | 564.75 | ND | 6.57 | 7.87 | 14.82 | 15.72 | 17.87 | 341.63 | 0.0466 | 1102.48 | 0.4008 | 0.3632 | 182.30 | 4323.25 | 0.1311 | 66.6090 | 0.5209 | 0.4416 | 11.092 | 7.436 | 0.9162 | 0.00326 | 4862 | |
| | | | | 【SD】 | M WT | | | 0.78 | 1.41 | 0.07 | 0.42 | 0.00 | 3.54 | ND | ND | 1.94 | 14.87 | ND | 0.15 | 41.22 | 0.0508 | 15.41 | 0.0354 | 0.0256 | 5.32 | 154.04 | 0.1056 | 8.2722 | 0.0185 | 0.0370 | 1.119 | 1.099 | 0.1119 | 0.00004 | 21 | |
| | | | | | M Hom | | | 0.26 | 0.58 | 0.20 | 0.67 | 0.00 | 58.35 | ND | ND | 1.38 | 1.26 | 0.20 | 0.15 | 9.09 | 0.0793 | 12.93 | 0.0133 | 0.0163 | 2.97 | 159.12 | 0.1769 | 3.7248 | 0.1958 | 0.0075 | 2.557 | 1.752 | 0.3440 | 0.00174 | 2299 | |
| | | | | | F WT | | | 0.61 | 1.53 | 0.55 | 0.70 | 0.40 | 53.80 | ND | ND | 0.89 | 0.95 | 1.09 | 0.32 | 26.13 | 0.0062 | 3.76 | 0.0067 | 0.0067 | 2.74 | 58.12 | 0.0156 | 5.6214 | 0.1731 | 0.0982 | 1.374 | 1.146 | 0.3553 | 0.00125 | 2322 | |
| | | | | | F Hom | | | 0.34 | 3.46 | 0.08 | 1.48 | 0.61 | 76.09 | ND | ND | 0.94 | 2.07 | 1.99 | 1.14 | 18.80 | 0.0214 | 22.41 | 0.0390 | 0.0291 | 9.46 | 162.12 | 0.0584 | 2.3777 | 0.1453 | 0.1102 | 2.073 | 1.159 | 0.2903 | 0.00115 | 1620 | |
| 31 | 21-W234 | *Etf1* | *KTPU8* | 【Mean】 | M WT | 3 | 14-16 | 152.00 | 5.03 | 112.67 | 9.37 | 10.23 | 2.73 | 320.67 | 6.06 | 15.20 | 22.42 | 23.59 | 26.84 | 497.07 | 0.3327 | 1193.23 | 0.6505 | 0.5448 | 240.13 | 5095.42 | 0.6715 | 74.7358 | 1.5194 | 0.1587 | 13.425 | 6.237 | 0.6880 | 0.00293 | 5507 | + |
| | 1817 | 18 | *pU-21W* | | M Hetero | 3 | 14 | 151.67 | 3.77 | 112.67 | 9.27 | 5.70 | 2.23 | 447.33 | 6.16 | 15.28 | 22.84 | 23.67 | 23.93 | 477.43 | 0.3606 | 1147.47 | 0.5249 | 0.4570 | 196.00 | 4999.81 | 0.7532 | 69.5403 | 1.6339 | 0.1402 | 15.386 | 7.587 | 1.0362 | 0.00466 | 3223 | |
| | | B6;CB-*Etf1Gt(pU-21W)234Card* | | | F WT | 3 | 14 | 150.33 | 3.97 | 114.33 | 9.17 | 7.37 | 2.30 | 463.33 | 6.99 | 15.08 | 18.80 | 20.65 | 22.16 | 429.97 | 0.0851 | 1171.33 | 0.5198 | 0.4450 | 215.55 | 4679.88 | 0.1958 | 61.9843 | 0.7726 | 0.2740 | 11.056 | 7.275 | 0.7924 | 0.00257 | 3969 | |
| | | | | | F Hetero | 3 | 14 | 150.00 | 3.70 | 114.00 | 9.17 | 7.70 | 2.27 | 573.33 | 6.31 | 13.91 | 18.14 | 20.43 | 21.12 | 421.67 | 0.0730 | 1174.43 | 0.4803 | 0.4090 | 202.81 | 4452.66 | 0.1698 | 61.4577 | 0.7465 | 0.2941 | 14.837 | 9.403 | 1.3251 | 0.00327 | 5562 | |
| | | | | 【SD】 | M WT | | | 1.00 | 0.47 | 1.15 | 0.21 | 2.48 | 0.15 | 23.29 | 0.11 | 2.09 | 1.64 | 0.89 | 2.48 | 32.27 | 0.1029 | 9.91 | 0.0860 | 0.0681 | 16.18 | 316.55 | 0.1720 | 7.2663 | 0.2797 | 0.0163 | 9.298 | 4.177 | 0.0962 | 0.00199 | 81 | |
| | | | | | M Hetero | | | 0.58 | 0.46 | 0.58 | 0.21 | 1.20 | 0.49 | 70.73 | 0.19 | 0.73 | 0.42 | 1.68 | 2.38 | 34.32 | 0.2567 | 18.70 | 0.0724 | 0.0572 | 11.23 | 354.59 | 0.5111 | 5.1235 | 0.5595 | 0.0483 | 5.264 | 1.743 | 0.3677 | 0.00189 | 504 | |
| | | | | | F WT | | | 0.58 | 0.25 | 0.58 | 0.06 | 0.99 | 0.00 | 27.21 | 0.40 | 1.03 | 0.65 | 0.73 | 0.55 | 18.78 | 0.0183 | 79.81 | 0.0117 | 0.0271 | 20.82 | 571.49 | 0.0396 | 2.3579 | 0.1214 | 0.0645 | 8.897 | 6.050 | 0.1955 | 0.00199 | 99 | |
| | | | | | F Hetero | | | 1.00 | 0.35 | 0.00 | 0.12 | 0.72 | 0.06 | 157.74 | 0.09 | 0.49 | 0.42 | 1.07 | 0.79 | 10.35 | 0.0182 | 9.88 | 0.0093 | 0.0070 | 5.66 | 11.81 | 0.0397 | 3.3769 | 0.1062 | 0.0765 | 2.889 | 1.830 | 0.3934 | 0.00027 | 2642 | |
| 32 | 21-W266 | *Ywhag* | *KTPU8* | 【Mean】 | M WT | 6 | 14-15 | 149.50 | 4.05 | 112.17 | 9.68 | 6.50 | 2.35 | 270.00 | 6.64 | 14.52 | 23.72 | 27.27 | 29.31 | 508.43 | 0.6605 | 1169.35 | 0.6959 | 0.5947 | 237.95 | 5534.04 | 1.3297 | 75.0581 | 2.4253 | 0.0415 | 18.878 | 7.784 | 0.4571 | 0.00335 | 13289 | + |
| | 1461 | 5 | *pU-21W* | | M Hetero | 6 | 14-15 | 150.17 | 4.02 | 111.50 | 9.47 | 6.17 | 2.63 | 273.67 | 6.97 | 14.08 | 22.23 | 28.09 | 28.59 | 509.08 | 0.7117 | 1179.03 | 0.6952 | 0.5890 | 240.04 | 5457.00 | 1.4125 | 76.4703 | 2.5202 | 0.0357 | 19.257 | 8.070 | 0.5506 | 0.00403 | 12098 | |
| | | B6;CB-*YwhagGt(pU-21W)266Card* | | | F WT | 6 | 14-15 | 151.17 | 3.60 | 115.00 | 9.62 | 7.62 | 2.32 | 383.50 | 7.84 | 14.63 | 20.11 | ND | 23.85 | 442.52 | 0.2239 | 1172.28 | 0.5738 | 0.4891 | 220.46 | 4918.94 | 0.5030 | 65.3144 | 1.4844 | 0.1064 | 16.211 | 8.389 | 0.3867 | 0.00292 | 11038 | |
| | | | | | F Hetero | 6 | 14-15 | 151.33 | 3.90 | 115.67 | 9.72 | 7.52 | 2.43 | 374.17 | 6.70 | 12.20 | 18.52 | ND | 21.97 | 441.93 | 0.2175 | 1172.47 | 0.5252 | 0.4478 | 213.05 | 4674.73 | 0.4843 | 64.6968 | 1.5547 | 0.0910 | 14.668 | 8.413 | 0.4448 | 0.00248 | 9494 | |
| | | | | 【SD】 | M WT | | | 0.84 | 0.30 | 1.94 | 0.20 | 0.53 | 0.10 | 71.21 | 0.95 | 3.20 | 1.86 | 1.26 | 1.24 | 31.26 | 0.1123 | 24.49 | 0.0644 | 0.0476 | 21.91 | 213.11 | 0.2013 | 5.7565 | 0.1986 | 0.0186 | 1.632 | 0.995 | 0.1271 | 0.00116 | 2269 | |
| | | | | | M Hetero | | | 1.83 | 0.34 | 2.59 | 0.39 | 0.61 | 0.29 | 109.84 | 1.49 | 3.91 | 2.94 | 1.68 | 3.06 | 39.02 | 0.2115 | 19.08 | 0.0801 | 0.0606 | 14.44 | 343.78 | 0.3654 | 7.2591 | 0.2590 | 0.0129 | 3.822 | 1.243 | 0.1390 | 0.00114 | 3395 | |
| | | | | | F WT | | | 2.40 | 0.37 | 2.10 | 0.26 | 1.76 | 0.12 | 56.34 | 1.57 | 3.28 | 1.46 | ND | 1.84 | 35.24 | 0.0892 | 12.52 | 0.0696 | 0.0549 | 14.44 | 317.81 | 0.1622 | 6.0552 | 0.2865 | 0.0500 | 2.532 | 0.740 | 0.0851 | 0.00070 | 2913 | |
| | | | | | F Hetero | | | 2.07 | 0.65 | 3.01 | 0.30 | 0.83 | 0.22 | 120.39 | 1.65 | 3.41 | 2.00 | ND | 1.56 | 43.25 | 0.0966 | 11.28 | 0.0328 | 0.0246 | 8.00 | 132.96 | 0.1686 | 7.0767 | 0.3250 | 0.0398 | 1.866 | 0.781 | 0.0433 | 0.00052 | 2333 | |
| 33 | 21-W267 | *Hdac4* | *KTPU8* | 【Mean】 | M WT | 4 | 14 | 153.00 | 4.20 | 109.75 | 8.83 | 7.40 | 3.18 | 287.50 | 5.07 | 12.50 | 21.30 | 25.44 | 27.94 | 494.33 | 0.5233 | 1170.73 | 0.6186 | 0.5282 | 220.93 | 5237.88 | 1.0783 | 74.7712 | 1.9639 | 0.1031 | 20.739 | 9.499 | 1.1338 | 0.00532 | 5635 | − |
| | 1457 | 1 | *pU-21W* | | M Hetero | 3 | 14 | 151.00 | 4.37 | 110.67 | 8.53 | 7.87 | 2.70 | 330.33 | 4.22 | 9.69 | 18.70 | 23.04 | 25.68 | 498.43 | 0.5303 | 1180.97 | 0.6210 | 0.5256 | 223.85 | 5170.13 | 1.0842 | 73.6505 | 2.2799 | 0.0575 | 20.763 | 9.764 | 1.0619 | 0.00555 | 4773 | |
| | | B6;CB-*Hdac4Gt(pU-21W)267Card* | | | F WT | 3 | 14 | 150.67 | 4.47 | 113.00 | 8.77 | 7.43 | 2.33 | 402.33 | 5.64 | 11.97 | 18.39 | 20.02 | 21.21 | 376.37 | 0.0691 | 1200.23 | 0.4990 | 0.4157 | 215.53 | 4363.49 | 0.1754 | 54.0395 | 0.8199 | 0.2940 | 18.538 | 12.229 | 1.0626 | 0.00530 | 3997 | |
| | | | | | F Hetero | 3 | 14 | 149.33 | 4.43 | 112.67 | 8.67 | 8.30 | 2.37 | 413.00 | 4.27 | 9.29 | 17.64 | 19.32 | 20.76 | 398.03 | 0.0765 | 1203.17 | 0.4968 | 0.4128 | 218.30 | 4303.35 | 0.1866 | 56.6522 | 0.7799 | 0.2606 | 14.630 | 9.838 | 0.9530 | 0.00385 | 4066 | |
| | | | | 【SD】 | M WT | | | 5.42 | 0.92 | 2.87 | 0.42 | 0.29 | 0.57 | 58.16 | 0.22 | 2.40 | 1.01 | 0.80 | 0.63 | 12.93 | 0.1473 | 18.30 | 0.0443 | 0.0337 | 8.97 | 191.72 | 0.3083 | 2.2177 | 0.4187 | 0.0214 | 3.326 | 1.458 | 0.3894 | 0.00086 | 2868 | |
| | | | | | M Hetero | | | 0.00 | 0.15 | 1.53 | 0.31 | 0.95 | 0.10 | 52.97 | 0.19 | 0.46 | 1.18 | 1.30 | 0.47 | 18.95 | 0.0470 | 11.47 | 0.0447 | 0.0329 | 12.37 | 98.82 | 0.0604 | 3.5052 | 0.1968 | 0.0130 | 0.818 | 0.400 | 0.0483 | 0.00053 | 540 | |
| | | | | | F WT | | | 1.15 | 0.57 | 1.73 | 0.21 | 1.27 | 0.32 | 44.61 | 0.42 | 0.27 | 0.70 | 0.72 | 0.85 | 26.53 | 0.0320 | 11.42 | 0.0196 | 0.0127 | 7.04 | 23.55 | 0.0767 | 5.7645 | 0.2088 | 0.1193 | 1.663 | 0.987 | 0.1538 | 0.00047 | 401 | |
| | | | | | F Hetero | | | 1.15 | 0.38 | 0.58 | 0.25 | 0.26 | 0.23 | 19.47 | 0.11 | 0.33 | 2.36 | 1.87 | 1.78 | 21.33 | 0.0257 | 6.71 | 0.0417 | 0.0326 | 12.79 | 156.68 | 0.0584 | 2.4488 | 0.1353 | 0.0490 | 3.563 | 1.649 | 0.2182 | 0.00069 | 583 | |
| 34 | 21-W268 | *Lbr* | *KTPU8* | 【Mean】 | M WT | 3 | 14 | 149.00 | 4.87 | 113.00 | 8.90 | 7.10 | 2.23 | 339.33 | 7.72 | 16.73 | 23.41 | 26.31 | 29.54 | 516.80 | 0.4258 | 1178.80 | 0.6761 | 0.5736 | 240.00 | 5354.43 | 0.8342 | 79.3716 | 1.5973 | 0.1179 | 24.167 | 10.976 | 1.4447 | 0.00529 | 12500 | + |
| | 1909 | 1 | *pU-21W* | | M Hom | 3 | 14 | 150.00 | 4.90 | 111.67 | 8.77 | 8.17 | 2.17 | 257.67 | 5.85 | 10.70 | 18.64 | 21.57 | 22.81 | 426.07 | 0.1775 | 1185.93 | 0.5475 | 0.4619 | 216.69 | 4749.72 | 0.4129 | 62.4144 | 1.2711 | 0.1992 | 13.330 | 7.432 | 1.2569 | 0.00359 | 5015 | |
| | | B6;CB-*LbrGt(pU-21W)268Card* | | | F WT | 3 | 15 | 151.67 | 4.60 | 115.00 | 9.07 | 7.80 | 2.43 | 450.00 | 7.76 | 16.13 | 20.55 | 21.11 | 23.14 | 375.73 | 0.0636 | 1200.70 | 0.5940 | 0.4946 | 225.43 | 4912.37 | 0.1624 | 54.0572 | 0.6770 | 0.3657 | 20.803 | 10.857 | 1.3337 | 0.00587 | 4136 | |
| | | | | | F Hom | 3 | 15 | 151.00 | 4.57 | 113.67 | 8.83 | 8.23 | 1.87 | 293.00 | 4.97 | 8.64 | 14.19 | 16.70 | 17.58 | 405.83 | 0.0419 | 1215.80 | 0.5138 | 0.4226 | 224.94 | 4325.41 | 0.0990 | 57.6811 | 0.4143 | 0.4281 | 17.320 | 11.749 | 1.3272 | 0.00410 | 6521 | |
| | | | | 【SD】 | M WT | | | 1.73 | 0.21 | 2.00 | 0.17 | 0.70 | 0.21 | 34.65 | 0.62 | 0.95 | 1.47 | 1.37 | 1.19 | 10.89 | 0.1152 | 26.76 | 0.0174 | 0.0060 | 19.57 | 257.63 | 0.2154 | 2.3991 | 0.2301 | 0.0270 | 2.046 | 1.532 | 0.5032 | 0.00208 | 6022 | |
| | | | | | M Hom | | | 1.00 | 0.46 | 1.53 | 0.60 | 1.40 | 0.06 | 41.10 | 0.13 | 2.87 | 1.02 | 1.84 | 1.98 | 20.83 | 0.0787 | 11.71 | 0.0302 | 0.0294 | 7.54 | 298.77 | 0.1723 | 4.1864 | 0.1374 | 0.0530 | 1.176 | 0.374 | 0.2818 | 0.00036 | 1511 | |
| | | | | | F WT | | | 0.58 | 0.46 | 1.00 | 0.12 | 0.61 | 0.15 | 36.59 | 0.18 | 0.63 | 0.64 | 0.77 | 0.75 | 19.87 | 0.0163 | 23.07 | 0.0230 | 0.0135 | 11.45 | 129.61 | 0.0398 | 1.0300 | 0.1648 | 0.1171 | 0.541 | 0.818 | 0.1649 | 0.00101 | 3620 | |
| | | | | | F Hom | | | 1.00 | 0.29 | 1.53 | 0.21 | 0.72 | 0.06 | 18.68 | 1.22 | 2.41 | 3.23 | 3.07 | 3.65 | 25.83 | 0.0211 | 15.80 | 0.0323 | 0.0270 | 11.80 | 275.68 | 0.0456 | 2.0273 | 0.1315 | 0.1581 | 2.193 | 2.169 | 0.3112 | 0.00101 | 3500 | |
| 35 | 21-W302 | *Umps* | *KTPU8* | 【Mean】 | M WT | 4 | 14 | 152.50 | 4.20 | 115.50 | 9.58 | 5.90 | 2.21 | 289.25 | 6.97 | 17.40 | 24.23 | 28.58 | 30.39 | 450.93 | 0.3262 | 1223.45 | 0.6840 | 0.5571 | 245.30 | 5124.41 | 0.7192 | 67.5938 | 1.7261 | 0.1005 | 19.484 | 9.189 | 0.5243 | 0.00443 | 13041 | + |
| | 1624 | 16 | *pU-21W* | | M Hetero | 4 | 14-15 | 150.50 | 3.78 | 112.50 | 9.45 | 6.20 | 2.10 | 289.00 | 6.61 | 11.40 | 21.00 | 25.15 | 26.87 | 513.63 | 0.4785 | 1220.95 | 0.6983 | 0.5714 | 244.58 | 5246.87 | 0.9472 | 76.3584 | 1.8896 | 0.1371 | 21.194 | 9.662 | 0.4251 | 0.00426 | 14028 | |
| | | B6;CB-*UmpsGt(pU-21W)302Card* | | | F WT | 4 | 14-15 | 151.50 | 3.86 | 116.75 | 9.48 | 7.13 | 2.15 | 361.50 | 6.14 | 12.91 | 17.04 | 18.87 | 20.32 | 382.75 | 0.0460 | 1197.13 | 0.5271 | 0.4382 | 210.62 | 4610.01 | 0.1160 | 55.9852 | 0.5465 | 0.4844 | 14.961 | 8.618 | 0.4402 | 0.00285 | 11953 | |
| | | | | | F Hetero | 4 | 15 | 151.25 | 3.58 | 115.75 | 9.30 | 5.88 | 2.08 | 353.00 | 7.05 | 14.89 | 19.71 | 21.45 | 24.44 | 400.13 | 0.0710 | 1217.28 | 0.5787 | 0.4753 | 229.41 | 4687.95 | 0.1747 | 58.7354 | 0.7363 | 0.3287 | 17.485 | 9.945 | 0.3512 | 0.00262 | 12846 | |
| | | | | 【SD】 | M WT | | | 2.38 | 0.29 | 1.91 | 0.10 | 0.59 | 0.07 | 21.44 | 4.23 | 6.02 | 5.67 | 6.28 | 6.28 | 34.38 | 0.0983 | 40.57 | 0.1256 | 0.0887 | 34.25 | 388.61 | 0.1744 | 4.9809 | 0.1362 | 0.0159 | 4.543 | 1.063 | 0.0700 | 0.00148 | 2968 | |
| | | | | | M Hetero | | | 1.29 | 0.76 | 1.29 | 0.47 | 1.10 | 0.18 | 12.08 | 2.55 | 3.62 | 2.59 | 3.75 | 3.31 | 32.96 | 0.1409 | 15.01 | 0.0827 | 0.0623 | 18.41 | 270.01 | 0.2604 | 3.5960 | 0.3757 | 0.0792 | 2.817 | 0.970 | 0.1294 | 0.00101 | 1227 | |
| | | | | | F WT | | | 2.38 | 0.44 | 2.75 | 0.37 | 4.06 | 0.24 | 59.89 | 2.95 | 5.79 | 5.86 | 4.77 | 4.29 | 24.79 | 0.0113 | 47.95 | 0.1022 | 0.0694 | 24.09 | 299.49 | 0.0303 | 4.1957 | 0.1277 | 0.1059 | 5.575 | 2.392 | 0.1124 | 0.00106 | 3772 | |
| | | | | | F Hetero | | | 1.89 | 0.43 | 2.87 | 0.32 | 1.52 | 0.22 | 52.06 | 1.20 | 1.08 | 1.04 | 0.68 | 1.00 | 18.93 | 0.0255 | 10.67 | 0.0314 | 0.0227 | 11.43 | 185.01 | 0.0685 | 2.3571 | 0.2091 | 0.0927 | 2.808 | 0.915 | 0.2149 | 0.00064 | 2441 | |
| 36 | 21-W319 | *Rcl1* | *KTPU8* | 【Mean】 | M WT | 3 | 15 | 152.33 | 3.70 | 114.00 | 9.27 | 5.57 | 2.30 | 292.67 | 4.97 | 11.28 | 21.49 | 25.86 | 27.83 | 497.10 | 0.4157 | 1210.20 | 0.7136 | 0.5895 | 246.40 | 5385.42 | 0.8349 | 74.0299 | 1.7574 | 0.1277 | 20.105 | 8.713 | 0.4870 | 0.00399 | 13443 | + |
| | 1527 | 19 | *pU-21W* | | M Hetero | 3 | 15 | 149.33 | 4.17 | 113.00 | 9.77 | 5.47 | 2.47 | 278.33 | 5.21 | 9.44 | 21.52 | 24.35 | 26.89 | 527.10 | 0.5659 | 1200.37 | 0.6936 | 0.5778 | 238.11 | 5404.14 | 1.1066 | 77.4744 | 2.1200 | 0.0620 | 19.748 | 8.491 | 0.4647 | 0.00389 | 12472 | |
| | | B6;CB-*Rcl1Gt(pU-21W)319Card* | | | F WT | 3 | 15 | 150.00 | 3.63 | 116.00 | 9.13 | 4.27 | 2.10 | 362.67 | 6.11 | 11.98 | 18.00 | 19.13 | 20.21 | 416.27 | 0.1032 | 1167.83 | 0.5104 | 0.4370 | 202.61 | 4717.93 | 0.2439 | 60.0785 | 0.9150 | 0.2365 | 13.657 | 7.701 | 0.4546 | 0.00249 | 9375 | |
| | | | | | F Hetero | 3 | 15-16 | 151.33 | 3.73 | 116.67 | 9.17 | 6.10 | 2.13 | 384.67 | 5.77 | 12.84 | 17.77 | 19.10 | 19.94 | 401.50 | 0.1089 | 1205.17 | 0.5602 | 0.4643 | 226.04 | 4631.95 | 0.2595 | 60.6901 | 0.9178 | 0.2510 | 16.123 | 9.424 | 0.3500 | 0.00217 | 12090 | |
| | | | | 【SD】 | M WT | | | 3.79 | 0.30 | 1.73 | 0.32 | 1.12 | 0.26 | 19.35 | 0.48 | 3.38 | 4.21 | 4.35 | 5.13 | 52.73 | 0.1737 | 7.24 | 0.0771 | 0.0620 | 11.71 | 337.61 | 0.2757 | 7.5524 | 0.1757 | 0.0484 | 2.566 | 0.889 | 0.1808 | 0.00157 | 1967 | |
| | | | | | M Hetero | | | 1.53 | 0.25 | 2.65 | 0.23 | 0.50 | 0.35 | 33.01 | 0.14 | 0.61 | 1.46 | 1.56 | 1.51 | 20.28 | 0.1471 | 9.87 | 0.0220 | 0.0136 | 4.16 | 46.72 | 0.3204 | 1.9166 | 0.4250 | 0.0344 | 1.564 | 0.530 | 0.0966 | 0.00106 | 835 | |
| | | | | | F WT | | | 1.73 | 0.21 | 2.00 | 0.38 | 0.47 | 0.10 | 22.50 | 1.02 | 2.09 | 1.38 | 1.04 | 1.99 | 11.87 | 0.0190 | 13.90 | 0.0202 | 0.0154 | 3.55 | 127.86 | 0.0457 | 0.9056 | 0.1335 | 0.0374 | 1.479 | 0.610 | 0.0255 | 0.00020 | 1800 | |
| | | | | | F Hetero | | | 1.15 | 0.78 | 4.04 | 0.25 | 2.44 | 0.06 | 45.08 | 2.06 | 3.34 | 2.04 | 1.45 | 1.24 | 39.58 | 0.0600 | 16.31 | 0.0745 | 0.0561 | 10.03 | 359.93 | 0.1328 | 4.9815 | 0.2846 | 0.0833 | 2.244 | 0.365 | 0.0403 | 0.00043 | 3131 | |
| 37 | 21-W321 | *1600020E01Rik; lincRNA* | *KTPU8* | 【Mean】 | M WT | 3 | 14-15 | 152.00 | 4.87 | 115.00 | 9.10 | 9.77 | 2.47 | 339.33 | 5.71 | 15.96 | 22.70 | 25.05 | 27.67 | 499.47 | 0.3892 | 1207.67 | 0.6141 | 0.5086 | 235.54 | 4905.76 | 0.7910 | 75.1249 | 1.7808 | 0.0879 | 18.400 | 9.673 | 1.0256 | 0.00417 | 6118 | + |
| | 1831 | 6 | *pU-21W* | | M Hetero | 3 | 14-15 | 153.33 | 4.83 | 117.33 | 9.17 | 9.27 | 2.37 | 306.00 | 5.34 | 14.64 | 23.09 | 24.78 | 27.36 | 469.00 | 0.3517 | 1182.67 | 0.6242 | 0.5272 | 223.54 | 5227.07 | 0.7402 | 69.9813 | 1.5488 | 0.1652 | 17.527 | 8.022 | 0.6931 | 0.00380 | 9425 | |
| | | B6;CB-*1600020E01RikGt(pU-21W)321Card* | | | F WT | 3 | 14-16 | 152.00 | 4.23 | 116.00 | 9.00 | 8.67 | 2.50 | 465.00 | 5.12 | 13.76 | 20.24 | 20.98 | 22.70 | 391.13 | 0.1181 | 1206.47 | 0.5566 | 0.4607 | 224.05 | 4641.58 | 0.2973 | 56.3234 | 1.1361 | 0.1753 | 17.185 | 10.045 | 0.7225 | 0.00361 | 8258 | |
| | | | | | F Hetero | 4 | 14-16 | 152.75 | 4.48 | 115.50 | 8.83 | 7.70 | 2.33 | 376.50 | 6.03 | 14.35 | 20.59 | 21.43 | 22.68 | 404.70 | 0.1413 | 1203.13 | 0.6044 | 0.5025 | 221.18 | 5055.19 | 0.3374 | 58.3439 | 1.0881 | 0.2451 | 20.608 | 10.045 | 0.6463 | 0.00432 | 15010 | |
| | | | | 【SD】 | M WT | | | 1.00 | 0.12 | 2.00 | 0.10 | 0.23 | 0.06 | 31.34 | 0.21 | 1.57 | 1.11 | 0.91 | 0.36 | 29.87 | 0.0525 | 14.93 | 0.0294 | 0.0251 | 10.79 | 147.98 | 0.1073 | 5.7542 | 0.1736 | 0.0276 | 3.207 | 2.182 | 0.3512 | 0.00130 | 1152 | |
| | | | | | M Hetero | | | 0.58 | 0.06 | 2.08 | 0.06 | 0.75 | 0.06 | 15.87 | 0.23 | 0.29 | 0.73 | 0.64 | 0.68 | 57.69 | 0.1589 | 20.77 | 0.0865 | 0.0647 | 27.83 | 151.39 | 0.2906 | 10.6434 | 0.4501 | 0.1454 | 3.085 | 0.946 | 0.2031 | 0.00119 | 2657 | |
| | | | | | F WT | | | 1.00 | 0.46 | 1.73 | 0.10 | 0.67 | 0.17 | 66.02 | 0.42 | 0.97 | 0.81 | 0.87 | 1.55 | 10.71 | 0.0132 | 16.36 | 0.1004 | 0.0772 | 8.91 | 532.64 | 0.0315 | 1.4017 | 0.2193 | 0.0390 | 3.449 | 1.346 | 0.1957 | 0.00093 | 2721 | |
| | | | | | F Hetero | | | 0.50 | 0.59 | 1.29 | 0.17 | 1.22 | 0.22 | 36.35 | 0.45 | 1.85 | 0.35 | 0.75 | 0.59 | 30.55 | 0.0726 | 22.83 | 0.0364 | 0.0325 | 9.33 | 232.56 | 0.1636 | 3.9849 | 0.3569 | 0.0761 | 3.217 | 1.802 | 0.3152 | 0.00079 | 4330 | |
| 38 | 21-W361 | *Dym* | *KTPU8* | 【Mean】 | M WT | 3 | 15 | 150.33 | 5.30 | 112.67 | 8.97 | 8.97 | 2.80 | 298.00 | 7.14 | 15.85 | 22.03 | 24.69 | 27.38 | 503.57 | 0.2867 | 1173.87 | 0.6033 | 0.5143 | 222.34 | 5105.77 | 0.5786 | 74.5832 | 1.2588 | 0.1958 | 21.512 | 10.252 | 0.7348 | 0.00468 | 13128 | + |
| | 2049 | 18 | *pU-21W* | | M Hom | 3 | 15 | 150.33 | 5.33 | 113.33 | 8.97 | 8.93 | 2.73 | 334.00 | 6.18 | ND | 20.13 | 22.47 | 25.53 | 492.70 | 0.2673 | 1181.90 | 0.5560 | 0.4705 | 222.88 | 4737.83 | 0.5508 | 75.3559 | 1.3572 | 0.1690 | 11.510 | 6.424 | 0.6834 | 0.00243 | 6735 | |
| | | B6;CB-*DymGt(pU-21W)361Card* | | | F WT | 3 | 14-15 | 149.67 | 4.47 | 112.00 | 8.93 | 7.60 | 2.60 | 420.67 | 7.14 | 13.31 | 18.19 | 19.66 | 20.76 | 353.77 | 0.0415 | 1182.43 | 0.4963 | 0.4197 | 207.81 | 4507.92 | 0.1111 | 53.7449 | 0.5879 | 0.4307 | 13.957 | 8.660 | 0.4772 | 0.00232 | 10127 | |
| | | | | | F Hom | 3 | 15-16 | 151.33 | 4.20 | 113.33 | 8.97 | 7.37 | 2.53 | 422.00 | 6.69 | 10.27 | 17.04 | 18.20 | 19.35 | 395.53 | 0.0575 | 1181.87 | 0.4847 | 0.4098 | 208.17 | 4425.68 | 0.1419 | 57.0958 | 0.6093 | 0.3461 | 14.818 | 9.584 | 0.5362 | 0.00238 | 10316 | |
| | | | | 【SD】 | M WT | | | 1.15 | 0.20 | 1.53 | 0.42 | 1.36 | 0.17 | 15.72 | 1.07 | ND | 2.00 | 2.58 | 2.13 | 14.59 | 0.0517 | 12.22 | 0.0186 | 0.0211 | 3.95 | 245.92 | 0.1177 | 5.7350 | 0.2547 | 0.0569 | 5.472 | 1.766 | 0.2679 | 0.00142 | 2869 | |
| | | | | | M Hom | | | 0.58 | 0.64 | 1.15 | 0.25 | 2.44 | 0.21 | 7.00 | 1.23 | ND | 2.39 | 2.26 | 2.62 | 17.80 | 0.0743 | 21.61 | 0.0316 | 0.0188 | 7.67 | 55.41 | 0.1756 | 3.1902 | 0.5314 | 0.0939 | 4.110 | 2.225 | 0.0526 | 0.00101 | 987 | |
| | | | | | F WT | | | 3.21 | 0.49 | 1.00 | 0.50 | 1.44 | 0.26 | 27.97 | 1.44 | 2.31 | 0.50 | 0.69 | 0.88 | 16.23 | 0.0166 | 20.31 | 0.0136 | 0.0052 | 9.77 | 126.95 | 0.0408 | 2.1593 | 0.1745 | 0.0915 | 0.709 | 0.920 | 0.0403 | 0.00023 | 2835 | |
| | | | | | F Hom | | | 0.58 | 0.36 | 3.06 | 0.38 | 0.95 | 0.21 | 50.03 | 1.10 | 0.16 | 0.91 | 1.07 | 1.28 | 24.71 | 0.0299 | 6.64 | 0.0276 | 0.0225 | 6.95 | 150.76 | 0.0788 | 2.6892 | 0.3094 | 0.1518 | 2.057 | 1.892 | 0.1215 | 0.00043 | 4373 | |
| 39 | 21-W367 | *Ptpn11* | *KTPU8* | 【Mean】 | M WT | 3 | 15-16 | 150.33 | 4.27 | 112.00 | 8.97 | 8.43 | 2.77 | 246.67 | 7.67 | 16.72 | 23.05 | 27.19 | 29.85 | 511.27 | 0.3505 | 1207.63 | 0.6617 | 0.5477 | 249.11 | 5009.26 | 0.6823 | 75.3434 | 1.5832 | 0.1268 | 21.225 | 10.651 | 1.1291 | 0.00481 | 4345 | + |
| | 1910 | 5 | *pU-21W* | | M Hetero | 3 | 15-16 | 150.67 | 5.07 | 111.33 | 8.97 | 9.10 | 2.73 | 272.00 | 7.74 | 17.53 | 24.52 | 27.94 | 30.66 | 497.37 | 0.5669 | 1189.00 | 0.6927 | 0.5827 | 235.78 | 5476.90 | 1.1630 | 73.2519 | 2.1975 | 0.0618 | 18.630 | 7.888 | 1.0631 | 0.00458 | 8971 | |
| | | B6;CB-*Ptpn11Gt(pU-21W)367Card* | | | F WT | 3 | 15-16 | 151.67 | 4.43 | 115.00 | 9.10 | 7.00 | 2.40 | 379.67 | 7.09 | 13.65 | 17.27 | 18.54 | 20.24 | 422.73 | 0.1249 | 1226.50 | 0.5259 | 0.4283 | 227.77 | 4314.31 | 0.2920 | 63.3592 | 1.2448 | 0.1391 | 16.285 | 10.997 | 1.1790 | 0.00468 | 3659 | |
| | | | | | F Hetero | 3 | 15-16 | 150.33 | 4.60 | 114.67 | 8.77 | 7.90 | 2.13 | 450.00 | 7.37 | 14.84 | 18.48 | 19.25 | 21.14 | 358.23 | 0.0736 | 1214.17 | 0.5238 | 0.4313 | 209.94 | 4581.61 | 0.1891 | 54.2725 | 0.7885 | 0.2826 | 13.762 | 8.287 | 1.1599 | 0.00398 | 3198 | |
| | | | | 【SD】 | M WT | | | 0.58 | 0.23 | 1.00 | 0.32 | 0.75 | 0.06 | 50.34 | 0.27 | 1.95 | 0.63 | 1.29 | 1.78 | 74.16 | 0.1343 | 11.76 | 0.0628 | 0.0481 | 6.93 | 260.67 | 0.1865 | 11.9206 | 0.0506 | 0.0269 | 2.207 | 1.107 | 0.3303 | 0.00136 | 574 | |
| | | | | | M Hetero | | | 1.53 | 0.32 | 1.53 | 0.06 | 0.56 | 0.21 | 18.68 | 0.74 | 1.77 | 1.31 | 1.20 | 1.81 | 11.08 | 0.0744 | 15.00 | 0.0308 | 0.0291 | 2.73 | 215.07 | 0.1817 | 1.8990 | 0.2269 | 0.0155 | 17.099 | 7.618 | 0.0591 | 0.00384 | 5600 | |
| | | | | | F WT | | | 1.15 | 0.55 | 1.73 | 0.10 | 0.70 | 0.26 | 32.65 | 0.71 | 0.78 | 1.96 | 2.32 | 2.65 | 38.43 | 0.0427 | 23.68 | 0.0609 | 0.0421 | 16.73 | 198.73 | 0.0793 | 5.3173 | 0.2872 | 0.0631 | 2.026 | 1.145 | 0.3830 | 0.00032 | 751 | |
| | | | | | F Hetero | | | 1.15 | 0.36 | 2.52 | 0.23 | 1.06 | 0.21 | 29.46 | 0.15 | 0.54 | 1.22 | 1.40 | 1.31 | 47.52 | 0.0604 | 26.38 | 0.0297 | 0.0200 | 13.22 | 235.98 | 0.1372 | 5.5425 | 0.3076 | 0.1291 | 0.338 | 0.952 | 0.2353 | 0.00078 | 708 | |
| 40 | 21-W373 | *Slc38a2* | *KTPU8* | 【Mean】 | M WT | 3 | 16 | 149.67 | 4.67 | 114.00 | 9.20 | 5.80 | 2.17 | 237.67 | 7.79 | 17.45 | 22.62 | 26.12 | 27.73 | 448.83 | 0.2519 | 1165.43 | 0.5655 | 0.4867 | 216.68 | 4951.81 | 0.5321 | 67.0610 | 1.3443 | 0.1956 | 18.498 | 9.526 | 0.7118 | 0.00312 | 13846 | + |
| | 1775 | 15 | *pU-21W* | | M Homo | 3 | 14-16 | 150.00 | 4.30 | 113.00 | 9.20 | 5.60 | 1.87 | 281.67 | 5.29 | 9.16 | 14.62 | 18.36 | 21.20 | 425.83 | 0.1849 | 1131.53 | 0.4443 | 0.3915 | 193.12 | 4441.68 | 0.4185 | 62.7365 | 1.4443 | 0.1678 | 14.353 | 9.140 | 0.7176 | 0.00260 | 10191 | |
| | | B6;CB-*Slc38a2Gt(pU-21W)373Card* | | | F WT | 3 | 16 | 152.00 | 3.73 | 115.67 | 9.17 | 5.17 | 2.13 | 314.67 | 6.23 | 13.18 | 18.78 | 20.70 | 22.34 | 353.47 | 0.0453 | 1211.80 | 0.5561 | 0.4588 | 221.20 | 4665.18 | 0.1215 | 54.5711 | 0.5988 | 0.3669 | 23.117 | 13.405 | 0.4866 | 0.00359 | 18072 | |
| | | | | | F Homo | 3 | 15-16 | 148.67 | 3.80 | 110.67 | 9.27 | 6.23 | 2.17 | 319.33 | 5.48 | 8.41 | 15.12 | 16.85 | 19.34 | 374.13 | 0.0217 | 1156.13 | 0.4339 | 0.3743 | 196.28 | 4237.52 | 0.0552 | 53.0769 | 0.3214 | 0.5138 | 17.710 | 12.398 | 0.3818 | 0.00285 | 13520 | |
| | | | | 【SD】 | M WT | | | 1.53 | 0.58 | 2.00 | 0.53 | 1.81 | 0.40 | 8.62 | 0.35 | 0.86 | 2.29 | 0.85 | 3.93 | 52.99 | 0.2348 | 28.58 | 0.0923 | 0.0922 | 6.48 | 697.25 | 0.4467 | 8.4356 | 0.5063 | 0.0188 | 3.463 | 1.015 | 0.2076 | 0.00062 | 1139 | |
| | | | | | M Homo | | | 0.00 | 0.17 | 2.00 | 0.40 | 1.57 | 0.42 | 23.35 | 0.36 | 0.32 | 4.94 | 3.41 | 1.44 | 61.99 | 0.1189 | 38.45 | 0.0707 | 0.0498 | 26.48 | 136.37 | 0.2392 | 8.7616 | 0.5605 | 0.0835 | 0.950 | 0.394 | 0.1976 | 0.00033 | 2982 | |
| | | | | | F WT | | | 1.00 | 0.55 | 2.08 | 0.06 | 0.25 | 0.15 | 71.44 | 0.77 | 1.73 | 0.63 | 1.60 | 1.73 | 27.16 | 0.0170 | 18.03 | 0.0219 | 0.0136 | 10.87 | 171.79 | 0.0394 | 3.3551 | 0.2129 | 0.1129 | 1.175 | 1.532 | 0.1170 | 0.00017 | 1141 | |
| | | | | | F Homo | | | 1.53 | 0.44 | 1.53 | 0.45 | 0.74 | 0.35 | 6.66 | 0.30 | 1.52 | 0.81 | 1.51 | 0.96 | 19.54 | 0.0027 | 59.33 | 0.0546 | 0.0290 | 21.26 | 71.11 | 0.0081 | 3.0725 | 0.0704 | 0.0314 | 0.771 | 0.603 | 0.0406 | 0.00062 | 1080 | |
| 41 | 21-W406 | *Ndufaf1* | *KTPU8* | 【Mean】 | M WT | 3 | 15-16 | 149.33 | 4.77 | 110.33 | 9.07 | 7.23 | 2.57 | 268.67 | 7.14 | 16.80 | 22.75 | 24.95 | 26.39 | 461.30 | 0.3777 | 1147.30 | 0.5587 | 0.4872 | 206.40 | 5123.61 | 0.8115 | 68.1850 | 1.7615 | 0.1171 | 15.358 | 7.339 | 1.2392 | 0.00400 | 4898 | − |
| | 1752 | 2 | *pU-21W* | | M Hetero | 3 | 15-16 | 148.33 | 3.90 | 108.33 | 8.67 | 6.60 | 2.10 | 268.33 | 8.49 | 18.32 | 23.73 | 27.20 | 29.24 | 432.03 | 0.3341 | 1120.60 | 0.5383 | 0.4803 | 196.63 | 5236.80 | 0.7444 | 63.0396 | 1.6685 | 0.1212 | 14.425 | 6.505 | 0.9481 | 0.00349 | 5333 | |
| | | B6;CB-*Ndufaf1Gt(pU-21W)406Card* | | | F WT | 3 | 15-16 | 149.33 | 4.33 | 110.67 | 9.07 | 8.50 | 2.33 | 354.67 | 7.84 | 14.95 | 19.37 | 20.15 | 21.58 | 383.33 | 0.1042 | 1171.67 | 0.4923 | 0.4201 | 207.84 | 4505.34 | 0.2609 | 53.4594 | 0.9770 | 0.2309 | 15.309 | 9.492 | 1.1813 | 0.00342 | 5917 | |
| | | | | | F Hetero | 3 | 15-16 | 148.33 | 4.13 | 111.00 | 8.90 | 7.97 | 2.23 | 345.67 | 7.76 | 15.38 | 18.65 | 20.12 | 21.55 | 384.40 | 0.0917 | 1153.43 | 0.4742 | 0.4111 | 200.44 | 4515.57 | 0.2320 | 54.6241 | 0.9355 | 0.2557 | 15.535 | 9.564 | 0.9021 | 0.00353 | 9135 | |
| | | | | 【SD】 | M WT | | | 1.15 | 0.31 | 1.15 | 0.40 | 0.47 | 0.25 | 12.10 | 0.52 | 0.78 | 0.74 | 0.53 | ND | 45.27 | 0.1451 | 7.66 | 0.0572 | 0.0531 | 4.70 | 385.29 | 0.2655 | 7.1705 | 0.1754 | 0.0184 | 2.144 | 0.473 | 0.1351 | 0.00022 | 1224 | |
| | | | | | M Hetero | | | 1.53 | 0.60 | 2.08 | 0.12 | 0.92 | 0.00 | 12.66 | 0.23 | 0.71 | 0.58 | 1.33 | 1.70 | 43.90 | 0.2420 | 2.62 | 0.0607 | 0.0530 | 4.95 | 412.08 | 0.4787 | 7.0060 | 0.5952 | 0.0691 | 5.587 | 1.998 | 0.3509 | 0.00099 | 955 | |
| | | | | | F WT | | | 1.15 | 0.49 | 2.08 | 0.15 | 0.52 | 0.15 | 27.79 | 0.78 | 1.44 | 1.34 | 2.31 | 1.76 | 38.06 | 0.0494 | 9.56 | 0.0249 | 0.0180 | 12.41 | 35.59 | 0.1096 | 4.1228 | 0.2716 | 0.0961 | 3.031 | 1.964 | 0.0776 | 0.00079 | 779 | |
| | | | | | F Hetero | | | 0.58 | 0.59 | 2.00 | 0.20 | 1.22 | 0.32 | 5.51 | 0.50 | 0.64 | 0.84 | 0.99 | 0.01 | 23.12 | 0.0175 | 16.94 | 0.0096 | 0.0035 | 5.81 | 58.05 | 0.0333 | 2.6749 | 0.0474 | 0.0124 | 1.512 | 0.734 | 0.0801 | 0.00108 | 4973 | |
| 42 | 21-W426 | *D19Wsu162e* | *KTPU8* | 【Mean】 | M WT | 3 | 14-16 | 150.33 | 5.00 | 114.00 | 9.37 | 6.03 | 2.37 | 275.33 | 7.29 | 14.72 | 20.83 | 24.94 | 28.97 | 525.07 | 0.6226 | 1197.63 | 0.7123 | 0.5949 | 246.62 | 5426.64 | 1.2047 | 78.9964 | 2.2247 | 0.0786 | 27.130 | 11.530 | 0.7057 | 0.00554 | 17100 | + |
| | 1740 | 19 | *pU-21W* | | M Homo | 3 | 15-16 | 152.33 | 5.10 | 115.00 | 9.20 | 6.93 | 2.23 | 279.33 | 6.89 | 12.01 | 21.68 | 23.80 | 24.51 | 532.83 | 0.5048 | 1200.90 | 0.6421 | 0.5347 | 236.51 | 5107.66 | 0.9678 | 79.8837 | 1.9480 | 0.0804 | 22.822 | 10.967 | 0.4903 | 0.00420 | 16003 | |
| | | B6;CB-*D19Wsu162eGt(pU-21W)426Card* | | | F WT | 3 | 14-16 | 151.00 | 4.60 | 115.67 | 9.37 | 4.47 | 2.27 | 406.33 | 8.23 | 13.06 | 18.95 | 19.73 | ND | 413.80 | 0.1059 | 1200.00 | 0.5624 | 0.4687 | 221.19 | 4773.71 | 0.2537 | 60.1560 | 0.9362 | 0.2201 | 19.252 | 10.729 | 0.5817 | 0.00365 | 13421 | |
| | | | | | F Homo | 3 | 14 | 151.00 | 4.17 | 114.33 | 9.57 | 4.53 | 2.13 | 437.67 | 7.90 | 11.63 | 16.35 | 18.16 | ND | 417.23 | 0.1430 | 1185.27 | 0.5379 | 0.4529 | 211.54 | 4754.12 | 0.3381 | 60.7825 | 1.2060 | 0.1503 | 17.878 | 9.941 | 0.6514 | 0.00346 | 14746 | |
| | | | | 【SD】 | M WT | | | 1.53 | 0.26 | 2.00 | 0.21 | 1.15 | 0.06 | 31.53 | 1.65 | 4.53 | 2.69 | 2.88 | 0.81 | 48.49 | 0.1309 | 7.18 | 0.0371 | 0.0334 | 6.37 | 291.27 | 0.1605 | 8.7168 | 0.2121 | 0.0459 | 4.207 | 1.004 | 0.1393 | 0.00162 | 2090 | |
| | | | | | M Homo | | | 1.53 | 0.53 | 0.00 | 0.10 | 1.02 | 0.25 | 23.76 | 0.34 | 0.68 | 0.86 | 1.61 | ND | 6.65 | 0.0822 | 0.70 | 0.0266 | 0.0218 | 3.59 | 150.52 | 0.1644 | 1.2516 | 0.3528 | 0.0460 | 2.780 | 0.839 | 0.0765 | 0.00086 | 1766 | |
| | | | | | F WT | | | 1.73 | 0.26 | 2.52 | 0.40 | 1.17 | 0.15 | 108.14 | 1.11 | 0.21 | 0.20 | 0.84 | ND | 11.80 | 0.0051 | 36.86 | 0.0201 | 0.0081 | 19.86 | 270.15 | 0.0173 | 1.8797 | 0.0160 | 0.0235 | 1.053 | 1.845 | 0.0897 | 0.00049 | 52 | |
| | | | | | F Homo | | | 1.00 | 0.31 | 0.58 | 0.06 | 0.59 | 0.12 | 60.70 | 1.17 | 0.68 | 0.21 | 1.09 | ND | 46.38 | 0.0214 | 38.57 | 0.0664 | 0.0408 | 25.91 | 1.91 | 0.0160 | 5.4538 | 0.0905 | 0.0447 | 2.838 | 1.583 | 0.2111 | 0.00026 | 1366 | |
| 43 | 21-W456 | *Ppat* | *KTPU8* | 【Mean】 | M WT | 3 | 14-16 | 150.67 | 4.10 | 112.67 | 9.43 | 5.90 | 2.13 | 239.00 | 8.54 | 18.77 | 25.27 | 25.51 | 28.85 | 503.37 | 0.3435 | 1190.57 | 0.6164 | 0.5172 | 228.01 | 5034.62 | 0.6719 | 74.6523 | 1.3827 | 0.2053 | 16.522 | 8.183 | 0.5479 | 0.00369 | 11737 | + |
| | 1755 | 5 | *pU-21W* | | M Hetero | 3 | 14-16 | 152.00 | 4.07 | 114.00 | 9.53 | 6.30 | 2.20 | 283.33 | 7.16 | 14.87 | 23.60 | 25.48 | 30.11 | 541.60 | 0.4560 | 1213.83 | 0.7455 | 0.6138 | 252.49 | 5485.40 | 0.8681 | 81.2224 | 1.5386 | 0.2185 | 16.924 | 6.954 | 0.4771 | 0.00332 | 12817 | |
| | | B6;CB-*PpatGt(pU-21W)456Card* | | | F WT | 3 | 15 | 150.33 | 3.87 | 116.00 | 9.43 | 5.94 | 2.33 | 321.67 | 8.02 | 15.95 | 18.77 | 20.94 | 21.99 | 451.90 | 0.0908 | 1222.63 | 0.5613 | 0.4590 | 223.62 | 4644.38 | 0.2068 | 65.8685 | 0.6887 | 0.3463 | 14.680 | 8.563 | 0.2879 | 0.00224 | 14048 | |
| | | | | | F Hetero | 3 | 14 | 150.33 | 3.70 | 115.00 | 9.47 | 4.77 | 2.03 | 388.33 | 7.31 | 13.55 | 18.79 | 20.90 | 22.42 | 433.37 | 0.1009 | 1198.87 | 0.5164 | 0.4302 | 212.68 | 4527.13 | 0.0935 | 61.1393 | 0.3977 | 0.4943 | 15.094 | 9.230 | 0.3789 | 0.00287 | 10286 | |
| | | | | 【SD】 | M WT | | | 0.58 | 1.18 | 1.53 | 0.15 | 1.13 | 0.23 | 28.16 | 1.70 | 4.14 | 0.24 | ND | 1.20 | 45.94 | 0.2259 | 9.35 | 0.1420 | 0.1152 | 22.77 | 586.28 | 0.3891 | 7.1415 | 0.3741 | 0.0495 | 3.262 | 0.818 | 0.1747 | 0.00180 | 626 | |
| | | | | | M Hetero | | | 2.65 | 0.67 | 2.00 | 0.21 | 1.18 | 0.17 | 35.85 | 1.89 | 4.55 | 2.51 | 1.52 | 2.72 | 25.00 | 0.1440 | 22.09 | 0.0748 | 0.0541 | 10.40 | 267.59 | 0.3164 | 5.3942 | 0.4334 | 0.1300 | 5.457 | 1.717 | 0.3715 | 0.00256 | 3204 | |
| | | | | | F WT | | | 1.53 | 0.31 | 1.00 | 0.21 | 2.29 | 0.21 | 32.59 | 0.30 | 1.26 | 1.37 | ND | 1.06 | 50.65 | 0.0487 | 24.95 | 0.0533 | 0.0425 | 9.76 | 286.76 | 0.1179 | 7.7432 | 0.3988 | 0.1948 | 4.211 | 2.570 | 0.0702 | 0.00073 | 348 | |
| | | | | | F Hetero | | | 2.31 | 0.53 | 1.73 | 0.15 | 0.49 | 0.25 | 16.44 | 0.87 | 1.74 | 0.69 | 0.67 | 1.58 | 35.42 | 0.0131 | 30.97 | 0.0498 | 0.0312 | 17.92 | 51.22 | 0.0382 | 3.4463 | 0.1935 | 0.1272 | 4.254 | 2.492 | 0.0109 | 0.00079 | 5915 | |
| 44 | 21-W473 | *Slc38a4* | *KTPU8* | 【Mean】 | M WT | 3 | 14-16 | 152.00 | 4.57 | 112.67 | 9.13 | 8.47 | 2.53 | 239.00 | 6.85 | 16.50 | 23.09 | 26.81 | 29.22 | 581.70 | 0.2440 | 1173.70 | 0.6425 | 0.5477 | 231.16 | 5265.84 | 0.3886 | 64.1452 | 1.2041 | 0.3266 | 22.912 | 10.433 | 1.1231 | 0.00454 | 7368 | + |
| | 1757 | 15 | *pU-21W* | | M Hetero | 3 | 14 | 148.67 | 3.97 | 109.67 | 9.03 | 8.50 | 2.47 | 278.33 | 6.08 | 13.26 | 21.39 | 21.72 | 27.32 | 439.37 | 0.1469 | 1171.40 | 0.5785 | 0.4934 | 229.86 | 4833.97 | 0.3327 | 65.2465 | 1.2198 | 0.2132 | 19.633 | 10.611 | 0.8080 | 0.00372 | 7263 | |
| | | B6;CB-*Slc38a4Gt(pU-21W)473Card* | | | F WT | 3 | 14 | 151.33 | 4.20 | 115.33 | 9.17 | 8.73 | 2.27 | 362.33 | 6.35 | 13.03 | 17.45 | 19.83 | 21.62 | 368.10 | 0.0505 | 1191.00 | 0.4932 | 0.4134 | 221.27 | 4263.10 | 0.1323 | 54.7290 | 0.7786 | 0.2553 | 17.335 | 12.128 | 0.6582 | 0.00266 | 9095 | |
| | | | | | F Hetero | 3 | 14-15 | 150.33 | 4.53 | 112.33 | 9.23 | 9.53 | 2.27 | 413.67 | 5.81 | 12.10 | 17.50 | 18.66 | 20.32 | 404.90 | 0.0724 | 1199.97 | 0.4625 | 0.3854 | 221.28 | 4027.72 | 0.1747 | 58.6180 | 0.8301 | 0.2264 | 17.368 | 13.460 | 1.3291 | 0.00453 | 4319 | |
| | | | | 【SD】 | M WT | | | 3.00 | 0.15 | 1.15 | 0.32 | 0.75 | 0.31 | 52.37 | 0.92 | 2.42 | 1.84 | 2.43 | 2.57 | 166.04 | 0.0879 | 19.68 | 0.0415 | 0.0403 | 1.79 | 328.51 | 0.2647 | 14.5071 | 0.5056 | 0.2096 | 2.010 | 1.206 | 0.1426 | 0.00025 | 4108 | |
| | | | | | M Hetero | | | 1.53 | 0.29 | 1.53 | 0.06 | 0.36 | 0.25 | 39.72 | 0.07 | 0.97 | 0.71 | 5.42 | 0.85 | 42.67 | 0.0688 | 27.78 | 0.0489 | 0.0303 | 13.34 | 84.79 | 0.1453 | 4.5477 | 0.3997 | 0.1049 | 6.516 | 3.691 | 0.3149 | 0.00197 | 1464 | |
| | | | | | F WT | | | 0.58 | 0.44 | 2.31 | 0.32 | 0.42 | 0.15 | 35.85 | 0.56 | 1.76 | 1.85 | 0.58 | 0.80 | 27.03 | 0.0090 | 39.72 | 0.0551 | 0.0334 | 16.41 | 157.57 | 0.0164 | 3.7574 | 0.1853 | 0.0488 | 3.178 | 3.130 | 0.1845 | 0.00093 | 4942 | |
| | | | | | F Hetero | | | 0.58 | 0.25 | 1.15 | 0.12 | 0.06 | 0.32 | 66.94 | 1.46 | 1.90 | 0.35 | 1.43 | 1.52 | 20.54 | 0.0241 | 12.25 | 0.0120 | 0.0070 | 4.15 | 29.02 | 0.0520 | 2.4688 | 0.1631 | 0.0471 | 4.700 | 3.678 | 0.2666 | 0.00103 | 1723 | |
| 45 | 21-W474 | *EST* | *KTPU8* | 【Mean】 | M WT | 3 | 15 | 148.33 | 4.30 | 109.00 | 8.80 | 7.60 | 2.30 | 291.00 | 9.77 | 16.88 | 21.37 | 23.34 | 25.24 | 497.13 | 0.3306 | 1168.80 | 0.6418 | 0.5488 | 222.82 | 5400.32 | 0.6728 | 73.0937 | 1.4613 | 0.2023 | 17.647 | 7.608 | 0.4916 | 0.00240 | 14356 | + |
| | 1913 | 19 | *pU-21W* | | M Hetero | 3 | 15 | 150.33 | 3.67 | 109.00 | 8.53 | 6.77 | 2.20 | 349.33 | 5.23 | 10.17 | 17.15 | 19.19 | 21.20 | 494.47 | 0.2938 | 1186.87 | 0.5609 | 0.4724 | 216.51 | 4823.56 | 0.5967 | 73.7205 | 1.5589 | 0.1491 | 16.658 | 8.971 | 0.5098 | 0.00243 | 12034 | |
| | | B6;CB-*Gt(pU-21W)474Card* | | | F WT | 3 | 15 | 150.00 | 4.10 | 112.33 | 8.87 | 6.40 | 2.23 | 392.00 | 6.90 | 13.45 | 18.78 | 20.21 | 21.56 | 404.50 | 0.0628 | 1188.33 | 0.5376 | 0.4526 | 211.03 | 4742.35 | 0.1489 | 61.2659 | 0.6358 | 0.4634 | 14.479 | 8.002 | 0.5318 | 0.00310 | 11251 | |
| | | | | | F Hetero | 3 | 15 | 150.00 | 3.60 | 112.67 | 8.67 | 7.50 | 2.10 | 430.00 | 5.92 | 11.09 | 15.40 | 16.30 | 17.09 | 367.03 | 0.0490 | 1162.83 | 0.4455 | 0.3832 | 193.69 | 4350.36 | 0.1289 | 55.4648 | 0.6597 | 0.3764 | 11.436 | 7.606 | 0.5651 | 0.00191 | 5793 | |
| | | | | 【SD】 | M WT | | | 3.21 | 0.56 | 3.46 | 0.10 | 1.23 | 0.26 | 33.78 | ND | 2.27 | 2.33 | 0.97 | 0.59 | 11.76 | 0.0815 | 10.58 | 0.0746 | 0.0594 | 16.91 | 229.47 | 0.1644 | 0.2811 | 0.3576 | 0.1002 | 1.499 | 0.542 | 0.0522 | 0.00080 | 1465 | |
| | | | | | M Hetero | | | 1.15 | 0.15 | 1.00 | 0.21 | 0.21 | 0.20 | 17.93 | 1.02 | 1.02 | 1.31 | 1.47 | 1.78 | 55.80 | 0.0917 | 11.43 | 0.0485 | 0.0366 | 7.74 | 215.69 | 0.1425 | 8.5323 | 0.1431 | 0.0709 | 2.348 | 0.822 | 0.0803 | 0.00040 | 4772 | |
| | | | | | F WT | | | 1.00 | 0.26 | 1.53 | 0.21 | 0.56 | 0.15 | 95.27 | 0.17 | 0.99 | 1.14 | 1.64 | 1.33 | 38.49 | 0.0178 | 20.04 | 0.0157 | 0.0198 | 3.81 | 134.31 | 0.0300 | 6.1536 | 0.0832 | 0.0475 | 4.687 | 2.230 | 0.1381 | 0.00064 | 5768 | |
| | | | | | F Hetero | | | 2.65 | 0.36 | 0.58 | 0.21 | 0.70 | 0.20 | 50.74 | 0.87 | 1.01 | 1.78 | 1.48 | 1.78 | 18.52 | 0.0216 | 21.25 | 0.0062 | 0.0090 | 3.79 | 103.73 | 0.0612 | 4.8073 | 0.2778 | 0.1963 | 0.352 | 0.530 | 0.1394 | 0.00054 | 1053 | |
| 46 | 21-W494 | *P4hb* | *KTPU8* | 【Mean】 | M WT | 3 | 14-15 | 151.00 | 4.00 | 109.67 | 8.73 | 7.97 | 2.87 | 241.33 | 7.26 | 15.87 | 21.89 | 25.31 | 27.45 | 469.17 | 0.4970 | 1151.77 | 0.6607 | 0.5736 | 233.32 | 5446.24 | 1.1827 | 93.4911 | 1.7313 | 0.1130 | 22.320 | 9.450 | 0.8071 | 0.00556 | 12630 | + |
| | 2084 | 11 | *pU-21W* | | M Hom | 3 | 14-15 | 151.00 | 4.33 | 112.33 | 8.80 | 8.63 | 2.43 | 386.00 | 4.30 | 10.00 | 17.13 | 19.08 | 21.44 | 457.50 | 0.3830 | 1158.00 | 0.5753 | 0.4968 | 224.90 | 4941.89 | 0.7267 | 81.5717 | 1.4224 | 0.1587 | 18.265 | 9.461 | 0.6501 | 0.00330 | 11183 | |
| | | B6;CB-*P4hbGt(pU-21W)494Card* | | | F WT | 3 | 14 | 151.67 | 4.30 | 113.67 | 9.07 | 8.40 | 2.43 | 397.33 | 7.00 | 14.90 | 18.23 | 19.15 | 20.17 | 341.13 | 0.0931 | 1218.07 | 0.5197 | 0.4287 | 206.31 | 4587.91 | 0.2625 | 67.8765 | 0.8528 | 0.2340 | 16.370 | 9.797 | 0.6096 | 0.00277 | 10369 | |
| | | | | | F Hom | 3 | 14-15 | 151.33 | 4.47 | 115.33 | 8.77 | 9.63 | 2.37 | 423.00 | 5.62 | 11.87 | 15.01 | 16.32 | 17.50 | 329.33 | 0.0569 | 1165.93 | 0.4780 | 0.2807 | 149.04 | 4362.56 | 0.1658 | 64.6168 | 0.7988 | 0.2717 | 14.540 | 9.621 | 0.8242 | 0.00262 | 8443 | |
| | | | | 【SD】 | M WT | | | 3.00 | 0.36 | 5.86 | 0.47 | 0.32 | 0.29 | 91.31 | 0.65 | 1.34 | 0.45 | 0.70 | 0.52 | 14.43 | 0.0413 | 11.95 | 0.0291 | 0.0200 | 10.61 | 99.45 | 0.1106 | 3.9669 | 0.0061 | 0.0133 | 1.288 | 0.266 | 0.3676 | 0.00280 | 6128 | |
| | | | | | M Hom | | | 1.00 | 0.67 | 3.06 | 0.36 | 3.37 | 0.15 | 54.56 | 0.44 | 1.38 | 0.64 | 0.01 | 1.17 | 27.01 | 0.2164 | 19.90 | 0.0176 | 0.0100 | 7.32 | 108.21 | 0.5315 | 16.2906 | 0.6030 | 0.0848 | 3.712 | 2.271 | 0.4746 | 0.00131 | 3317 | |
| | | | | | F WT | | | 1.15 | 0.17 | 0.58 | 0.12 | 1.45 | 0.15 | 40.50 | 0.92 | 1.61 | 0.79 | 0.95 | 0.42 | 13.59 | 0.0182 | 80.85 | 0.0327 | 0.0504 | 23.82 | 126.83 | 0.0422 | 2.9504 | 0.0352 | 0.0270 | 0.758 | 0.882 | 0.1167 | 0.00021 | 1164 | |
| | | | | | F Hom | | | 0.58 | 0.61 | 0.58 | 0.40 | 2.42 | 0.25 | 63.24 | 0.75 | 1.03 | 0.43 | 0.48 | 0.57 | 5.71 | 0.0117 | 2.40 | 0.0204 | 0.2409 | 111.41 | 99.85 | 0.0489 | 1.0007 | 0.1121 | 0.0720 | 0.655 | 0.837 | 0.0511 | 0.00033 | 2738 | |
| 47 | 21-W536 | *Osbpl8* | *KTPU8* | 【Mean】 | M WT | 3 | 14 | 149.67 | 4.50 | 111.67 | 9.10 | 7.80 | 2.73 | 342.67 | 6.43 | 13.32 | 20.42 | 23.55 | 25.09 | 432.70 | 0.2895 | 1204.47 | 0.6231 | 0.5176 | 233.28 | 4997.31 | 0.6685 | 65.9696 | 1.8142 | 0.0804 | 19.547 | 9.863 | 0.6567 | 0.00367 | 11984 | + |
| | 2018 | 10 | *pU-21W* | | M Hom | 3 | 14 | 151.00 | 4.63 | 112.00 | 8.77 | 8.97 | 2.63 | 295.67 | 5.01 | 10.07 | 19.60 | 22.18 | 25.14 | 494.47 | 0.5212 | 1173.30 | 0.5836 | 0.4971 | 216.25 | 5083.87 | 1.0885 | 73.3096 | 2.2877 | 0.0572 | 17.768 | 8.784 | 0.4364 | 0.00311 | 11438 | |
| | | B6;CB-*Osbpl8Gt(pU-21W)536Card* | | | F WT | 3 | 14 | 151.33 | 4.23 | 115.33 | 8.93 | 9.10 | 2.50 | 485.33 | 5.46 | 10.39 | 16.86 | 18.53 | 19.62 | 419.97 | 0.1734 | 1192.27 | 0.5390 | 0.4521 | 213.61 | 4707.01 | 0.4014 | 64.2167 | 1.2223 | 0.1681 | 17.557 | 10.099 | 0.5012 | 0.00328 | 10376 | |
| | | | | | F Hom | 3 | 14 | 150.67 | 4.17 | 114.00 | 9.10 | 9.03 | 2.43 | 497.00 | 4.85 | 9.82 | 16.21 | 16.82 | 18.16 | 403.43 | 0.1021 | 1213.77 | 0.4649 | 0.3832 | 209.09 | 4146.06 | 0.2462 | 61.3995 | 1.0012 | 0.1849 | 14.950 | 10.940 | 0.4491 | 0.00234 | 10986 | |
| | | | | 【SD】 | M WT | | | 3.06 | 0.30 | 1.53 | 0.44 | 1.73 | 0.15 | 27.65 | 1.82 | 3.97 | 1.68 | 0.64 | 0.42 | 41.33 | 0.0430 | 18.80 | 0.0246 | 0.0269 | 1.10 | 244.61 | 0.0440 | 2.7333 | 0.1404 | 0.0249 | 0.637 | 0.659 | 0.1884 | 0.00013 | 1521 | |
| | | | | | M Hom | | | 2.65 | 0.15 | 1.00 | 0.15 | 1.63 | 0.06 | 72.04 | 0.31 | 0.97 | 1.03 | 0.76 | 1.13 | 30.50 | 0.1552 | 51.69 | 0.0461 | 0.0242 | 28.45 | 394.31 | 0.3797 | 2.2221 | 0.5514 | 0.0384 | 1.987 | 2.009 | 0.0784 | 0.00075 | 3572 | |
| | | | | | F WT | | | 1.15 | 0.72 | 2.08 | 0.12 | 1.85 | 0.17 | 40.10 | 0.69 | 0.25 | 0.06 | 1.00 | 0.95 | 46.28 | 0.0937 | 12.45 | 0.0160 | 0.0140 | 9.06 | 212.04 | 0.1896 | 5.2913 | 0.4037 | 0.0917 | 2.802 | 2.610 | 0.0483 | 0.00040 | 3728 | |
| | | | | | F Hom | | | 2.08 | 0.23 | 1.73 | 0.60 | 1.19 | 0.06 | 189.64 | ND | 0.29 | ND | 0.83 | 1.02 | 29.41 | 0.0575 | 21.29 | 0.0276 | 0.0266 | 5.76 | 256.61 | 0.1233 | 3.6540 | 0.1875 | 0.0384 | 1.206 | 0.486 | 0.0496 | 0.00080 | 3109 | |
| 48 | 21-W560 | *Rbm12* | *KTPU8* | 【Mean】 | M WT | 3 | 14 | 151.33 | 4.10 | 113.00 | 8.67 | 7.27 | 2.57 | 310.33 | 6.24 | 12.87 | 21.00 | 24.18 | 27.05 | 420.20 | 0.5373 | 1192.23 | 0.6387 | 0.5349 | 228.70 | 5181.08 | 1.2858 | 92.6599 | 1.9839 | 0.0627 | 20.927 | 9.148 | 0.8624 | 0.00402 | 10363 | + |
| | 1914 | 2 | *pU-21W* | | M Hom | 3 | 14-15 | 152.33 | 4.60 | 114.67 | 8.60 | 8.20 | 2.67 | 330.33 | 6.16 | 12.65 | 20.28 | 23.11 | 25.49 | 427.83 | 0.3827 | 1176.97 | 0.6660 | 0.5658 | 240.84 | 5270.51 | 0.9000 | 88.4055 | 1.8878 | 0.0903 | 20.265 | 9.146 | 0.6274 | 0.00363 | 10737 | |
| | | B6;CB-*Rbm12Gt(pU-21W)560Card* | | | F WT | 3 | 14 | 151.00 | 4.07 | 115.00 | 8.73 | 6.83 | 2.47 | 393.33 | 6.62 | 12.43 | 18.34 | 19.93 | 22.32 | 307.43 | 0.0627 | 1162.63 | 0.5077 | 0.4366 | 211.09 | 4603.25 | 0.1959 | 64.4255 | 0.7921 | 0.2469 | 16.025 | 9.507 | 0.6392 | 0.00266 | 12004 | |
| | | | | | F Hom | 3 | 14 | 150.67 | 4.23 | 115.00 | 8.80 | 7.53 | 2.13 | 415.00 | 5.30 | 11.23 | 16.95 | 19.09 | 20.95 | 337.97 | 0.0903 | 1202.17 | 0.4367 | 0.3642 | 175.77 | 4467.96 | 0.2609 | 68.1702 | 1.0242 | 0.1826 | 13.618 | 8.628 | 0.6687 | 0.00219 | 6764 | |
| | | | | 【SD】 | M WT | | | 1.53 | 0.17 | 2.65 | 0.06 | 0.50 | 0.21 | 25.42 | 0.74 | 1.17 | 1.04 | 1.05 | 1.47 | 26.07 | 0.1045 | 24.70 | 0.0871 | 0.0624 | 22.91 | 146.35 | 0.1857 | 9.7425 | 0.0728 | 0.0187 | 5.361 | 2.616 | 0.1257 | 0.00028 | 3496 | |
| | | | | | M Hom | | | 0.58 | 0.72 | 1.53 | 0.46 | 0.79 | 0.06 | 50.82 | 0.40 | 3.08 | 1.33 | 1.76 | 1.80 | 12.76 | 0.0021 | 11.16 | 0.0312 | 0.0237 | 6.06 | 167.57 | 0.0234 | 2.3128 | 0.1333 | 0.0228 | 3.159 | 1.081 | 0.0872 | 0.00044 | 3901 | |
| | | | | | F WT | | | 1.73 | 0.60 | 2.65 | 0.45 | 0.64 | 0.12 | 22.03 | 0.47 | 1.31 | 1.22 | 0.69 | 1.36 | 7.03 | 0.0162 | 18.55 | 0.0240 | 0.0138 | 11.01 | 49.38 | 0.0482 | 1.0492 | 0.1842 | 0.0379 | 0.182 | 0.298 | 0.0949 | 0.00013 | 1735 | |
| | | | | | F Hom | | | 0.58 | 0.06 | 1.00 | 0.17 | 0.74 | 0.15 | 37.36 | 0.74 | 1.50 | 2.73 | 4.21 | 4.81 | 0.38 | 0.0086 | 65.56 | 0.0294 | 0.0330 | 24.35 | 330.26 | 0.0232 | 2.9876 | 0.1378 | 0.0573 | 2.150 | 1.629 | 0.0591 | 0.00072 | 1451 | |
| 49 | 21-W588 | *Itpr1* | *KTPU8* | 【Mean】 | M WT | 4 | 15 | 149.50 | 4.48 | 108.75 | 9.23 | 7.95 | 2.75 | 276.00 | 8.67 | 14.82 | 26.33 | 29.78 | 32.10 | 526.08 | 0.5962 | 1205.20 | 0.7284 | 0.6042 | 241.29 | 5566.45 | 1.1550 | 80.8885 | 1.7274 | 0.1340 | 20.270 | 8.180 | 1.1587 | 0.00482 | 4717 | + |
| | 1834 | 6 | *pU-21W* | | M Hetero | 4 | 14-15 | 149.00 | 4.38 | 109.50 | 9.15 | 8.33 | 2.55 | 340.75 | 6.71 | 12.59 | 20.71 | 23.76 | 26.15 | 487.60 | 0.4454 | 1168.50 | 0.6230 | 0.5330 | 224.44 | 5240.43 | 0.9023 | 72.9174 | 1.6574 | 0.1525 | 17.376 | 8.092 | 1.3222 | 0.00471 | 5010 | |
| | | B6;CB-*Itpr1Gt(pU-21W)588Card* | | | F WT | 3 | 14-15 | 150.00 | 4.23 | 114.33 | 9.27 | 7.77 | 2.37 | 476.33 | 9.12 | 14.63 | 18.81 | 20.77 | 21.03 | 414.33 | 0.0720 | 1194.53 | 0.5318 | 0.4452 | 217.21 | 4588.42 | 0.1677 | 60.5739 | 0.6175 | 0.4216 | 15.020 | 8.954 | 1.3913 | 0.00438 | 4422 | |
| | | | | | F Hetero | 3 | 14-15 | 149.75 | 4.13 | 114.25 | 9.13 | 8.23 | 2.15 | 455.75 | 9.05 | 15.18 | 20.65 | 23.81 | 25.71 | 443.40 | 0.0658 | 1212.67 | 0.5755 | 0.4744 | 226.66 | 4718.85 | 0.1505 | 63.9529 | 0.5194 | 0.4362 | 17.278 | 10.112 | 1.1087 | 0.00408 | 4855 | |
| | | | | 【SD】 | M WT | | | 0.58 | 0.26 | 2.87 | 0.21 | 0.90 | 0.26 | 52.26 | 0.28 | 0.37 | 1.44 | 2.06 | 2.29 | 19.51 | 0.3024 | 18.97 | 0.0796 | 0.0643 | 5.41 | 443.01 | 0.5925 | 3.7586 | 0.6613 | 0.0909 | 4.275 | 1.078 | 0.2181 | 0.00146 | 432 | |
| | | | | | M Hetero | | | 1.41 | 0.49 | 1.73 | 0.31 | 0.79 | 0.26 | 88.09 | 1.95 | 3.66 | 3.15 | 4.31 | 5.70 | 44.57 | 0.2828 | 12.76 | 0.0979 | 0.0830 | 10.42 | 636.81 | 0.5395 | 7.3793 | 0.4146 | 0.0728 | 1.837 | 1.371 | 0.1379 | 0.00093 | 1000 | |
| | | | | | F WT | | | 1.00 | 0.12 | 1.15 | 0.21 | 0.29 | 0.21 | 76.23 | 1.92 | 2.46 | 1.74 | 1.04 | ND | 34.12 | 0.0431 | 8.90 | 0.0283 | 0.0241 | 8.48 | 103.10 | 0.0918 | 5.6792 | 0.1466 | 0.0991 | 1.463 | 0.618 | 0.0719 | 0.00089 | 1154 | |
| | | | | | F Hetero | | | 1.26 | 0.25 | 1.71 | 0.29 | 0.91 | 0.24 | 108.89 | 0.95 | 1.37 | 1.00 | 2.05 | 3.00 | 44.63 | 0.0292 | 16.03 | 0.0328 | 0.0220 | 10.52 | 44.43 | 0.0754 | 3.6332 | 0.2651 | 0.2133 | 1.148 | 0.775 | 0.0861 | 0.00030 | 827 | |
| 50 | 21-W6 | *Ctnnd1* | *KTPU8* | 【Mean】 | M WT | 3 | 14-15 | 151.33 | 3.77 | 113.67 | 9.70 | 5.97 | 2.17 | 280.67 | 11.98 | 18.54 | 24.13 | 26.34 | 26.94 | 513.93 | 0.2649 | 1296.00 | 0.7420 | 0.5718 | 265.71 | 4976.70 | 0.5197 | 73.5867 | 1.3526 | 0.1713 | 26.895 | 13.661 | 0.4458 | 0.00445 | 18874 | + |
| | 1304 | 2 | *pU-21W* | | M Hetero | 3 | 15 | 150.33 | 4.23 | 112.33 | 9.90 | 6.00 | 2.10 | 353.67 | ND | 12.25 | 22.08 | 25.14 | 26.12 | 475.40 | 0.2291 | 1248.83 | 0.5933 | 0.4751 | 231.37 | 4666.49 | 0.4832 | 67.5102 | 1.5548 | 0.1289 | 21.707 | 12.384 | 0.8246 | 0.00350 | 15329 | |
| | | B6;CB-*Ctnnd1Gt(pU-21W)6Card* | | | F WT | 3 | 14-15 | 151.50 | 3.60 | 114.50 | 9.40 | 5.13 | 1.90 | 387.75 | 8.62 | 16.62 | 22.17 | 23.72 | 25.02 | 470.28 | 0.1518 | 1235.00 | 0.5816 | 0.4707 | 216.25 | 4820.68 | 0.3119 | 64.1814 | 1.0714 | 0.1765 | 17.118 | 9.572 | 0.9115 | 0.00268 | 10216 | |
| | | | | | F Hetero | 3 | 14-15 | 152.00 | 4.07 | 117.00 | 8.57 | 5.87 | 2.13 | 415.33 | 7.78 | 13.31 | 19.65 | 19.92 | 21.27 | 453.70 | 0.1170 | 1276.17 | 0.5419 | 0.4245 | 229.40 | 4255.50 | 0.2555 | 62.8722 | 1.1559 | 0.1320 | 18.125 | 11.522 | 0.8212 | 0.00252 | 13288 | |
| | | | | 【SD】 | M WT | | | 0.58 | 0.15 | 1.15 | 0.72 | 1.35 | 0.31 | 25.42 | ND | 1.63 | 1.21 | 1.51 | 4.00 | 20.73 | 0.1432 | 23.88 | 0.0939 | 0.0627 | 19.02 | 241.81 | 0.2768 | 7.4021 | 0.5284 | 0.1277 | 4.323 | 2.030 | 0.0696 | 0.00087 | 3213 | |
| | | | | | M Hetero | | | 1.15 | 0.57 | 1.53 | 0.42 | 0.46 | 0.17 | 34.70 | ND | 4.54 | 4.42 | 6.08 | 3.03 | 16.03 | 0.1512 | 31.50 | 0.0599 | 0.0451 | 15.93 | 311.44 | 0.3123 | 3.9704 | 0.5725 | 0.1117 | 4.794 | 1.271 | 0.1317 | 0.00074 | 2327 | |
| | | | | | F WT | | | 2.08 | 0.56 | 2.65 | 0.24 | 0.67 | 0.32 | 125.08 | 2.35 | 1.78 | 0.82 | 0.26 | 1.08 | 50.59 | 0.1210 | 15.39 | 0.0568 | 0.0422 | 16.12 | 193.04 | 0.2199 | 7.1712 | 0.4956 | 0.0944 | 1.025 | 1.149 | 0.1166 | 0.00024 | 3412 | |
| | | | | | F Hetero | | | 1.00 | 0.50 | 0.00 | 1.71 | 1.03 | 0.21 | 171.51 | ND | 0.29 | 0.88 | 1.22 | 1.45 | 28.38 | 0.0635 | 10.74 | 0.0732 | 0.0551 | 19.74 | 319.83 | 0.1250 | 3.6980 | 0.2278 | 0.0438 | 3.731 | 1.734 | 0.1992 | 0.00066 | 1072 | |
| 51 | 21-W76 | *Srr* | *KTPU8* | 【Mean】 | M WT | 4 | 14-15 | 150.25 | 3.78 | 110.75 | 9.40 | 5.23 | 2.18 | 267.75 | 7.19 | 18.60 | 23.68 | 26.56 | 28.64 | 507.48 | 0.4105 | 1263.48 | 0.7471 | 0.5909 | 268.85 | 5105.46 | 0.8013 | 73.1987 | 1.8104 | 0.0864 | 24.743 | 11.872 | 0.4368 | 0.00563 | 14810 | − |
| | 1439 | 11 | *pU-21W* | | M Homo | 3 | 14-15 | 152.00 | 3.63 | 113.33 | 9.40 | 7.27 | 1.97 | 300.67 | 5.78 | 12.60 | 20.19 | 24.25 | 25.93 | 503.83 | 0.3944 | 1251.57 | 0.7207 | 0.5754 | 245.36 | 5298.81 | 0.7980 | 74.8330 | 1.7021 | 0.1701 | 23.072 | 10.290 | 0.4958 | 0.00544 | 9826 | |
| | | B6;CB-*SrrGt(pU-21W)76Card* | | | F WT | 3 | 14 | 152.33 | 3.67 | 117.00 | 9.80 | 6.40 | 2.17 | 546.00 | 7.30 | 15.64 | 18.57 | 20.29 | 21.46 | 434.10 | 0.0834 | 1274.97 | 0.6582 | 0.5162 | 244.35 | 4829.64 | 0.1880 | 63.5783 | 0.6995 | 0.3063 | 21.448 | 11.557 | 0.3191 | 0.00363 | 15001 | |
| | | | | | F Homo | 4 | 15 | 153.00 | 3.55 | 113.25 | 9.23 | 4.58 | 2.10 | 497.25 | 7.00 | 14.63 | 17.67 | 18.86 | 20.11 | 424.75 | 0.1034 | 1284.13 | 0.6273 | 0.4885 | 241.72 | 4659.04 | 0.2285 | 62.9396 | 0.8652 | 0.2297 | 18.793 | 10.885 | 0.3351 | 0.00289 | 14152 | |
| | | | | 【SD】 | M WT | | | 0.96 | 0.75 | 1.71 | 0.08 | 1.76 | 0.28 | 29.51 | 1.20 | 3.16 | 1.95 | 0.88 | 0.94 | 71.80 | 0.1733 | 10.34 | 0.1250 | 0.0951 | 27.85 | 346.63 | 0.2213 | 8.7606 | 0.0994 | 0.0150 | 3.920 | 0.532 | 0.0372 | 0.00217 | 991 | |
| | | | | | M Homo | | | 1.00 | 0.12 | 1.15 | 0.36 | 1.55 | 0.35 | 79.61 | 0.39 | 1.66 | 0.13 | 0.41 | 0.39 | 39.88 | 0.0649 | 17.82 | 0.0645 | 0.0436 | 21.93 | 65.88 | 0.1255 | 7.8409 | 0.1260 | 0.0154 | 5.324 | 2.108 | 0.1538 | 0.00173 | 4919 | |
| | | | | | F WT | | | 1.53 | 0.50 | 1.00 | 0.46 | 1.44 | 0.15 | 95.77 | 0.12 | 0.56 | 1.16 | 0.88 | 0.44 | 53.84 | 0.0148 | 1.53 | 0.0400 | 0.0318 | 8.35 | 141.15 | 0.0102 | 6.1031 | 0.0692 | 0.0704 | 1.444 | 0.696 | 0.0232 | 0.00024 | 475 | |
| | | | | | F Homo | | | 2.16 | 0.48 | 1.71 | 0.21 | 1.44 | 0.16 | 146.04 | 0.26 | 1.65 | 0.48 | 0.68 | 0.55 | 58.68 | 0.0827 | 10.39 | 0.0530 | 0.0402 | 10.95 | 208.99 | 0.1567 | 6.8317 | 0.3509 | 0.0849 | 2.256 | 1.230 | 0.0747 | 0.00033 | 1717 | |
| 52 | R | *RhoA* | *KTPU10* | 【Mean】 | M WT | 8 | 14-15 | 150.50 | 4.23 | 110.63 | 9.03 | 7.83 | 2.69 | 293.00 | 6.98 | 14.95 | 21.74 | 24.54 | 26.29 | 473.78 | 0.3131 | 1152.40 | 0.5632 | 0.4887 | 209.19 | 5099.02 | 0.6586 | 70.3046 | 1.5348 | 0.1276 | 18.486 | 8.842 | 0.9759 | 0.00471 | 6974 | − |
| | - | 9 | *pU-17* | | M Hetero | 6 | 14-15 | 150.83 | 4.65 | 110.67 | 9.00 | 7.58 | 2.48 | 326.50 | 6.56 | 13.60 | 21.20 | 23.69 | 25.97 | 429.83 | 0.2283 | 1138.48 | 0.4989 | 0.4378 | 196.31 | 4816.94 | 0.5055 | 64.4298 | 1.3489 | 0.1858 | 14.334 | 7.747 | 1.0689 | 0.00321 | 5318 | |
| | | B6;CB-*RhoaGt(pU-17)52Imeg* | | | F WT | 8 | 14-16 | 149.63 | 3.88 | 113.25 | 9.11 | 6.79 | 2.23 | 398.75 | 7.20 | 15.40 | 18.98 | 20.96 | 22.01 | 379.36 | 0.0845 | 1177.68 | 0.5273 | 0.4477 | 207.53 | 4754.78 | 0.2166 | 55.2503 | 0.8583 | 0.2681 | 17.178 | 9.573 | 0.9643 | 0.00356 | 8495 | |
| | | | | | F Hetero | 9 | 14-16 | 149.67 | 4.41 | 112.67 | 8.86 | 7.33 | 2.31 | 427.33 | 6.66 | 13.53 | 16.98 | 19.09 | 20.74 | 362.79 | 0.0788 | 1172.74 | 0.5042 | 0.4298 | 206.01 | 4614.96 | 0.2078 | 53.3771 | 0.8331 | 0.2913 | 18.093 | 10.528 | 0.9879 | 0.00388 | 8654 | |
| | | | | 【SD】 | M WT | | | 1.77 | 0.48 | 1.60 | 0.21 | 1.50 | 0.24 | 41.95 | 1.49 | 3.32 | 2.67 | 2.54 | 0.73 | 50.10 | 0.0832 | 11.71 | 0.0470 | 0.0387 | 9.61 | 229.30 | 0.1413 | 8.1415 | 0.2962 | 0.0767 | 4.930 | 1.648 | 0.2698 | 0.00195 | 3400 | |
| | | | | | M Hetero | | | 1.17 | 0.19 | 2.50 | 0.28 | 0.40 | 0.25 | 50.33 | 2.00 | 4.08 | 2.56 | 2.92 | 2.65 | 58.87 | 0.1508 | 18.06 | 0.0967 | 0.0817 | 20.91 | 489.88 | 0.2825 | 8.2333 | 0.3684 | 0.0758 | 3.149 | 1.114 | 0.1285 | 0.00059 | 2658 | |
| | | | | | F WT | | | 1.69 | 0.25 | 1.58 | 0.26 | 0.79 | 0.25 | 31.13 | 0.95 | 1.19 | 1.43 | 1.54 | 2.19 | 14.38 | 0.0208 | 11.63 | 0.0337 | 0.0271 | 7.38 | 163.02 | 0.0509 | 2.6630 | 0.0935 | 0.0512 | 0.866 | 0.767 | 0.2312 | 0.00055 | 2311 | |
| | | | | | F Hetero | | | 1.50 | 0.32 | 1.00 | 0.29 | 0.62 | 0.26 | 30.67 | 0.55 | 0.87 | 0.95 | 1.21 | 1.66 | 26.12 | 0.0263 | 13.56 | 0.0326 | 0.0243 | 10.80 | 155.87 | 0.0612 | 3.6732 | 0.1660 | 0.0766 | 3.196 | 1.753 | 0.1750 | 0.00067 | 4038 | |

EGTC: Exchangeable Gene Trap Clones, CARD: Center for Animal Resources and Development of Kumamoto University, Chr: Chromosome, GT: gene trap mouse, Tb.BMD: trabecular bone mineral density, Tb.BMC: trabecular bone mineral content, Ct.BMD: cortical bone mineral density, Ct.BMC: cortical bone mineral content, Ct.V: cortical bone volume, Ct.Th: cortical bone thickness, Ex.Ln.Le: external line length, BV: trabecular bone volume, Tb.Th: trabecular bone thickness, Tb.N: trabecular bone number, V*m.space: marrow space star volume, M.load: maximum load, M.stress: maximum stress, M.dsp: maximum displacement, M.work: maximum work, E.M: elastic modulus. ND: not determined, +: positive X-gal staining, −: unchanged data, Red: > [WT mean + 2.0 SD], Blue: < [WT mean − 2.0 SD], WT: wild-type. "R" line has not been registered in the EGTC database.

**Supplementary Table S3. Correlation between the first screening items and the second screening results (BMA or BSA) in female trap lines**

| | 1st screening selected lines | 2nd screening positive lines | BSA positive lines | BMA positive lines | 2nd screening negative lines | P-value |
|---|---|---|---|---|---|---|
| **EST profile** | 30 | 26 | 19 (73.1) | 22 (84.6) | 4 | 0.4588 |
| **X-gal** | 16 | 15 | 12 (80.0) | 13 (86.7) | 1 | 0.2177 |
| **RA** | 8 | 6 | 4 (66.7) | 4 (66.7) | 2 | 0.3571 |
| **Novel gene** | 11 | 8 | 5 (62.5) | 6 (75.0) | 3 | 0.2158 |

Biomechanical strength analysis (BSA) and bone morphometric analysis (BMA) indicate the number of positive lines (%). One-tailed Fisher's exact test was calculated for lines with a difference observed for each selected item in the first and second screening (BMA or BSA). EST: expressed sequence tag, RA: related article, Novel gene: EST+New.

**Supplementary Table S4. Correlation coefficient between the gene trap and wild-type mouse ratio (GT/WT) of M. load and morphometric parameters in male trap lines**

| BMD analysis | Correlation coefficient | P-value |
|---|---|---|
| Tb.BMD [mg/cm$^3$] | 0.595 | $5.48\times10^{-6}$ |
| Tb.BMC [mg] | 0.677 | $9.4\times10^{-8}$ |
| Ct.BMD [mg/cm$^3$] | 0.416 | 0.0025 |
| Ct.BMC [mg] | 0.730 | $3.25\times10^{-9}$ |
| **Cortical bone analysis** | | |
| Ct.V [mm$^3$] | 0.705 | $1.71\times10^{-8}$ |
| Ct.Th [µm] | 0.559 | $2.51\times10^{-5}$ |
| Ex.Ln.Le [µm] | 0.599 | $4.65\times10^{-6}$ |
| **Trabecular bone analysis** | | |
| BV [mm$^3$] | 0.691 | $4.04\times10^{-8}$ |
| Tb.Th [µm] | 0.632 | $1.00\times10^{-6}$ |
| Tb.N [1/mm] | 0.592 | $6.39\times10^{-6}$ |
| V*m.space [mm$^3$] | -0.411 | $1.25\times10^{-3}$ |

Tb.BMD: trabecular bone mineral density, Tb.BMC: trabecular bone mineral content, Ct.BMD: cortical bone mineral density, Ct.BMC: cortical bone mineral content, Ct.V: cortical bone volume, Ct.Th: cortical bone thickness, Ex.Ln.Le: external line length, BV: trabecular bone volume, Tb.Th: trabecular bone thickness, Tb.N: trabecular bone number, V*m.space: marrow space star volume.

**Supplementary Table S5. Real-time PCR primers**

| | |
|---|---|
| *BMP2* | Forward 5′-CGGACTGCGGTCTCCTAA-3′<br>Reverse 5′-GGGGAAGCAGCAACACTAGA-3’ |
| *Runx2* | Forward 5′-TACAAACCATACCCAGTCCCTGTTT-3′<br>Reverse 5′-AGTGCTCTAACCACAGTCCATGCA-3′ |
| *Osterix* | Forward 5′-ATGGCGTCCTCTCTGCTTG-3′<br>Reverse 5′-TGAAAGGTCAGCGTATGGCTT-3′ |
| *ALP* | Forward 5′-CCAACTCTTTTGTGCCAGAGA-3′<br>Reverse 5′-GGCTACATTGGTGTTGAGCTTTT-3′ |
| *Col1a1* | Forward 5′-GCTCCTCTTAGGGGCCACT-3′<br>Reverse 5′-CCACGTCTCACCATTGGGG-3′ |
| *OCN* | Forward 5′-AGACAAGTCCCACACAGCAG-3′<br>Reverse 5′-GTCAGAGAGACAGAGCGCAG-3′ |
| *RANKL* | Forward 5′-CTCAACAAGGATACGGTGTGC-3′<br>Reverse 5′-ACCACATCTGATTCCGTTGTC-3′ |
| *NFATc1* | Forward 5′-GACCCGGAGTTCGACTTCG-3′<br>Reverse 5′-TGACACTAGGGGACACATAACTG-3′ |
| *TRAP* | Forward 5′-TTCACTGGAGTGCACGATG-3′<br>Reverse 5′-ATGCAATCTGTGCAGAGACG-3′ |
| *β-actin* | Forward 5′-GAGCTATGAGCTGCCTGACG-3′<br>Reverse 5′-AGTTTCATGGATGCCACAGG-3′ |

*BMP2*: bone morphogenetic protein 2, *Runx2*: runt-related transcription factor 2, *ALP*: alkaline phosphatase, *Col1a1*: collagen, type I, alpha 1, *OCN*: osteocalcin, *RANKL*: receptor activator of nuclear factor kappa-B ligand, *NFATc1*: nuclear factor of activated T-cells, cytoplasmic 1, *TRAP*: tartrate-resistant acid phosphatase.
